# Association between early post-insertion arterial oxygen tension and documented acute upper-body deep or central venous thrombosis in critically ill adults: A retrospective MIMIC-IV cohort study

**DOI:** 10.1371/journal.pone.0359719

**Published:** 2026-10-05

**Authors:** Qing Cui, Anshuai Fang, Haiyan Zhang, Qingtong Meng, Sijia Zhou, Yueming Peng, Weixiang Luo

**Affiliations:** 1 Department of Pulmonary and Critical Care Medicine, Shenzhen Key Laboratory of Respiratory Diseases, Shenzhen Clinical Research Center for Respiratory Disease, Shenzhen Institute of Respiratory Diseases, Shenzhen People's Hospital (The First Affiliated Hospital, Southern University of Science and Technology, The Second Clinical Medical College, Jinan University), Shenzhen, China; 2 Department of Hepatobiliary Surgery, Shenzhen People’s Hospital, Shenzhen, Guangdong, China; 3 Department of Cardiology, Shenzhen People’s Hospital, Shenzhen, Guangdong, China; 4 Department of Nursing, Shenzhen People’s Hospital, Shenzhen, Guangdong, China; Sapienza University of Rome: Universita degli Studi di Roma La Sapienza, ITALY

## Abstract

Abnormal oxygenation may influence thrombosis, but evidence in catheterized critically ill patients remains limited. We examined whether the first arterial partial pressure of oxygen (PaO_2_) measured within 24 hours after qualifying invasive venous catheter insertion was associated with documented acute upper-body deep or central venous thrombosis during hospitalization. This retrospective observational cohort study used the Medical Information Mart for Intensive Care IV database, version 2.2 (MIMIC-IV v2.2) (2008–2019). Adult intensive care unit admissions were included when a qualifying invasive venous catheter was present and an arterial PaO_2_ measurement was obtained after insertion, within 24 hours, and while the catheter remained in situ. PaO_2_ was analyzed continuously and categorically (≤80, 81–100, and >100 mmHg). Logistic regression models sequentially adjusted for patient characteristics, laboratory measurements, catheter characteristics, measurement timing, inspired oxygen concentration, respiratory support, and non-respiratory organ dysfunction. Multiple imputation addressed missing covariate data, and patient-clustered robust standard errors accounted for repeated admissions. The cohort included 11,277 hospital admissions from 10,789 patients; 195 admissions (1.73%) had the primary outcome. In the unadjusted model, higher PaO_2_ was associated with lower odds of the outcome (odds ratio [OR] per 10-mmHg increase, 0.970; 95% confidence interval [CI], 0.952–0.987), but this association attenuated after adjustment. In the fully adjusted model, PaO_2_ was not associated with the outcome (OR, 1.008; 95% CI, 0.989–1.027; P = 0.430). Most secondary analyses supported the primary result, although the 60-minute sensitivity analysis yielded adjustment-dependent estimates. In the complete cohort, a single early post-insertion PaO_2_ measurement was not independently associated with documented acute upper-body deep or central venous thrombosis. These findings do not support PaO_2_-based thrombosis risk stratification or modification of oxygen therapy for thrombosis prevention. Future studies should incorporate repeated oxygenation measurements and accurately timed, imaging-confirmed thrombotic outcomes.

## Introduction

Central venous access devices are widely used in critically ill patients for the administration of vasoactive medications, fluids, parenteral nutrition, renal replacement therapy, and other supportive treatments. However, upper-extremity deep or central venous thrombosis is an important complication of vascular access and may lead to catheter dysfunction, treatment interruption, recurrent venous thromboembolism, pulmonary embolism, and post-thrombotic morbidity [1–4]. Reported thrombosis rates vary substantially according to the population studied, catheter type, outcome definition, follow-up duration, and whether systematic imaging surveillance is performed. Peripherally inserted central catheters (PICCs) have been associated with a higher occurrence of venous thromboembolism than conventional central venous catheters in several observational syntheses, particularly in critically ill populations, although findings have not been uniform across study designs [2,3]. Thrombotic susceptibility in the ICU is also influenced by patient and treatment characteristics, including malignancy, sepsis, invasive mechanical ventilation, vasoactive medication use, immobility, and the presence of central venous catheters [5]. In addition, catheter diameter, the catheter-to-vein relationship, tip position, insertion technique, and other device-related factors may modify thrombotic risk [6].

Abnormal oxygenation may also be relevant to thrombosis, but the direction and magnitude of the relationship remain uncertain. Recurrent hypoxia–reoxygenation, as observed in obstructive sleep apnea, has been linked to oxidative stress, systemic inflammation, endothelial dysfunction, altered blood viscosity, and increased expression of procoagulant mediators, providing a potential connection between oxygen disturbance and the components of Virchow’s triad [7]. However, a systematic review of environmental hypoxia in healthy adults found only small and heterogeneous changes in coagulation, platelet activity, and fibrinolysis and did not establish hypoxia as an independent cause of hypercoagulability [8]. Disease-specific clinical studies have reported lower arterial partial pressure of oxygen (PaO_2_) among patients with venous thromboembolism or pulmonary embolism in chronic obstructive pulmonary disease [9,10]. These findings may nevertheless reflect respiratory failure, inflammation, immobility, invasive ventilation, or the effects of pulmonary embolism on gas exchange rather than an isolated effect of low PaO_2_. At the opposite end of the oxygen spectrum, exposure to higher inspired oxygen concentrations may increase oxidative stress and reduce antioxidant activity [11]. Trials of higher versus lower oxygenation targets in mechanically ventilated ICU patients have also used heterogeneous and overlapping target ranges, leaving uncertainty regarding the clinical effects of different oxygen exposures [12]. Consistent with this uncertainty, large randomized trials comparing lower or conservative oxygenation strategies with higher or usual oxygenation strategies have not demonstrated a consistent clinical advantage of one strategy across critically ill populations [13–15]. Thus, physiological or clinical observations arising from intermittent hypoxia, environmental hypoxia, chronic lung disease, or administered oxygen strategies cannot be directly extrapolated to a single arterial PaO_2_ measurement obtained during routine critical care.

In critically ill patients, PaO_2_ is influenced by inspired oxygen concentration, ventilatory support, pulmonary gas exchange, perfusion, disease severity, and the timing of arterial blood-gas sampling. Many of these factors are also related to the indication for vascular access, catheter selection, and thrombotic risk. Consequently, an apparent association between PaO_2_ and thrombosis may be confounded by the clinical and device-related circumstances surrounding oxygen measurement and catheter placement. Direct evidence examining whether an early arterial PaO_2_ measurement is associated with upper-body venous thrombosis among catheterized ICU patients, after accounting for these factors, remains limited. We therefore conducted a retrospective observational cohort study using the Medical Information Mart for Intensive Care IV database to examine the association between the first arterial PaO_2_ measured within 24 hours after qualifying catheter insertion and documented acute upper-body deep or central venous thrombosis during hospitalization. PaO_2_ was evaluated as both a continuous and categorical exposure, potential nonlinear associations were assessed, and the robustness of the findings was examined after adjustment for catheter characteristics, measurement timing, respiratory support, inspired oxygen concentration, and illness severity.

## Materials and methods

### Study design and objective

This retrospective observational cohort study used deidentified electronic health record data from the Medical Information Mart for Intensive Care IV database and was reported in accordance with the Strengthening the Reporting of Observational Studies in Epidemiology (STROBE) statement. The study aimed to evaluate whether arterial partial pressure of oxygen (PaO_2_) measured during the first 24 hours after qualifying invasive venous catheter insertion was associated with documented acute upper-body deep or central venous thrombosis during the corresponding hospitalization in critically ill adults. We further examined whether this association remained after adjustment for patient characteristics, catheter-related factors, the timing of PaO_2_ measurement, inspired oxygen concentration, respiratory support, and illness severity.

The hospital admission was used as the primary unit of analysis. When more than one ICU stay within the same hospital admission met the eligibility criteria, only the earliest eligible ICU stay was retained. A patient could contribute more than one eligible hospital admission. This study evaluated admission-level associations and was not designed as a time-to-event analysis. Because thrombosis diagnoses lacked reliable onset timestamps, temporal precedence of the selected PaO_2_ measurement could not be confirmed for every admission.

### Data source

Data were obtained from the Medical Information Mart for Intensive Care IV database, version 2.2 (MIMIC-IV v2.2), a deidentified electronic health record database containing clinical information from patients treated at Beth Israel Deaconess Medical Center in Boston, Massachusetts, between 2008 and 2019 [16]. The database includes hospital- and ICU-level information on demographics, hospital and ICU encounters, laboratory measurements, bedside observations, procedures, respiratory support, medications, and diagnosis codes. Access to MIMIC-IV is restricted to credentialed researchers who complete the required human-subjects research training and sign the PhysioNet data use agreement. The authors accessed the database through the standard credentialing process and had no special access privileges.

### Ethics statement

The collection and sharing of the MIMIC-IV research resource were reviewed by the Institutional Review Board of Beth Israel Deaconess Medical Center, which approved the data-sharing initiative and granted a waiver of individual informed consent [16]. The present study was a secondary analysis of deidentified data accessed under the PhysioNet Credentialed Health Data Use Agreement. The investigators had no direct contact with participants and no access to identifiable information. No additional institutional ethics approval was obtained for this analysis because the study used only an existing deidentified database and involved no participant recruitment, intervention, or collection of identifiable information. No additional individual informed consent was obtained.

### Study population

We screened invasive venous catheter records linked to ICU stays in MIMIC-IV. ICU stays were eligible if patients were aged 18 years or older at hospital admission, remained in the ICU for at least 24 hours, had at least one qualifying central venous catheter episode overlapping the ICU stay, and had at least one eligible arterial PaO_2_ measurement obtained after catheter insertion and within 24 hours of catheter placement while at least one qualifying catheter remained in situ.

Catheters documented in lower-body locations were excluded because the study outcomes were restricted to upper-body deep or central venous thrombosis. Catheters documented in upper-body locations were retained. Otherwise eligible catheter records were not excluded solely because their anatomical location was incompletely documented.

The initial extraction included 30,003 invasive venous catheter records corresponding to 18,056 ICU stays among 15,916 patients. After application of the eligibility criteria, 11,474 ICU stays remained. A further 197 ICU stays were excluded because an earlier eligible ICU stay occurred during the same hospital admission. The final cohort comprised 11,277 hospital admissions from 10,789 unique patients. The cohort-selection process is shown in Fig 1.

**Fig 1:**
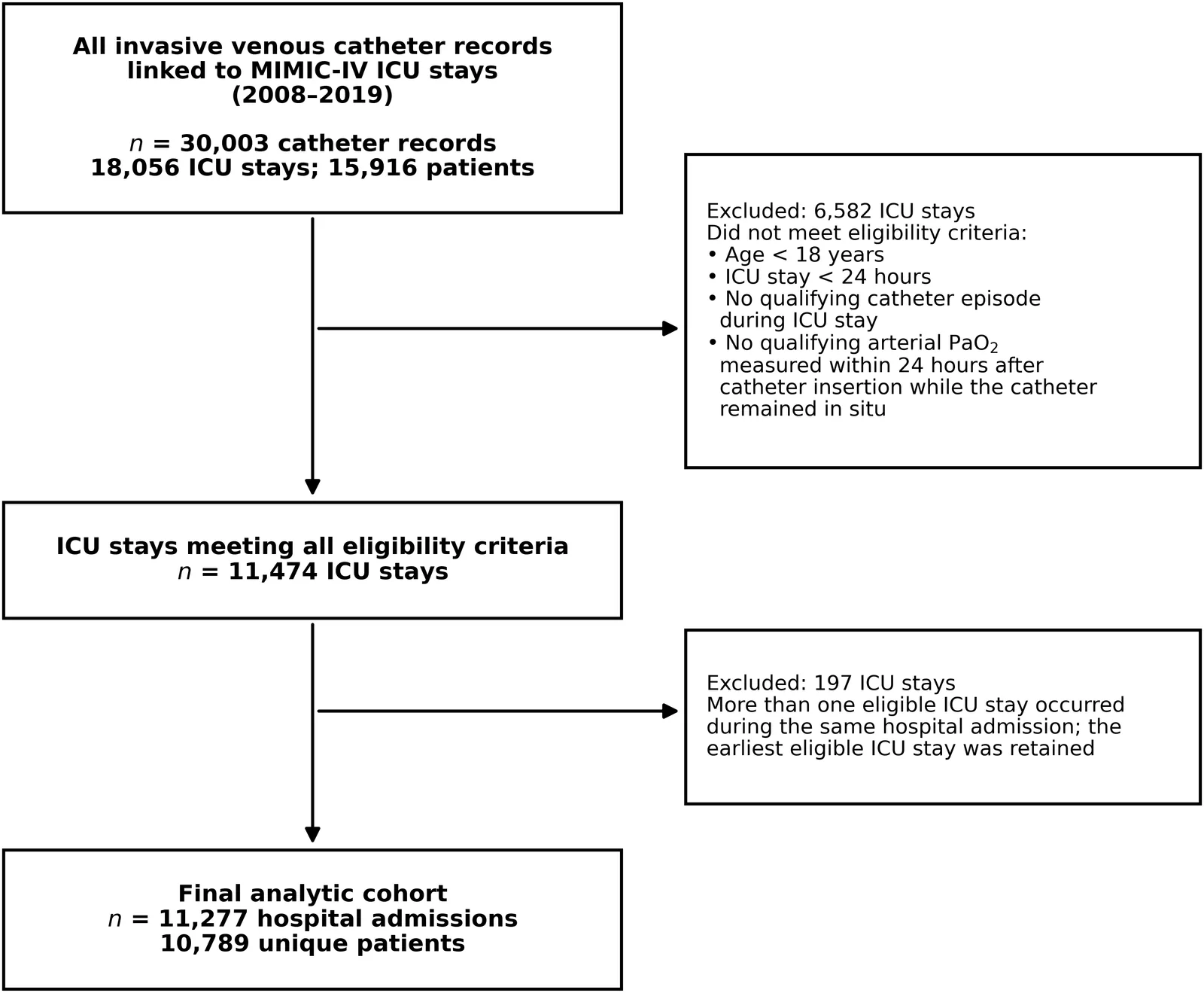
Flowchart of cohort selection. ICU, intensive care unit; PaO_2_, arterial partial pressure of oxygen. The hospital admission was used as the unit of analysis. When more than one eligible ICU stay occurred during the same hospital admission, only the earliest eligible ICU stay was retained. A patient could contribute more than one eligible hospital admission.

### Exposure assessment

The primary exposure was arterial PaO_2_ measured after placement of a qualifying central venous catheter. For each eligible ICU stay, catheter episodes were reconstructed using the documented insertion and removal times. The beginning of the qualifying catheter episode was defined as the index time.

An arterial PaO_2_ measurement was eligible if it was obtained:

1. At or after the index catheter insertion time;
2. Within 24 hours after catheter insertion;
3. During the corresponding ICU stay; and
4. While at least one qualifying catheter remained active.

The first arterial PaO_2_ measurement satisfying all four criteria was selected as the exposure value. The interval between catheter insertion and the selected PaO_2_ measurement was calculated in minutes. For an additional post hoc descriptive analysis, the interval from ICU admission to the selected PaO_2_ measurement was also calculated in minutes.

PaO_2_ was analyzed in two prespecified forms:

1. As a continuous variable, with odds ratios reported per 10-mmHg increase; and
2. As a categorical variable divided into ≤80 mmHg, 81–100 mmHg, and >100 mmHg, with ≤80 mmHg used as the reference group.

In a secondary sensitivity analysis, the ratio of PaO_2_ to the fraction of inspired oxygen was analyzed as an alternative oxygenation exposure.

### Outcome definitions

The primary outcome was documented acute upper-body deep or central venous thrombosis during the hospital admission. The outcome was identified using a prespecified list of International Classification of Diseases, Ninth and Tenth Revision diagnosis codes recorded in the MIMIC-IV diagnoses_icd table.

The primary outcome definition included acute thrombosis involving deep veins of the upper extremity, axillary veins, subclavian veins, or internal jugular veins. Superficial venous thrombosis and thrombosis identified only by anatomically unspecified upper-extremity codes were not included in the primary outcome.

Two additional outcome definitions were evaluated in sensitivity analyses:

1. A broader secondary outcome that included other documented upper-body venous thrombosis diagnoses, including superficial or less anatomically specific venous thrombosis; and
2. A strict outcome restricted to primary-outcome diagnoses with laterality potentially compatible with the qualifying catheter: a right-sided diagnosis with a right-sided catheter, a left-sided diagnosis with a left-sided catheter, or a bilateral diagnosis. Diagnoses with unspecified laterality did not satisfy the strict definition.

The complete ICD-9-CM and ICD-10-CM code list and the rules used to construct the primary, broader, and strict outcome definitions are provided in Supplementary Table S10 in S1 File. No chart-adjudicated or imaging-adjudicated reference standard was available; therefore, the sensitivity and specificity of the coding algorithm could not be estimated.

Because the diagnosis codes were assigned at the hospitalization level and lacked reliable timestamps, the exact onset time of thrombosis could not be determined. Accordingly, it could not be confirmed that the selected PaO_2_ measurement preceded thrombus formation in every admission. The available data also could not establish that the coded thrombosis was caused by the qualifying catheter. Therefore, the outcome was described as a documented hospitalization-level thrombotic outcome rather than confirmed catheter-related thrombosis.

### Covariates

We collected demographic characteristics, comorbidity burden, laboratory measurements, catheter-related characteristics, measurement timing, respiratory variables, and indicators of illness severity as potential confounding factors. These variables included age at hospital admission, sex, race or ethnicity, Charlson Comorbidity Index, hemoglobin, platelet count, baseline lactate, catheter type, catheter laterality, the number of active catheters, the interval between catheter insertion and PaO_2_ measurement, FiO_2_ at the time of PaO_2_ measurement, respiratory-support status, and the non-respiratory component of the first-day SOFA score. Race or ethnicity was used in descriptive analyses and an additional sensitivity analysis but was not included in the primary fully adjusted model.

Hemoglobin and platelet count were obtained from the most recent complete blood count recorded within the 24 hours preceding the selected PaO_2_ measurement. Lactate was preferentially obtained from the same arterial blood gas record as PaO_2_; when unavailable in the same record, the most recent lactate measurement within the preceding 24 hours was used. Hemoglobin, platelet count, and lactate were analyzed as continuous variables.

## Statistical analysis

Continuous variables were summarized as medians with interquartile ranges, and categorical variables as frequencies and percentages. Standardized mean differences were used to describe between-group differences.

The association between PaO_2_ and the primary outcome was evaluated using logistic regression. PaO_2_ was analyzed continuously, with odds ratios reported per 10-mmHg increase, and categorically as ≤80, 81–100, and >100 mmHg, with ≤80 mmHg as the reference group.

Four models were constructed. Model 0 was unadjusted. Model 1 included age, sex, and Charlson Comorbidity Index. Model 2 additionally included hemoglobin, platelet count, lactate, catheter type, catheter laterality, multiple active catheters, and the log-transformed interval from catheter insertion to PaO_2_ measurement. Model 3 further included FiO_2_, respiratory-support status, and the non-respiratory component of the first-day SOFA score.

Missing values in the prespecified model covariates were handled using multiple imputation by chained equations, with 20 imputed datasets [17]. Each dataset was analyzed separately using patient-clustered robust standard errors, and estimates were combined using Rubin’s rules.

Sensitivity analyses included complete-case analysis; exclusion of PaO_2_ values greater than 400 mmHg; exclusion of values above the 99th percentile; restriction to each patient’s first eligible admission; restriction to a high-reliability FiO_2_ subset, defined as FiO_2_ obtained from the same blood-gas record or from a ventilator setting recorded within 60 minutes of the PaO_2_ measurement; additional adjustment for race or ethnicity; alternative outcome definitions; and substitution of the PaO_2_/FiO_2_ ratio for PaO_2_. An additional narrow-window sensitivity analysis restricted the cohort to admissions in which the selected PaO_2_ measurement was obtained within 60 minutes after catheter insertion. Models 2 and 3 were refitted in this restricted cohort using the same multiple-imputation framework, patient-clustered HC0 robust standard errors, and Rubin’s-rules pooling as in the primary analysis. Results of this analysis are presented in Supplementary Table S12 in S1 File. Because the strict outcome included only 69 events, Firth penalized logistic regression was used as an additional sensitivity analysis. Restricted cubic spline functions were used to examine potential nonlinear associations between PaO_2_ and the primary outcome [18]. Exploratory interaction analyses were conducted according to measurement-time window, invasive mechanical ventilation status, and PICC status. A post hoc module-specific analysis examined changes in the PaO_2_ estimate after adjustment for different groups of covariates. Model performance and stability were assessed using the apparent area under the receiver operating characteristic curve, Brier score, generalized variance inflation factors, leverage, Cook’s distance, and DFBETA statistics. No adjustment for multiple comparisons was applied to secondary and exploratory analyses; these findings were interpreted as hypothesis-generating. Further details are provided in the Supplementary Methods.

No a priori sample-size calculation was performed because this was a retrospective database study. All hospital admissions meeting the prespecified eligibility criteria were included. All analyses were conducted using R version 4.3.3. Tests were two-sided, and P < 0.05 was considered statistically significant.

## Results

### Study population and baseline characteristics

A total of 11,277 hospital admissions from 10,789 unique patients were included in the analytic cohort. The median age was 67.7 years (interquartile range [IQR], 58.2–76.4 years), 64.0% of admissions involved male patients, and the median Charlson Comorbidity Index was 5 (IQR, 4–7). Based on the first qualifying PaO_2_ measurement obtained within 24 hours after catheter insertion, 1,143 admissions (10.1%) had a PaO_2_ of ≤80 mmHg, 1,348 (12.0%) had a PaO_2_ of 81–100 mmHg, and 8,786 (77.9%) had a PaO_2_ of >100 mmHg. The primary outcome occurred in 32 admissions (2.8%) in the PaO_2_ ≤ 80-mmHg group, 29 admissions (2.2%) in the 81–100-mmHg group, and 134 admissions (1.5%) in the > 100-mmHg group, for a total of 195 events (1.73%). In an additional post hoc descriptive analysis, the median interval from ICU admission to the selected PaO_2_ measurement was 299 minutes (IQR, 176–525) in admissions without the primary outcome and 494 minutes (IQR, 232–1,160) in admissions with the primary outcome. Baseline characteristics according to PaO_2_ category and primary-outcome status are presented in Tables 1 and 2, respectively.

**Table 1:** Baseline characteristics according to PaO_2_ category.

Characteristic	PaO_2_ ≤ 80 mmHg(N = 1,143)	PaO_2_ 81–100 mmHg(N = 1,348)	PaO_2_ > 100 mmHg(N = 8,786)
Age, years	65.7 (55.2, 75.8)	67.4 (57.3, 76.1)	68.0 (58.7, 76.5)
Charlson Comorbidity Index	6.0 (4.0, 8.0)	6.0 (4.0, 8.0)	5.0 (4.0, 7.0)
Hemoglobin, g/dL	10.3 (9.0, 11.8)	10.5 (9.2, 11.9)	10.5 (9.3, 11.7)
Platelet count, × 10^9^/L	186.0 (131.0, 257.0)	177.0 (130.0, 239.0)	156.0 (120.0, 205.0)
Baseline lactate, mmol/L	1.9 (1.3, 3.0)	2.0 (1.3, 3.0)	2.2 (1.6, 3.1)
Nonrespiratory SOFA score	7.0 (4.0, 9.0)	6.0 (4.0, 8.0)	5.0 (3.0, 7.0)
FiO_2_, %	60.0 (50.0, 100.0)	50.0 (50.0, 100.0)	80.0 (50.0, 100.0)
Time from catheter insertion to PaO_2_ measurement, min	111.0 (40.0, 336.5)	99.0 (36.0, 248.0)	38.0 (20.0, 124.0)
PaO_2_, mmHg	70.0 (64.0, 76.0)	91.0 (86.0, 96.0)	190.0 (137.0, 287.0)
Sex			
Male	693 (60.6)	861 (63.9)	5,652 (64.3)
Female	450 (39.4)	487 (36.1)	3,134 (35.7)
Race or ethnicity			
White	741 (64.8)	934 (69.3)	6,213 (70.7)
Black	103 (9.0)	81 (6.0)	534 (6.1)
Hispanic or Latino	35 (3.1)	28 (2.1)	289 (3.3)
Asian	30 (2.6)	21 (1.6)	218 (2.5)
Other	48 (4.2)	66 (4.9)	369 (4.2)
Unknown or not reported	186 (16.3)	218 (16.2)	1,163 (13.2)
Respiratory support at PaO_2_ measurement			
Invasive mechanical ventilation	650 (56.9)	846 (62.8)	5,874 (66.9)
Other recorded respiratory support	229 (20.0)	166 (12.3)	321 (3.7)
No contemporaneous respiratory-support interval identified	264 (23.1)	336 (24.9)	2,591 (29.5)
Catheter type			
PICC	140 (12.2)	104 (7.7)	298 (3.4)
Multi-lumen catheter	590 (51.6)	646 (47.9)	2,764 (31.5)
Large-bore or specialized catheter	167 (14.6)	301 (22.3)	3,568 (40.6)
Other qualifying catheter type	246 (21.5)	297 (22.0)	2,156 (24.5)
Catheter laterality			
Right	871 (76.2)	1,094 (81.2)	7,713 (87.8)
Left	271 (23.7)	251 (18.6)	1,067 (12.1)
Unknown	1 (0.1)	3 (0.2)	6 (0.1)
Number of active qualifying catheters			
One active catheter	937 (82.0)	954 (70.8)	4,647 (52.9)
Multiple active catheters	206 (18.0)	394 (29.2)	4,139 (47.1)
Primary outcome			
Documented event, n (%)	32 (2.8)	29 (2.2)	134 (1.5)

Data are presented as median (interquartile range) for continuous variables and n (%) for categorical variables.

SMD denotes the absolute standardized mean difference. For continuous variables, SMDs were calculated using means and pooled standard deviations; for categorical variables, SMDs were calculated from proportions.

The primary outcome was documented acute upper-body deep or central venous thrombosis during hospitalization. Hospital-level diagnosis codes could not establish that the event was caused by the qualifying catheter or occurred after the selected PaO_2_ measurement.

Abbreviations: FiO_2_, fraction of inspired oxygen; IQR, interquartile range; PaO_2_, arterial partial pressure of oxygen; PICC, peripherally inserted central catheter; SMD, standardized mean difference; SOFA, Sequential Organ Failure Assessment.

**Table 2:** Baseline characteristics according to primary-outcome status.

Characteristic	No documented primary outcome(N = 11,082)	Documented primary outcome(N = 195)	SMD
Age, years	67.7 (58.2, 76.4)	64.4 (55.6, 75.2)	0.164
Charlson Comorbidity Index	5.0 (4.0, 7.0)	6.0 (4.0, 7.0)	0.191
Hemoglobin, g/dL	10.5 (9.3, 11.7)	10.0 (8.8, 11.2)	0.253
Platelet count, × 10^9^/L	159.0 (121.0, 214.0)	167.0 (108.0, 249.5)	0.142
Baseline lactate, mmol/L	2.2 (1.5, 3.1)	2.1 (1.4, 3.9)	0.212
Nonrespiratory SOFA score	5.0 (3.0, 7.0)	8.0 (6.0, 10.0)	0.779
FiO_2_, %	60.0 (50.0, 100.0)	55.0 (50.0, 100.0)	0.316
Time from catheter insertion to PaO_2_ measurement, min	45.0 (22.0, 152.0)	134.0 (60.5, 339.0)	0.422
PaO_2_, mmHg	158.0 (106.0, 255.0)	123.0 (93.5, 195.0)	0.279
Sex			
Male	7,089 (64.0)	117 (60.0)	0.082
Female	3,993 (36.0)	78 (40.0)	0.082
Race or ethnicity			
White	7,771 (70.1)	117 (60.0)	0.212
Black	693 (6.3)	25 (12.8)	0.224
Hispanic or Latino	346 (3.1)	6 (3.1)	0.003
Asian	264 (2.4)	5 (2.6)	0.012
Other	471 (4.3)	12 (6.2)	0.086
Unknown or not reported	1,537 (13.9)	30 (15.4)	0.043
Respiratory support at PaO_2_ measurement			
Invasive mechanical ventilation	7,232 (65.3)	138 (70.8)	0.118
Other recorded respiratory support	701 (6.3)	15 (7.7)	0.054
No contemporaneous respiratory-support interval identified	3,149 (28.4)	42 (21.5)	0.159
Catheter type			
PICC	505 (4.6)	37 (19.0)	0.447
Multi-lumen catheter	3,913 (35.3)	87 (44.6)	0.190
Large-bore or specialized catheter	4,013 (36.2)	23 (11.8)	0.572
Other qualifying catheter type	2,651 (23.9)	48 (24.6)	0.016
Catheter laterality			
Right	9,541 (86.1)	137 (70.3)	0.383
Left	1,532 (13.8)	57 (29.2)	0.375
Unknown	9 (0.1)	1 (0.5)	0.079
Number of active qualifying catheters			
One active catheter	6,390 (57.7)	148 (75.9)	0.387
Multiple active catheters	4,692 (42.3)	47 (24.1)	0.387
PaO_2_ category			
≤80 mmHg	1,111 (10.0)	32 (16.4)	0.189
81–100 mmHg	1,319 (11.9)	29 (14.9)	0.087
>100 mmHg	8,652 (78.1)	134 (68.7)	0.212
Missing data, n (%)			
Age	0 (0.0)	0 (0.0)	
Charlson Comorbidity Index	0 (0.0)	0 (0.0)	
Hemoglobin	425 (3.8)	8 (4.1)	
Platelet count	390 (3.5)	4 (2.1)	
Baseline lactate	584 (5.3)	15 (7.7)	
Nonrespiratory SOFA score	892 (8.0)	23 (11.8)	
FiO_2_	1,181 (10.7)	29 (14.9)	
Time from catheter insertion to PaO_2_ measurement	0 (0.0)	0 (0.0)	
PaO_2_	0 (0.0)	0 (0.0)	

Data are presented as median (interquartile range) for continuous variables and n (%) for categorical variables.

SMD denotes the absolute standardized mean difference. For continuous variables, SMDs were calculated using means and pooled standard deviations; for categorical variables, SMDs were calculated from proportions.

The primary outcome was documented acute upper-body deep or central venous thrombosis during hospitalization. Hospital-level diagnosis codes could not establish that the event was caused by the qualifying catheter or occurred after the selected PaO_2_ measurement.

Abbreviations: FiO_2_, fraction of inspired oxygen; IQR, interquartile range; PaO_2_, arterial partial pressure of oxygen; PICC, peripherally inserted central catheter; SMD, standardized mean difference; SOFA, Sequential Organ Failure Assessment.

### Association between PaO_2_ and the primary outcome

When modeled as a continuous variable, higher PaO_2_ was associated with lower odds of the primary outcome in the unadjusted model, with an OR of 0.970 for each 10-mmHg increase in PaO_2_ (95% CI, 0.952–0.987; P < 0.001). The association remained statistically significant after adjustment for age, sex, and Charlson Comorbidity Index (Model 1: OR, 0.975; 95% CI, 0.957–0.993; P = 0.006). After additional adjustment for laboratory measurements, catheter-related characteristics, and the interval between catheter insertion and PaO_2_ measurement, the association was substantially attenuated and was no longer statistically significant (Model 2: OR, 1.003; 95% CI, 0.984–1.022; P = 0.77). No association was observed in the fully adjusted model, which additionally included FiO_2_, respiratory-support status, and the non-respiratory SOFA score (Model 3: OR, 1.008; 95% CI, 0.989–1.027; P = 0.43; Table 3).

**Table 3:** Association between continuous PaO_2_ and the documented thrombotic outcome.

Model	Exposure	OR (95% CI)	P value	N	Events
Model 0	PaO_2_ per 10-mmHg increase	0.970 (0.952–0.987)	<0.001	11,277	195
Model 1	PaO_2_ per 10-mmHg increase	0.975 (0.957–0.993)	0.006	11,277	195
Model 2	PaO_2_ per 10-mmHg increase	1.003 (0.984–1.022)	0.774	11,277	195
Model 3	PaO_2_ per 10-mmHg increase	1.008 (0.989–1.027)	0.430	11,277	195

The outcome was documented acute upper-body deep or central venous thrombosis during hospitalization.

Model 0: unadjusted. *Model 1:* adjusted for age, sex, and Charlson Comorbidity Index. *Model 2:* additionally adjusted for hemoglobin, platelet count, baseline lactate, catheter type, catheter laterality, multiple active qualifying catheters, and ln(minutes from catheter insertion to PaO_2_ measurement + 1). *Model 3:* additionally adjusted for FiO_2_ at the time of PaO_2_ measurement, respiratory-support status, and non-respiratory first-day SOFA score.

Statistical analysis: Missing values in prespecified covariates were handled using multiple imputation by chained equations (20 datasets). Logistic regression models used patient-clustered HC0 robust standard errors, and estimates were combined using Rubin’s rules. All P values are two-sided.

Abbreviations: CI, confidence interval; FiO_2_, fraction of inspired oxygen; OR, odds ratio; PaO_2_, arterial partial pressure of oxygen; SOFA, Sequential Organ Failure Assessment.

In the categorical analysis, compared with admissions with a PaO_2_ of ≤80 mmHg, those with a PaO_2_ of >100 mmHg had lower odds of the primary outcome in the unadjusted model (OR, 0.538; 95% CI, 0.364–0.795; P = 0.002) and in Model 1 (OR, 0.603; 95% CI, 0.405–0.899; P = 0.013). However, this association was attenuated after further adjustment and was not statistically significant in Model 2 (OR, 0.961; 95% CI, 0.634–1.456; P = 0.85) or Model 3 (OR, 0.989; 95% CI, 0.652–1.502; P = 0.96). No statistically significant association was observed for the 81–100-mmHg group compared with the ≤ 80-mmHg group in any model (Table 4).

**Table 4:** Association between PaO_2_ categories and the documented thrombotic outcome.

Model	PaO_2_ category comparison	OR (95% CI)	P value	N	Events
Model 0	81–100 vs ≤ 80 mmHg	0.763 (0.455–1.281)	0.307	11,277	195
	>100 vs ≤ 80 mmHg	0.538 (0.364–0.795)	0.002	11,277	195
Model 1	81–100 vs ≤ 80 mmHg	0.802 (0.477–1.349)	0.406	11,277	195
	>100 vs ≤ 80 mmHg	0.603 (0.405–0.899)	0.013	11,277	195
Model 2	81–100 vs ≤ 80 mmHg	0.927 (0.549–1.565)	0.776	11,277	195
	>100 vs ≤ 80 mmHg	0.961 (0.634–1.456)	0.850	11,277	195
Model 3	81–100 vs ≤ 80 mmHg	0.900 (0.531–1.526)	0.695	11,277	195
	>100 vs ≤ 80 mmHg	0.989 (0.652–1.502)	0.960	11,277	195

*Note:* The PaO_2_ ≤ 80-mmHg category was the reference group. The outcome was documented acute upper-body deep or central venous thrombosis during hospitalization.

*Model 0*: unadjusted. *Model 1:* adjusted for age, sex, and Charlson Comorbidity Index. *Model 2:* additionally adjusted for hemoglobin, platelet count, baseline lactate, catheter type, catheter laterality, multiple active qualifying catheters, and ln(minutes from catheter insertion to PaO_2_ measurement + 1). *Model 3:* additionally adjusted for FiO_2_ at the time of PaO_2_ measurement, respiratory-support status, and non-respiratory first-day SOFA score.

*Statistical analysis*: Missing values in prespecified covariates were handled using multiple imputation by chained equations (20 datasets). Logistic regression models used patient-clustered HC0 robust standard errors, and estimates were combined using Rubin’s rules. All P values are two-sided.

*Abbreviations*: CI, confidence interval; FiO_2_, fraction of inspired oxygen; OR, odds ratio; PaO_2_, arterial partial pressure of oxygen; SOFA, Sequential Organ Failure Assessment.

### Sensitivity, nonlinear, and subgroup analyses

Most prespecified sensitivity analyses were broadly consistent with the primary complete-cohort result. After exclusion of the non-respiratory SOFA score, restriction to complete cases, exclusion of extreme PaO_2_ values, restriction to the first eligible admission for each patient, restriction to admissions with high-reliability FiO_2_ measurements, and additional adjustment for race or ethnicity, the adjusted ORs for the primary outcome per 10-mmHg increase in PaO_2_ ranged from 1.001 to 1.017, with none reaching statistical significance. Analyses using PaO_2_ categories yielded similar results. Detailed estimates from the continuous and categorical sensitivity analyses are presented in Supplementary Tables S1 and S2 in S1 File, respectively. In an additional post hoc descriptive summary of the upper tail of the PaO_2_ distribution, 647 admissions with 10 outcome events had PaO_2_ values greater than 384 mmHg, 490 admissions with 5 events had values greater than 400 mmHg, and 111 admissions with 2 events had values above the 99th percentile (>461 mmHg). These data are presented in Supplementary Table S11 in S1 File.

The findings were also unchanged when alternative outcome and exposure definitions were applied. For the broader secondary outcome, which occurred in 282 admissions, the adjusted OR per 10-mmHg increase in PaO_2_ was 1.002 (95% CI, 0.986–1.018; P = 0.81). For the strict outcome definition, which occurred in 69 admissions, the corresponding OR was 1.008 (95% CI, 0.974–1.042; P = 0.66), with a similar estimate obtained using Firth penalized logistic regression. The PaO_2_/FiO_2_ ratio was likewise not associated with the primary outcome (OR per 50-unit increase, 1.010; 95% CI, 0.957–1.065; P = 0.73). Detailed results of the alternative outcome and exposure analyses are provided in Supplementary Table S3 in S1 File.

In an additional narrow-window sensitivity analysis restricted to admissions with PaO_2_ measured within 60 minutes after catheter insertion, 6,301 admissions from 6,185 patients were included, with 49 primary outcome events. The association was not statistically significant in Model 2 (OR per 10-mmHg increase, 1.026; 95% CI, 0.995–1.058; P = 0.102), whereas fully adjusted Model 3 showed a positive association (OR, 1.041; 95% CI, 1.010–1.073; P = 0.010; Supplementary Table S12 in S1 File).

Restricted cubic spline analysis showed no evidence of an overall or nonlinear association between PaO_2_ and the primary outcome (P for overall association = 0.186; P for nonlinearity = 0.109; Fig 2).

**Fig 2:**
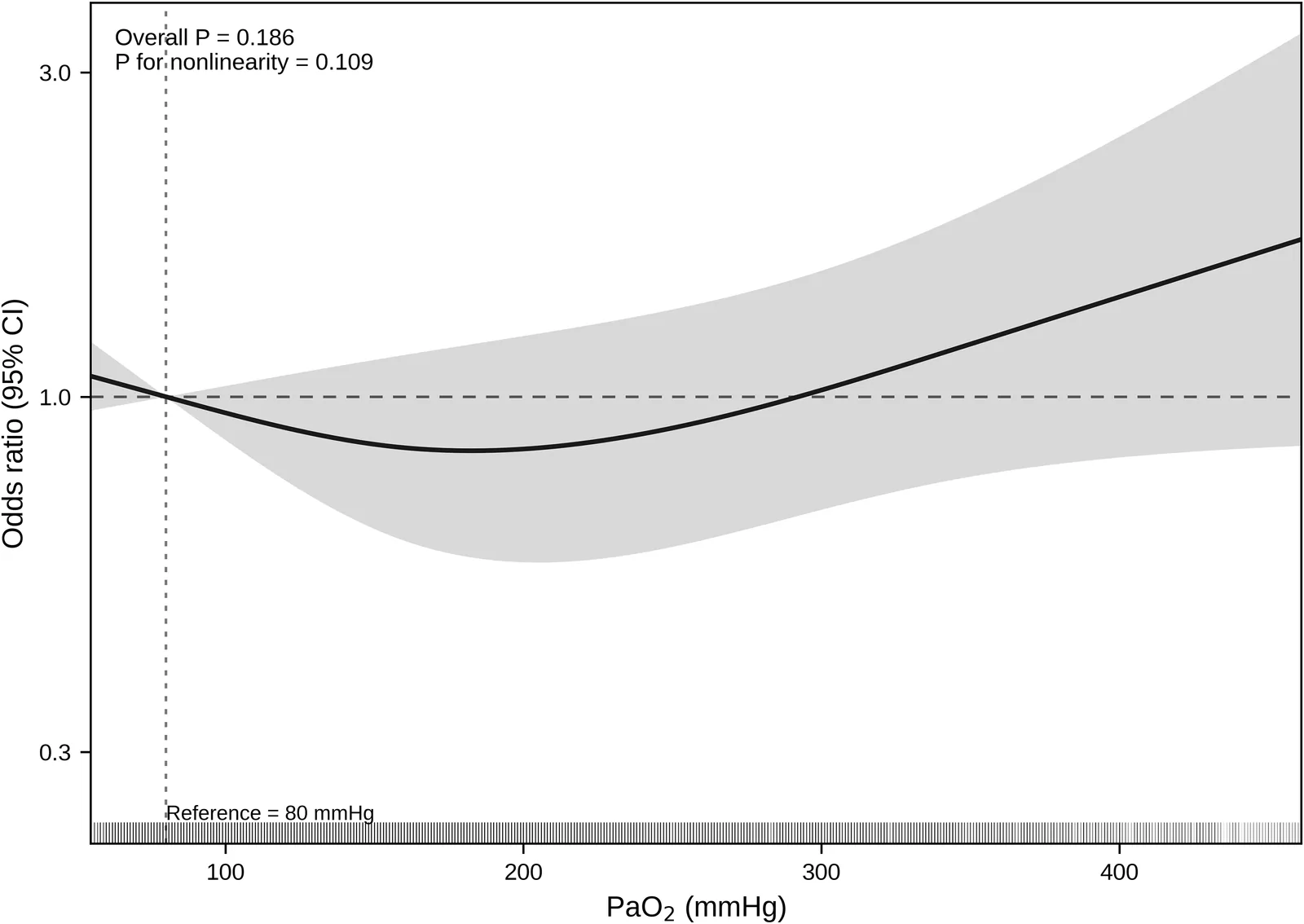
Restricted cubic spline analysis of the association between arterial PaO_2_ and the documented thrombotic outcome.

There was no evidence of effect modification according to the interval from catheter insertion to PaO_2_ measurement (P for interaction = 0.223), invasive mechanical ventilation status (P for interaction = 0.522), or PICC status (P for interaction = 0.453). Subgroup-specific estimates were not statistically significant (Supplementary Table S4 and Supplementary Figure S1 in S1 File).

The mean apparent area under the receiver operating characteristic curve across the 20 imputed datasets was 0.7834, and the mean apparent Brier score was 0.0166. No substantial multicollinearity was identified (maximum adjusted generalized variance inflation factor, 1.318). The maximum PaO_2_-specific absolute DFBETA was 0.390. Excluding two admissions identified by the overall DFBETA assessment did not materially change the PaO_2_ estimate (OR per 10-mmHg increase, 1.006; 95% CI, 0.987–1.025; P = 0.555). Additional apparent discrimination, calibration, multicollinearity, and influence diagnostics are provided in Supplementary Table S5 and Supplementary Figure S2 in S1 File.

The analysis included 11,277 hospital admissions and 195 outcome events. The solid line represents the adjusted odds ratio, and the shaded area represents the 95% confidence interval. Three knots were placed at the 10th, 50th, and 90th percentiles of the PaO_2_ distribution (80, 158, and 346 mmHg, respectively), with 80 mmHg as the reference value. Rug marks indicate the distribution of observed PaO_2_ values. The model was adjusted for the covariates included in Model 3. Estimates were combined across 20 multiply imputed datasets using Rubin’s rules, with patient-clustered robust standard errors. Overall association, P = 0.186; nonlinearity, P = 0.109.

### Secondary associations of model covariates with the documented thrombotic outcome

In an exploratory examination of the fully adjusted model, several covariates were associated with the documented thrombotic outcome. Compared with multi-lumen catheters, PICC use was associated with higher odds of the outcome (OR, 2.71; 95% CI, 1.77–4.17; P < 0.001), whereas large-bore or specialized catheters were associated with lower odds (OR, 0.44; 95% CI, 0.24–0.80; P = 0.007). Left-sided catheter placement was associated with higher odds than right-sided placement (OR, 1.43; 95% CI, 1.01–2.02; P = 0.044). A longer interval between catheter insertion and PaO_2_ measurement was also associated with higher odds of the outcome (OR, 1.33 per one-unit increase in ln[minutes + 1]; 95% CI, 1.15–1.53; P < 0.001), as was a higher non-respiratory SOFA score (OR, 1.15 per point; 95% CI, 1.09–1.20; P < 0.001). These findings were considered secondary associations and were not interpreted as causal effects. Complete estimates for all covariates included in the fully adjusted model are provided in Supplementary Table S8 in S1 File.

### Post hoc module-specific analysis

In the post hoc module-specific analysis, the inverse association observed in Model 1 (OR per 10-mmHg increase in PaO_2_, 0.975; 95% CI, 0.957–0.993; P = 0.006) was most substantially attenuated after the addition of catheter-related characteristics and the interval from catheter insertion to PaO_2_ measurement (OR, 1.004; 95% CI, 0.985–1.023; P = 0.687). Adding the laboratory module alone resulted in minimal change in the effect estimate (OR, 0.974; 95% CI, 0.956–0.992; P = 0.006), whereas addition of the oxygenation and illness-severity module shifted the estimate toward the null (OR, 0.992; 95% CI, 0.973–1.011; P = 0.388). After simultaneous adjustment for all covariate modules in Model 3, the association was no longer statistically significant (OR, 1.008; 95% CI, 0.989–1.027; P = 0.430; Fig 3 and Supplementary Table S7 in S1 File). These analyses were exploratory and descriptive; they were not intended to determine whether any individual covariate was responsible for the observed change in the PaO_2_ estimate.

**Fig 3:**
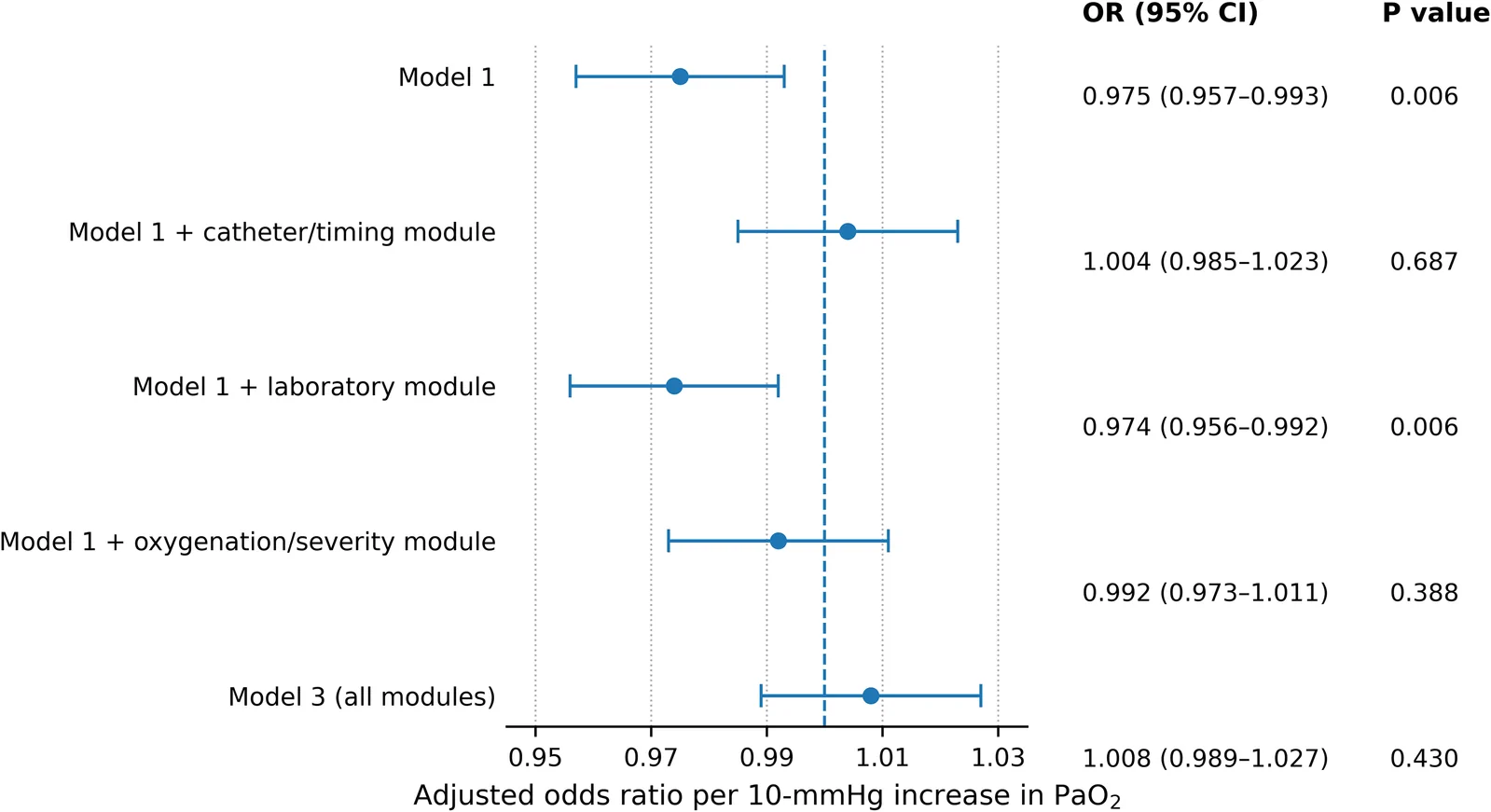
Changes in the estimated association between arterial PaO_2_ and the documented thrombotic outcome after adjustment for different covariate modules.

Odds ratios are reported for each 10-mmHg increase in PaO_2_. Model 1 was adjusted for age, sex, and Charlson Comorbidity Index. Each module-specific model added the indicated module separately to Model 1. The catheter/timing module included catheter type, catheter laterality, multiple active qualifying catheters, and ln(minutes from catheter insertion to PaO_2_ measurement + 1). The laboratory module included hemoglobin, platelet count, and baseline lactate. The oxygenation/severity module included FiO_2_ at the time of PaO_2_ measurement, respiratory-support status, and the non-respiratory first-day SOFA score. Model 3 included all covariates. Estimates were combined across 20 multiply imputed datasets using Rubin’s rules, with patient-clustered robust standard errors. The module-specific analyses were exploratory and descriptive.

## Discussion

In this retrospective observational cohort study of 11,277 hospital admissions involving critically ill adults with qualifying invasive venous catheter episodes, arterial PaO_2_ measured within 24 hours after catheter insertion was not associated with documented acute upper-body deep or central venous thrombosis after comprehensive adjustment for patient characteristics, laboratory measurements, catheter-related factors, measurement timing, FiO_2_, respiratory-support status, and illness severity. Higher PaO_2_ was associated with lower odds of the outcome in the unadjusted and minimally adjusted models; however, the estimate moved toward the null after catheter-related characteristics and the interval from catheter insertion to PaO_2_ measurement were incorporated into the model. Most secondary and sensitivity analyses in the complete cohort were broadly consistent with the primary null result, although the narrow-window analysis yielded adjustment-set-dependent estimates.

These findings should not be interpreted as evidence that arterial oxygenation has no biological relationship with thrombosis under all circumstances. Rather, they indicate that a single early PaO_2_ measurement obtained during routine ICU care was not independently associated with the documented thrombotic outcome after the major clinical and catheter-related covariates available in the database were considered. The attenuation of the initial association highlights the importance of accounting for the clinical context in which PaO_2_ is measured, particularly among critically ill patients receiving invasive vascular access and respiratory support.

A single arterial PaO_2_ value obtained during routine ICU care should not be regarded as an isolated physiological exposure. It is jointly influenced by pulmonary gas exchange, the administered FiO_2_, respiratory-support settings, and the patient’s evolving clinical condition. The role of FiO_2_ adjustment depends on the estimand of interest because FiO_2_ is a direct determinant of the measured PaO_2_. Importantly, however, the association was already null in Model 2, before further adjustment for FiO_2_, respiratory-support status, and non-respiratory SOFA score (OR per 10-mmHg increase, 1.003; 95% CI, 0.984–1.022; P = 0.774). Thus, the null primary finding cannot be attributed solely to adjustment for FiO_2_. The PaO_2_/FiO_2_ ratio was therefore retained as an alternative exposure in sensitivity analysis rather than included as an additional covariate in the primary models. Systematic reviews of oxygenation targets in mechanically ventilated and general ICU populations have demonstrated substantial heterogeneity in oxygen targets and uncertainty regarding the optimal level of arterial oxygenation, further illustrating that an individual PaO_2_ value cannot be interpreted independently of oxygen delivery and respiratory management [12,19].

The additional analysis restricted to PaO_2_ measurements obtained within 60 minutes after catheter insertion yielded more heterogeneous estimates. The association was not statistically significant in Model 2 but became positive in the fully adjusted Model 3. This finding should be interpreted cautiously because only 49 outcome events remained in the restricted cohort, and the estimate was sensitive to the adjustment set. In addition, restricting the analysis to patients with very early post-insertion blood-gas measurements may have introduced selection related to clinical severity and measurement timing. Accordingly, this result is best viewed as a secondary, hypothesis-generating finding rather than evidence that overrides the primary complete-cohort analysis.

At the same time, venous thromboembolism in critically ill patients is associated with multiple patient- and treatment-related factors. Previous systematic reviews have identified central venous catheterization, invasive mechanical ventilation, sepsis, vasoactive therapy, malignancy, previous venous thromboembolism, and prolonged ICU treatment as relevant prognostic factors [5,20]. Accordingly, the inverse association observed in the less extensively adjusted models may have reflected differences in clinical condition and treatment context rather than an independent association between PaO_2_ and the documented thrombotic outcome.

The post hoc module-specific analysis was consistent with this interpretation. The largest change in the PaO_2_ estimate occurred after addition of the catheter and measurement-timing module, whereas adjustment for laboratory variables alone produced little change. Previous evidence indicates that PICC-associated thrombosis may vary with device- and insertion-related characteristics and has been associated with venous blood-flow velocity, insertion site, and time since catheter placement [21]. However, the present analysis cannot establish that any single catheter variable accounted for the attenuation, nor can it determine whether the documented thrombotic events were caused by the qualifying catheter. The module-specific findings should therefore be interpreted as descriptive evidence that the estimated association between PaO_2_ and the outcome was sensitive to the broader catheter-related and temporal context, rather than as proof of a specific causal pathway.

Biological plausibility exists for an association between abnormal oxygenation and thrombosis, although the pattern and duration of oxygen exposure are likely to be important. In obstructive sleep apnea, recurrent hypoxia–reoxygenation has been proposed to promote oxidative stress and inflammatory signaling, increase the expression of procoagulant mediators such as plasminogen activator inhibitor-1 and tissue factor, alter blood viscosity, and impair endothelial function. Collectively, these changes may affect hypercoagulability, venous stasis, and endothelial injury, the three components of Virchow’s triad [7]. However, these mechanisms arise in the context of chronic, recurrent intermittent hypoxia and cannot be directly extrapolated to a single arterial PaO_2_ measurement obtained during routine ICU care.

Evidence regarding the effects of hypoxia on hemostasis is also inconsistent. A systematic review of environmental hypoxia in healthy adults found only small and heterogeneous changes in coagulation at high altitude. Increased thrombin generation appeared to be accompanied by reduced platelet activation, fibrinolysis was largely unchanged, and available viscoelastic measurements did not consistently indicate increased thrombogenicity. The review therefore could not establish environmental hypoxia as an independent cause of hypercoagulability, particularly because exercise, hydration status, stress, and other concurrent exposures could not be fully separated from the effects of hypoxia [8]. These observations illustrate that the relationship between oxygen tension and hemostasis is context dependent and may be substantially influenced by concurrent physiological and clinical factors.

Some disease-specific clinical studies have reported lower PaO_2_ among patients with venous thromboembolism. A meta-analysis of patients with COPD found a small pooled difference in PaO_2_ between those with and without venous thromboembolism, while also identifying prolonged immobility, invasive mechanical ventilation, neoplasia, older age, and previous venous thrombosis as associated factors [9]. Similarly, a meta-analysis of patients with acute exacerbations of COPD found that those with pulmonary embolism had lower PaO_2_ than those without pulmonary embolism [10]. These findings should be interpreted within the broader pathophysiological context of COPD, in which mitochondrial injury and the release of mitochondrial damage-associated molecular patterns may amplify inflammatory signaling [22]. Thus, lower PaO_2_ in these populations may coexist with inflammation, respiratory failure, reduced mobility, mechanical ventilation, and other factors related to thrombotic risk rather than representing an isolated exposure.

These disease-specific findings are not directly comparable with the present study. Previous studies mainly evaluated pulmonary embolism or conventional venous thromboembolism in COPD populations and frequently assessed oxygenation during the same clinical episode in which thromboembolism was diagnosed. Because pulmonary embolism itself may impair gas exchange and reduce PaO_2_, reverse causation cannot be excluded [10]. By contrast, our exposure was defined using a PaO_2_ measurement obtained within 24 hours after catheter insertion, and the outcome was documented upper-body deep or central venous thrombosis. Nevertheless, because the exact onset time of thrombosis was unavailable, some temporal uncertainty also remains in our study.

Potential adverse effects are also biologically plausible at the higher end of oxygen exposure. Hyperoxia may increase reactive oxygen species production, inflammation, and endothelial injury. A systematic review of randomized perioperative studies found that higher FiO_2_ was associated with increases in several oxidative-stress biomarkers and reductions in some antioxidant markers, although the evidence was derived from a small number of predominantly single-center surgical studies [11]. In mechanically ventilated ICU patients, trials comparing higher and lower oxygenation targets have used heterogeneous and sometimes overlapping target ranges, and the optimal oxygenation strategy remains uncertain [12]. Neither body of evidence directly evaluated upper-body venous thrombosis, and an administered FiO_2_ strategy or cumulative hyperoxic exposure is not equivalent to a single measured PaO_2_ value. Therefore, our results should not be interpreted as evidence that higher PaO_2_ protects against thrombosis or that oxygen administration should be increased for thrombosis prevention. Future studies should evaluate repeated or time-weighted oxygenation measurements together with accurately timed and imaging-confirmed thrombotic outcomes.

Several catheter- and patient-related variables were associated with the documented thrombotic outcome in the fully adjusted model. PICC use, left-sided catheter placement, a longer interval from catheter insertion to the selected PaO_2_ measurement, and a higher non-respiratory SOFA score were associated with higher odds of the outcome. In contrast, the large-bore or specialized catheter category was associated with lower odds. These findings provide additional insight into the clinical and device-related context in which upper-body venous thrombosis was documented.

The association between PICC use and higher odds of thrombosis is consistent with previous evidence. A systematic review and meta-analysis of 75 studies involving 109,292 hospitalized patients found that PICCs were associated with higher odds of venous thromboembolism than conventional CVCs, with the highest pooled incidence reported in critical care populations [2]. Although the difference was not statistically significant when the analysis was restricted to randomized trials, the overall findings support the view that PICC-associated thrombotic risk may be clinically relevant in selected hospitalized populations. A separate meta-analysis of prospective studies showed that the incidence of symptomatic PICC-related deep vein thrombosis was comparatively low when contemporary insertion practices were used, including ultrasound-guided venipuncture, appropriate catheter-size selection, and verification of catheter-tip position [6]. Taken together, these findings suggest that the thrombotic profile of a PICC is likely determined not only by the device type itself but also by catheter size, the catheter-to-vein relationship, tip position, insertion technique, treatment indication, and patient characteristics. Observational evidence has also suggested that a catheter-to-vein ratio greater than approximately 45% may be associated with a higher occurrence of PICC-related thrombosis, although the optimal threshold remains uncertain [23]. Many of these technical factors were unavailable in MIMIC-IV and may partly account for the association observed in our study.

The higher odds observed with left-sided placement may also have a plausible vascular basis. Previous evidence has linked slower venous blood flow and left basilic-vein placement with asymptomatic PICC-related thrombosis, and most detected events occurred within 3–12 days after insertion [21]. A longer and potentially more angulated venous course on the left side may contribute to altered flow, greater catheter–vessel contact, or local venous stasis. Although the populations and outcome definitions in previous studies differ from ours, the consistency in direction suggests that catheter laterality and local venous anatomy may be relevant when evaluating upper-body thrombotic events.

The association between a higher non-respiratory SOFA score and the thrombotic outcome is consistent with the broader thrombotic vulnerability of critically ill patients. A meta-analysis of 39 ICU cohorts identified central venous catheterization, invasive mechanical ventilation, sepsis, vasoactive medication use, and lack of pharmacologic thromboprophylaxis as factors associated with venous thromboembolism [5]. Higher non-respiratory SOFA scores may therefore reflect a greater burden of organ dysfunction, systemic inflammation, hemodynamic instability, immobility, and invasive treatment. In this context, the SOFA association is clinically plausible as a marker of the overall severity and treatment intensity accompanying thrombotic risk.

The association with a longer interval from catheter insertion to the selected PaO_2_ measurement may reflect the evolving clinical course after catheter placement. Patients whose blood gas measurement occurred later may have had more prolonged or complex ICU management, greater cumulative exposure to invasive devices, or more opportunities for thrombotic events to be investigated and documented. This variable was not equivalent to total catheter dwell time, but its association suggests that the temporal context surrounding catheter placement and subsequent monitoring may be relevant to thrombotic risk assessment.

The lower odds observed for the large-bore or specialized catheter category were unexpected. Central venous access devices used in adult critical care differ considerably in their indications, duration of use, insertion settings, and complication profiles [1]. Large-bore or specialized catheters may be inserted for short-term, procedure-specific indications and removed earlier than standard multi-lumen catheters, or they may be used in patient groups with different surveillance and treatment pathways. The inverse association may therefore reflect differences in device indication and patterns of use rather than an inherently lower thrombogenic potential. Nevertheless, this finding highlights the importance of considering catheter categories separately rather than treating all central venous access devices as equivalent.

Overall, these secondary findings suggest that catheter type, laterality, temporal context, and illness severity may be more informative correlates of the documented thrombotic outcome than a single early PaO_2_ measurement. Because the analyses were observational and not designed to establish causal effects for individual covariates, these associations should be interpreted as hypothesis-generating and warrant confirmation in datasets containing detailed device characteristics and accurately timed thrombotic events.

Our findings have several practical implications. First, a single arterial PaO_2_ measurement obtained within 24 hours after catheter insertion should not be used in isolation to stratify the risk of documented upper-body deep or central venous thrombosis. The attenuation of the association after comprehensive adjustment, together with the broadly consistent findings from most sensitivity, nonlinear, and subgroup analyses, does not support modifying oxygen administration solely to reduce thrombotic risk. Oxygen therapy should continue to be guided by established acute-care oxygenation goals and by the patient’s respiratory, hemodynamic, and overall clinical needs [24].

Second, thrombotic risk assessment in patients with invasive venous catheters should place greater emphasis on the clinical and device-related context, including catheter type, the indication for PICC placement, insertion laterality, catheter size and tip position when available, ongoing device necessity, and the patient’s overall severity of illness(1, 2, 5, 6). Existing clinical guidance on PICC-related thrombosis prevention is heterogeneous, and a substantial proportion of recommendations are supported by low-certainty evidence or expert consensus [25]. These considerations favor individualized catheter selection, careful insertion and maintenance practices, and clinical monitoring rather than the use of a PaO_2_ threshold as a basis for thrombosis prevention. Because the exact onset of thrombosis and standardized imaging ascertainment were unavailable, our findings do not provide a basis for introducing new PaO_2_-directed oxygen targets, routine ultrasound screening, or anticoagulant prophylaxis strategies.

This study has several strengths. It included a large cohort of 11,277 hospital admissions involving 10,789 critically ill patients with qualifying invasive venous catheter episodes. The exposure definition incorporated a clinically relevant temporal framework by requiring the arterial PaO_2_ measurement to occur within 24 hours after catheter insertion, during the ICU stay, and while the qualifying catheter remained in situ. The analyses accounted for a broad range of potential confounders, including demographic and comorbidity characteristics, laboratory measurements, catheter type and laterality, multiple active catheters, the interval from catheter insertion to PaO_2_ measurement, FiO_2_, respiratory-support status, and non-respiratory organ dysfunction. Patient-clustered robust standard errors were used to account for repeated admissions, and multiple imputation reduced the loss of information associated with missing covariate data. The broadly consistent findings across most continuous, categorical, nonlinear, alternative-outcome, alternative-exposure, subgroup, and sensitivity analyses support the overall stability of the primary complete-cohort result.

Several limitations should also be acknowledged. First, the retrospective observational design precludes causal inference and leaves the possibility of residual or unmeasured confounding despite extensive adjustment. More importantly, the database did not provide a sufficiently precise time of thrombus onset. Consequently, it was not possible to confirm that the selected PaO_2_ measurement preceded thrombus formation in every admission. It was therefore also not possible to calculate the interval from PaO_2_ measurement to thrombus onset or to perform a valid time-to-event or competing-risk analysis. The outcome was identified from hospitalization-level diagnostic records rather than through a standardized prospective imaging protocol. No chart-adjudicated or imaging-adjudicated reference standard was available, and the sensitivity and specificity of the coding algorithm are therefore unknown. As a result, asymptomatic or clinically unsuspected thromboses may have been missed, and the observed event rate may therefore be lower than rates reported in studies using systematic imaging surveillance. Outcome ascertainment may also have been influenced by diagnostic testing and documentation practices, and the recorded events could not be definitively attributed to the qualifying catheter. The outcome should therefore be interpreted as documented acute upper-body deep or central venous thrombosis during hospitalization rather than confirmed catheter-related thrombosis.

Second, the exposure was based on a single early PaO_2_ measurement. This value may not adequately represent the duration, variability, or cumulative burden of hypoxemia or hyperoxemia over the course of critical illness or catheter exposure. PaO_2_ is also influenced by FiO_2_, ventilatory management, pulmonary gas exchange, perfusion, and the timing of arterial blood-gas sampling. Although these factors were addressed where possible, only patients with an eligible arterial blood-gas measurement were included, which may have introduced selection bias. In addition, the timing of blood-gas measurement was clinically determined rather than protocolized, and restricting analyses to very early measurements may therefore select patients with different illness severity, monitoring intensity, or clinical trajectories. In addition, several potentially relevant catheter characteristics were unavailable or could not be measured reliably, including catheter material, exact diameter, catheter-to-vein ratio, precise tip location, insertion technique, operator-related factors, and complete catheter dwell time. Although catheter insertion and removal records were used to determine whether a qualifying catheter was active at the time of PaO_2_ measurement, the catheter dwell time before thrombus onset could not be calculated because reliable thrombus-onset timestamps were unavailable. Information on imaging surveillance and time-varying thromboprophylaxis or anticoagulant treatment was also limited, leaving the possibility of residual confounding and differential outcome detection.

Finally, MIMIC-IV reflects practice at a single academic medical center between 2008 and 2019. Changes in oxygen therapy, vascular-access practice, thromboprophylaxis, and diagnostic imaging over time may limit the applicability of the findings to other institutions and contemporary practice. The primary outcome occurred in 195 admissions, the strict outcome occurred in only 69 admissions, and the 60-minute restricted cohort included only 49 primary outcome events; therefore, some subgroup and sensitivity analyses may have had limited statistical precision and increased susceptibility to model instability. The statistically significant associations observed for several model covariates should also be regarded as secondary, hypothesis-generating findings, particularly because multiple exploratory comparisons were performed.

## Conclusion

In the complete cohort of critically ill patients with invasive venous catheters, the first arterial PaO_2_ measured within 24 hours after catheter insertion was not associated with documented acute upper-body deep or central venous thrombosis after comprehensive adjustment. The apparent inverse association observed in less-adjusted models was largely attenuated after accounting for catheter characteristics, measurement timing, respiratory support, and illness severity, although the additional 60-minute sensitivity analysis yielded adjustment-dependent estimates. In secondary analyses, catheter type, placement laterality, measurement timing, and non-respiratory organ dysfunction were associated with the documented thrombotic outcome, although these exploratory findings require confirmation. Future studies incorporating repeated oxygenation measurements, detailed catheter characteristics, standardized imaging assessment, and accurately timed thrombotic events are needed to clarify whether dynamic oxygen exposure contributes to upper-body venous thrombosis.

## Supporting information

S1 File Supplementary Information. Supplementary methods, Tables S1–S12, and Figures S1–S4.
(DOCX)
