# Supplementary material for: Association between early post-insertion arterial oxygen tension and documented acute upper-body deep or central venous thrombosis in critically ill adults: A retrospective MIMIC-IV cohort study
Source: PLoS One. 2026 Oct 5;21(10):e0359719. doi: 10.1371/journal.pone.0359719 (PMC13637912; doi:10.1371/journal.pone.0359719)
Supplement: Supplementary file 1 [file pone.0359719.s001.docx]

**Supplementary Information**

**Methods, Tables, and Figures**

Supplementary Methods

Supplementary Table S1. Sensitivity analyses for continuous PaO₂

Supplementary Table S2. Sensitivity analyses for categorical PaO₂

Supplementary Table S3. Alternative outcome and exposure analyses

Supplementary Table S4. Subgroup analyses

Supplementary Table S5. Model diagnostics and influence analysis

Supplementary Table S6. Missing data and multiple-imputation summary

Supplementary Table S7. Post hoc module-specific analysis

Supplementary Table S8. Fully adjusted Model 3 coefficients

Supplementary Table S9. Pairwise standardized mean differences across PaO₂ categories

Supplementary Table S10. ICD-9-CM and ICD-10-CM codes used to define the outcomes

Supplementary Table S11. Timing characteristics and upper-tail distribution of PaO₂

Supplementary Table S12. Sensitivity analysis restricted to PaO₂ measurements obtained within 60 minutes after catheter insertion

Supplementary Figure S1. Subgroup forest plot

Supplementary Figure S2. Apparent calibration plot

Supplementary Figure S3. Multiple-imputation trace plots

Supplementary Figure S4. Multiple-imputation distributional diagnostics

**Supplementary Methods**

**Cohort construction and exposure alignment**

The hospital admission was the primary unit of analysis because thrombotic diagnosis codes were available at the hospitalization level. When more than one ICU stay within the same hospital admission met the eligibility criteria, only the earliest eligible ICU stay was retained. A patient could contribute more than one eligible hospital admission; patient-clustered robust standard errors were therefore used in the regression analyses.

Qualifying catheter episodes were reconstructed from documented insertion and removal times. The beginning of the qualifying catheter episode was used as the catheter index time. The selected arterial PaO₂ measurement was the first measurement obtained at or after catheter insertion, within 24 hours after insertion, during the corresponding ICU stay, and while at least one qualifying catheter remained active. The interval from catheter insertion to the selected PaO₂ measurement was calculated in minutes and entered into adjusted models as ln(minutes + 1). The interval from ICU admission to the selected PaO₂ measurement was calculated only for an additional post hoc descriptive analysis.

**Outcome classification**

Hospitalization-level ICD-9-CM and ICD-10-CM diagnosis codes were classified using a frozen code dictionary. The primary outcome included documented acute thrombosis of the deep veins of the upper extremity, axillary veins, subclavian veins, or internal jugular veins. The broader secondary outcome included all acute upper-extremity venous thrombosis codes in the dictionary, including superficial and anatomically unspecified diagnoses. The strict sensitivity outcome required the primary outcome definition and laterality potentially compatible with the qualifying catheter: a right-sided diagnosis with a right-sided catheter, a left-sided diagnosis with a left-sided catheter, or a bilateral diagnosis. Diagnoses with unspecified laterality did not satisfy the strict definition. The exact acute codes and classification rules are shown in Supplementary Table S10. Because diagnosis codes lacked reliable onset timestamps, the analyses could not establish that a coded event occurred after the selected PaO₂ measurement or was caused by the qualifying catheter.

**Covariate extraction and missing data**

Hemoglobin and platelet count were taken from the most recent complete blood count during the 24 hours preceding the selected PaO₂ measurement. Lactate was obtained preferentially from the same arterial blood-gas record as PaO₂; otherwise, the most recent lactate value during the preceding 24 hours was used. FiO₂ was matched to the PaO₂ measurement using the available blood-gas and respiratory-support records. The high-reliability FiO₂ sensitivity subset required FiO₂ from the same blood-gas record or from a ventilator setting recorded within 60 minutes of the PaO₂ measurement.

Missing values in prespecified model covariates were handled using multiple imputation by chained equations. Twenty completed datasets were generated using 20 iterations and random seed 20260717. Continuous variables were imputed using predictive mean matching, and the small number of missing catheter-laterality values were imputed using logistic regression. Exposure and outcome variables were not imputed. Missingness and imputation summaries are provided in Supplementary Table S6, with graphical diagnostics in Supplementary Figures S3 and S4.

**Regression, sensitivity, and diagnostic analyses**

Logistic regression was used to estimate odds ratios for the hospitalization-level documented thrombotic outcome. PaO₂ was modeled continuously per 10-mmHg increase and categorically as ≤80, 81–100, and >100 mmHg. Four models were fitted: an unadjusted model; a demographic/comorbidity model; a model additionally including laboratory, catheter, and measurement-timing variables; and a fully adjusted model additionally including FiO₂, respiratory-support status, and non-respiratory first-day SOFA score. Estimates from the imputed datasets were combined using Rubin’s rules.

Sensitivity analyses included complete-case analysis, exclusion of PaO₂ values >400 mmHg, exclusion of values above the 99th percentile, restriction to each patient’s first eligible admission, the high-reliability FiO₂ subset, additional adjustment for race or ethnicity, broader and strict outcome definitions, and substitution of the PaO₂/FiO₂ ratio for PaO₂. Firth penalized logistic regression was additionally used for the strict outcome. Restricted cubic splines used knots at the 10th, 50th, and 90th percentiles of PaO₂ (80, 158, and 346 mmHg), with 80 mmHg as the reference. Exploratory interaction analyses were performed by measurement-time window, invasive mechanical ventilation status, and PICC status. Model diagnostics included apparent AUC, apparent Brier score, generalized variance inflation factors, leverage, Cook’s distance, and DFBETA statistics. Secondary and exploratory analyses were not adjusted for multiple comparisons and were interpreted as hypothesis-generating. An additional narrow-window sensitivity analysis restricted the cohort to admissions in which the selected PaO₂ measurement was obtained within 60 minutes after catheter insertion. Models 2 and 3 were refitted in this restricted cohort using the same multiple-imputation framework, patient-clustered HC0 robust standard errors, and Rubin’s-rules pooling as in the primary analysis; results are shown in Supplementary Table S12.

**Supplementary Table S1. Sensitivity analyses for continuous PaO₂ and the documented thrombotic outcome**

| **Analysis** | **Adjusted OR per 10-mmHg increase (95% CI)** | **P value** | **N** | **Events** |
| --- | --- | --- | --- | --- |
| Main analysis (Model 3) | 1.008 (0.989–1.027) | 0.430 | 11,277 | 195 |
| Model 3 without non-respiratory SOFA | 1.001 (0.982–1.020) | 0.959 | 11,277 | 195 |
| Complete-case analysis | 1.012 (0.989–1.034) | 0.306 | 8,906 | 139 |
| Excluding PaO₂ >400 mmHg | 1.007 (0.985–1.029) | 0.545 | 10,787 | 190 |
| Excluding PaO₂ above the 99th percentile | 1.005 (0.986–1.025) | 0.597 | 11,166 | 193 |
| First eligible admission per patient | 1.006 (0.987–1.026) | 0.514 | 10,789 | 186 |
| High-reliability FiO₂ subset | 1.017 (0.992–1.043) | 0.186 | 7,255 | 102 |
| Additional adjustment for race or ethnicity | 1.008 (0.989–1.027) | 0.426 | 11,277 | 195 |

The primary outcome was documented acute upper-body deep or central venous thrombosis during hospitalization. The high-reliability FiO₂ subset was restricted to admissions in which FiO₂ was obtained from the same blood gas record or a ventilator setting recorded within 60 minutes of PaO₂ measurement.

Except for the complete-case analysis, missing covariates were handled by multiple imputation by chained equations (20 datasets). Logistic regression models used patient-clustered HC0 robust standard errors, and estimates were combined using Rubin’s rules. All P values are two-sided.

Abbreviations: CI, confidence interval; FiO₂, fraction of inspired oxygen; OR, odds ratio; PaO₂, arterial partial pressure of oxygen; SOFA, Sequential Organ Failure Assessment.

**Supplementary Table S2. Sensitivity analyses for categorical PaO₂ and the documented thrombotic outcome**

| **Analysis** | **Comparison** | **Adjusted OR (95% CI)** | **P value** | **N** | **Events** |
| --- | --- | --- | --- | --- | --- |
| Main analysis (Model 3) | 81–100 vs ≤80 mmHg | 0.900 (0.531–1.526) | 0.695 | 11,277 | 195 |
| Main analysis (Model 3) | >100 vs ≤80 mmHg | 0.989 (0.652–1.502) | 0.960 | 11,277 | 195 |
| Model 3 without non-respiratory SOFA | 81–100 vs ≤80 mmHg | 0.895 (0.529–1.516) | 0.681 | 11,277 | 195 |
| Model 3 without non-respiratory SOFA | >100 vs ≤80 mmHg | 0.890 (0.585–1.356) | 0.588 | 11,277 | 195 |
| Complete-case analysis | 81–100 vs ≤80 mmHg | 0.987 (0.520–1.873) | 0.968 | 8,906 | 139 |
| Complete-case analysis | >100 vs ≤80 mmHg | 0.961 (0.566–1.632) | 0.883 | 8,906 | 139 |
| Excluding PaO₂ >400 mmHg | 81–100 vs ≤80 mmHg | 0.901 (0.531–1.528) | 0.699 | 10,787 | 190 |
| Excluding PaO₂ >400 mmHg | >100 vs ≤80 mmHg | 0.987 (0.649–1.500) | 0.950 | 10,787 | 190 |
| Excluding PaO₂ above the 99th percentile | 81–100 vs ≤80 mmHg | 0.899 (0.530–1.526) | 0.694 | 11,166 | 193 |
| Excluding PaO₂ above the 99th percentile | >100 vs ≤80 mmHg | 0.984 (0.648–1.495) | 0.939 | 11,166 | 193 |
| First eligible admission per patient | 81–100 vs ≤80 mmHg | 0.849 (0.497–1.449) | 0.548 | 10,789 | 186 |
| First eligible admission per patient | >100 vs ≤80 mmHg | 0.948 (0.624–1.441) | 0.804 | 10,789 | 186 |
| High-reliability FiO₂ subset | 81–100 vs ≤80 mmHg | 1.699 (0.823–3.505) | 0.152 | 7,255 | 102 |
| High-reliability FiO₂ subset | >100 vs ≤80 mmHg | 1.179 (0.628–2.214) | 0.609 | 7,255 | 102 |
| Additional adjustment for race or ethnicity | 81–100 vs ≤80 mmHg | 0.904 (0.532–1.536) | 0.709 | 11,277 | 195 |
| Additional adjustment for race or ethnicity | >100 vs ≤80 mmHg | 0.992 (0.653–1.507) | 0.971 | 11,277 | 195 |

PaO₂ ≤80 mmHg was the reference category in all analyses. The primary outcome was documented acute upper-body deep or central venous thrombosis during hospitalization. The high-reliability FiO₂ subset was restricted to admissions in which FiO₂ was obtained from the same blood gas record or a ventilator setting recorded within 60 minutes of PaO₂ measurement.

Except for the complete-case analysis, missing covariates were handled by multiple imputation by chained equations (20 datasets). Logistic regression models used patient-clustered HC0 robust standard errors, and estimates were combined using Rubin’s rules. All P values are two-sided.

Abbreviations: CI, confidence interval; FiO₂, fraction of inspired oxygen; OR, odds ratio; PaO₂, arterial partial pressure of oxygen; SOFA, Sequential Organ Failure Assessment.

**Supplementary Table S3. Alternative outcome and exposure analyses**

| **Outcome** | **Method** | **Exposure or comparison** | **Adjusted OR (95% CI)** | **P value** | **N** | **Events** |
| --- | --- | --- | --- | --- | --- | --- |
| Broader secondary outcome | Clustered logistic regression | PaO₂ per 10-mmHg increase | 1.002 (0.986–1.018) | 0.814 | 11,277 | 282 |
| Broader secondary outcome | Clustered logistic regression | 81–100 vs ≤80 mmHg | 0.817 (0.530–1.260) | 0.360 | 11,277 | 282 |
| Broader secondary outcome | Clustered logistic regression | >100 vs ≤80 mmHg | 0.895 (0.637–1.257) | 0.521 | 11,277 | 282 |
| Strict outcome | Clustered logistic regression | PaO₂ per 10-mmHg increase | 1.008 (0.974–1.042) | 0.661 | 11,277 | 69 |
| Strict outcome | Clustered logistic regression | 81–100 vs ≤80 mmHg | 0.455 (0.187–1.109) | 0.083 | 11,277 | 69 |
| Strict outcome | Clustered logistic regression | >100 vs ≤80 mmHg | 0.747 (0.401–1.392) | 0.359 | 11,277 | 69 |
| Strict outcome | Firth penalized logistic regression | PaO₂ per 10-mmHg increase | 1.009 (0.981–1.037) | 0.546 | 11,277 | 69 |
| Strict outcome | Firth penalized logistic regression | 81–100 vs ≤80 mmHg | 0.473 (0.202–1.106) | 0.084 | 11,277 | 69 |
| Strict outcome | Firth penalized logistic regression | >100 vs ≤80 mmHg | 0.734 (0.415–1.297) | 0.287 | 11,277 | 69 |
| Primary outcome | Clustered logistic regression | PaO₂/FiO₂ ratio per 50-unit increase | 1.010 (0.957–1.065) | 0.730 | 11,277 | 195 |

The broader secondary outcome included any documented upper-extremity venous thrombosis, including superficial or anatomically unspecified thrombosis. The strict outcome was restricted to primary-outcome diagnoses with laterality potentially compatible with the qualifying catheter (right-sided diagnosis with right-sided catheter, left-sided diagnosis with left-sided catheter, or bilateral diagnosis); diagnoses with unspecified laterality were excluded from the strict definition. Hospital-level diagnosis codes could not establish that an event was caused by the qualifying catheter or occurred after the selected PaO₂ measurement.

For Firth penalized logistic regression, estimates were pooled across imputations without patient-clustered robust standard errors. In the PaO₂/FiO₂ analysis, PaO₂ and FiO₂ were not entered separately in the model. PaO₂ ≤80 mmHg was the reference category for categorical comparisons.

Abbreviations: CI, confidence interval; FiO₂, fraction of inspired oxygen; OR, odds ratio; PaO₂, arterial partial pressure of oxygen.

**Supplementary Table S4. Subgroup analyses of continuous PaO₂ and the documented thrombotic outcome**

| **Subgroup** | **Level** | **N** | **Events** | **Adjusted OR per 10-mmHg increase (95% CI)** | **P value** | **P for interaction** |
| --- | --- | --- | --- | --- | --- | --- |
| Measurement-time window | ≤1 h | 6,301 | 49 | 1.021 (0.992–1.051) | 0.167 | 0.223 |
| Measurement-time window | >1–6 h | 3,759 | 98 | 0.990 (0.961–1.019) | 0.485 |  |
| Measurement-time window | >6–24 h | 1,217 | 48 | 1.024 (0.987–1.063) | 0.212 |  |
| Invasive ventilation | No identified invasive ventilation | 3,907 | 57 | 1.017 (0.988–1.046) | 0.261 | 0.522 |
| Invasive ventilation | Invasive mechanical ventilation | 7,370 | 138 | 1.005 (0.983–1.028) | 0.651 |  |
| PICC status | Non-PICC | 10,735 | 158 | 1.005 (0.985–1.026) | 0.617 | 0.453 |
| PICC status | PICC | 542 | 37 | 1.024 (0.978–1.073) | 0.305 |  |

Subgroup analyses used the fully adjusted Model 3 covariates and continuous PaO₂. Interaction P values evaluated effect modification by the corresponding subgroup variable. Estimates were combined across 20 imputed datasets using Rubin’s rules with patient-clustered robust standard errors.

Abbreviations: CI, confidence interval; OR, odds ratio; PaO₂, arterial partial pressure of oxygen; PICC, peripherally inserted central catheter.

**Supplementary Table S5. Model diagnostics and influence analysis**

| **Diagnostic** | **Value** | **Details** |
| --- | --- | --- |
| Apparent AUC (C-statistic) | 0.7834 (range, 0.7801–0.7872) | Mean across 20 imputed datasets |
| Apparent Brier score | 0.0166 (range, 0.0166–0.0167) | Null-model Brier score, 0.0170 |
| Scaled Brier score | 0.0207 (range, 0.0198–0.0227) | Relative improvement over the null model |
| Maximum adjusted GVIF | 1.318 | No substantial multicollinearity |
| Maximum leverage | 0.0580 | Representative completed dataset |
| Maximum Cook's distance | 0.0148 | Representative completed dataset |
| Maximum overall \|DFBETA\| | 0.884 | Two admissions exceeded 0.5 |
| Maximum PaO₂-specific \|DFBETA\| | 0.390 | Below the prespecified 0.5 threshold |
| Model 3 after excluding 2 influential admissions | OR, 1.006 (95% CI, 0.987–1.025); P=0.555 | N=11,275; events=193 |

Discrimination and Brier statistics are apparent values calculated in the development sample and were not corrected for optimism. Apparent calibration intercept and slope were 0 and 1, respectively, as expected when a logistic model is assessed in the same data used for fitting; these values do not establish calibration quality. Influence and multicollinearity diagnostics were evaluated in a representative completed dataset.

Abbreviations: AUC, area under the receiver operating characteristic curve; CI, confidence interval; DFBETA, change in a regression coefficient after deletion of an observation; GVIF, generalized variance inflation factor; OR, odds ratio; PaO₂, arterial partial pressure of oxygen.

**Supplementary Table S6. Missing data and multiple-imputation summary**

| **Variable** | **Observed N** | **Missing, n (%)** | **Observed median** | **Completed-data median** | **Imputation method** |
| --- | --- | --- | --- | --- | --- |
| Hemoglobin, g/dL | 10,844 | 433 (3.8) | 10.5 | 10.5 | Predictive mean matching |
| Platelet count, ×10⁹/L | 10,883 | 394 (3.5) | 160 | 160 | Predictive mean matching |
| Lactate, mmol/L | 10,678 | 599 (5.3) | 2.2 | 2.2 | Predictive mean matching |
| Non-respiratory SOFA score | 10,362 | 915 (8.1) | 5 | 5 | Predictive mean matching |
| FiO₂, % | 10,067 | 1,210 (10.7) | 60 | 60 | Predictive mean matching |
| Catheter laterality | 11,267 | 10 (0.1) | — | — | Logistic regression |

Multiple imputation by chained equations generated 20 completed datasets using 20 iterations and seed 20260717. Continuous variables were imputed using predictive mean matching; the small number of missing catheter-laterality values were imputed using logistic regression. The exposure and outcome were not imputed.

The final validation included 284 checks, all of which passed. Logged collinearity events were confined to the FiO₂-source indicator; this indicator was excluded from predicting FiO₂ in the predictor matrix.

Abbreviations: FiO₂, fraction of inspired oxygen; SOFA, Sequential Organ Failure Assessment.

**Supplementary Table S7. Post hoc module-specific analysis of changes in the PaO₂ estimate**

| **Model specification** | **Added covariates** | **PaO₂ OR per 10-mmHg increase (95% CI)** | **P value** | **β change vs Model 1** |
| --- | --- | --- | --- | --- |
| Model 1 | Age, sex, and Charlson Comorbidity Index | 0.975 (0.957–0.993) | 0.006 | Reference |
| Model 1 + catheter/timing module | Catheter type, laterality, multiple active catheters, and ln(minutes+1) | 1.004 (0.985–1.023) | 0.687 | +0.030 |
| Model 1 + laboratory module | Hemoglobin, platelet count, and lactate | 0.974 (0.956–0.992) | 0.006 | −0.000 |
| Model 1 + oxygenation/severity module | FiO₂, respiratory support, and non-respiratory SOFA | 0.992 (0.973–1.011) | 0.388 | +0.018 |
| Model 3 | All covariate modules entered simultaneously | 1.008 (0.989–1.027) | 0.430 | +0.033 |

Each covariate module was added separately to Model 1; the module-specific models were therefore parallel rather than sequential. Model 3 included all covariate modules simultaneously. The analysis was exploratory and descriptive and cannot identify any individual covariate as responsible for the change in the PaO₂ estimate.

Abbreviations: CI, confidence interval; FiO₂, fraction of inspired oxygen; OR, odds ratio; PaO₂, arterial partial pressure of oxygen; SOFA, Sequential Organ Failure Assessment.

**Supplementary Table S8. Associations of all covariates in the fully adjusted model with the documented thrombotic outcome**

| **Variable** | **Comparison or unit** | **Adjusted OR (95% CI)** | **P value** |
| --- | --- | --- | --- |
| PaO₂ | Per 10-mmHg increase | 1.008 (0.989–1.027) | 0.430 |
| Age | Per year | 0.992 (0.980–1.004) | 0.195 |
| Sex | Male vs female | 0.941 (0.696–1.273) | 0.693 |
| Charlson Comorbidity Index | Per point | 1.044 (0.978–1.115) | 0.196 |
| Hemoglobin | Per 1 g/dL | 0.971 (0.902–1.046) | 0.439 |
| Platelet count | Per 1 ×10⁹/L | 1.001 (1.000–1.002) | 0.099 |
| Lactate | Per 1 mmol/L | 1.055 (0.990–1.125) | 0.099 |
| Catheter type | Large-bore/specialized vs multi-lumen | 0.437 (0.240–0.795) | 0.007 |
| Catheter type | Other vs multi-lumen | 0.979 (0.644–1.488) | 0.919 |
| Catheter type | PICC vs multi-lumen | 2.713 (1.766–4.167) | <0.001 |
| Catheter laterality | Left vs right | 1.429 (1.010–2.020) | 0.044 |
| Multiple active catheters | Multiple vs one | 1.051 (0.667–1.656) | 0.830 |
| Time from catheter insertion to PaO₂ | Per one-unit increase in ln(minutes+1) | 1.326 (1.152–1.526) | <0.001 |
| FiO₂ at PaO₂ measurement | Per 1 percentage point | 1.000 (0.993–1.008) | 0.938 |
| Respiratory support | Other recorded support vs invasive mechanical ventilation | 0.613 (0.340–1.104) | 0.103 |
| Respiratory support | No contemporaneous support interval identified vs invasive mechanical ventilation | 0.789 (0.549–1.132) | 0.198 |
| Non-respiratory SOFA score | Per point | 1.147 (1.093–1.203) | <0.001 |

The outcome was documented acute upper-body deep or central venous thrombosis during hospitalization. Model 3 included all variables shown in the table. Missing covariates were handled using multiple imputation by chained equations (20 datasets); logistic regression used patient-clustered HC0 robust standard errors, and estimates were combined using Rubin’s rules.

Reference categories were female sex, multi-lumen catheter, right-sided catheter placement, one active qualifying catheter, and invasive mechanical ventilation. Associations shown for covariates were secondary findings and should not be interpreted as causal effects or independent risk factors.

Abbreviations: CI, confidence interval; FiO₂, fraction of inspired oxygen; OR, odds ratio; PaO₂, arterial partial pressure of oxygen; PICC, peripherally inserted central catheter; SOFA, Sequential Organ Failure Assessment.

**Supplementary Table S9. Pairwise standardized mean differences for baseline characteristics across PaO₂ categories**

| **Characteristic** | **SMD ≤80 vs 81–100** | **SMD ≤80 vs >100** | **SMD 81–100 vs >100** |
| --- | --- | --- | --- |
| Age, years | 0.108 | 0.141 | 0.034 |
| Charlson Comorbidity Index | 0.010 | 0.163 | 0.158 |
| Hemoglobin, g/dL | 0.081 | 0.031 | 0.056 |
| Platelet count, ×10⁹/L | 0.075 | 0.302 | 0.240 |
| Baseline lactate, mmol/L | 0.000 | 0.060 | 0.059 |
| Nonrespiratory SOFA score | 0.144 | 0.511 | 0.367 |
| FiO₂, % | 0.142 | 0.277 | 0.415 |
| Time from catheter insertion to PaO₂ measurement, min | 0.189 | 0.514 | 0.362 |
| PaO₂, mmHg | 2.667 | 2.170 | 1.852 |
| Sex |  |  |  |
| Male | 0.067 | 0.076 | 0.010 |
| Female | 0.067 | 0.076 | 0.010 |
| Race or ethnicity |  |  |  |
| White | 0.095 | 0.126 | 0.031 |
| Black | 0.114 | 0.111 | 0.003 |
| Hispanic or Latino | 0.062 | 0.013 | 0.075 |
| Asian | 0.075 | 0.009 | 0.066 |
| Other | 0.033 | 0.000 | 0.033 |
| Unknown or not reported | 0.003 | 0.086 | 0.083 |
| Respiratory support at PaO₂ measurement |  |  |  |
| Invasive mechanical ventilation | 0.120 | 0.206 | 0.086 |
| Other recorded respiratory support | 0.210 | 0.507 | 0.320 |
| No contemporaneous respiratory-support interval identified | 0.043 | 0.145 | 0.103 |
| Catheter type |  |  |  |
| PICC | 0.151 | 0.330 | 0.189 |
| Multi-lumen catheter | 0.074 | 0.409 | 0.337 |
| Large-bore or specialized catheter | 0.199 | 0.582 | 0.394 |
| Other qualifying catheter type | 0.012 | 0.072 | 0.059 |
| Catheter laterality |  |  |  |
| Right | 0.121 | 0.301 | 0.183 |
| Left | 0.125 | 0.302 | 0.179 |
| Unknown | 0.034 | 0.007 | 0.040 |
| Number of active qualifying catheters |  |  |  |
| One active catheter | 0.264 | 0.621 | 0.368 |
| Multiple active catheters | 0.264 | 0.621 | 0.368 |
| Primary outcome |  |  |  |
| Documented event, n (%) | 0.042 | 0.088 | 0.047 |

SMD denotes the absolute standardized mean difference. For continuous variables, SMDs were calculated using means and pooled standard deviations; for categorical variables, SMDs were calculated from proportions.

Abbreviations: FiO₂, fraction of inspired oxygen; PaO₂, arterial partial pressure of oxygen; PICC, peripherally inserted central catheter; SMD, standardized mean difference; SOFA, Sequential Organ Failure Assessment.

**Supplementary Table S10. ICD-9-CM and ICD-10-CM codes used to define documented upper-body venous thrombosis outcomes**

| **ICD version** | **Code** | **Vein group** | **Laterality** | **Primary outcome** | **Broader outcome** | **Description** |
| --- | --- | --- | --- | --- | --- | --- |
| 9 | 45381 | Superficial | Unspecified | Not included | Included | Acute venous embolism and thrombosis of superficial veins of upper extremity |
| 9 | 45382 | Deep | Unspecified | Included | Included | Acute venous embolism and thrombosis of deep veins of upper extremity |
| 9 | 45383 | Unspecified | Unspecified | Not included | Included | Acute venous embolism and thrombosis of upper extremity, unspecified |
| 9 | 45384 | Axillary | Unspecified | Included | Included | Acute venous embolism and thrombosis of axillary veins |
| 9 | 45385 | Subclavian | Unspecified | Included | Included | Acute venous embolism and thrombosis of subclavian veins |
| 9 | 45386 | Internal jugular | Unspecified | Included | Included | Acute venous embolism and thrombosis of internal jugular veins |
| 10 | I82601 | Unspecified | Right | Not included | Included | Acute embolism and thrombosis of unspecified veins of right upper extremity |
| 10 | I82602 | Unspecified | Left | Not included | Included | Acute embolism and thrombosis of unspecified veins of left upper extremity |
| 10 | I82603 | Unspecified | Bilateral | Not included | Included | Acute embolism and thrombosis of unspecified veins of upper extremity, bilateral |
| 10 | I82609 | Unspecified | Unspecified | Not included | Included | Acute embolism and thrombosis of unspecified veins of unspecified upper extremity |
| 10 | I82611 | Superficial | Right | Not included | Included | Acute embolism and thrombosis of superficial veins of right upper extremity |
| 10 | I82612 | Superficial | Left | Not included | Included | Acute embolism and thrombosis of superficial veins of left upper extremity |
| 10 | I82613 | Superficial | Bilateral | Not included | Included | Acute embolism and thrombosis of superficial veins of upper extremity, bilateral |
| 10 | I82619 | Superficial | Unspecified | Not included | Included | Acute embolism and thrombosis of superficial veins of unspecified upper extremity |
| 10 | I82621 | Deep | Right | Included | Included | Acute embolism and thrombosis of deep veins of right upper extremity |
| 10 | I82622 | Deep | Left | Included | Included | Acute embolism and thrombosis of deep veins of left upper extremity |
| 10 | I82623 | Deep | Bilateral | Included | Included | Acute embolism and thrombosis of deep veins of upper extremity, bilateral |
| 10 | I82629 | Deep | Unspecified | Included | Included | Acute embolism and thrombosis of deep veins of unspecified upper extremity |
| 10 | I82A11 | Axillary | Right | Included | Included | Acute embolism and thrombosis of right axillary vein |
| 10 | I82A12 | Axillary | Left | Included | Included | Acute embolism and thrombosis of left axillary vein |
| 10 | I82A13 | Axillary | Bilateral | Included | Included | Acute embolism and thrombosis of axillary vein, bilateral |
| 10 | I82A19 | Axillary | Unspecified | Included | Included | Acute embolism and thrombosis of unspecified axillary vein |
| 10 | I82B11 | Subclavian | Right | Included | Included | Acute embolism and thrombosis of right subclavian vein |
| 10 | I82B12 | Subclavian | Left | Included | Included | Acute embolism and thrombosis of left subclavian vein |
| 10 | I82B13 | Subclavian | Bilateral | Included | Included | Acute embolism and thrombosis of subclavian vein, bilateral |
| 10 | I82B19 | Subclavian | Unspecified | Included | Included | Acute embolism and thrombosis of unspecified subclavian vein |
| 10 | I82C11 | Internal jugular | Right | Included | Included | Acute embolism and thrombosis of right internal jugular vein |
| 10 | I82C12 | Internal jugular | Left | Included | Included | Acute embolism and thrombosis of left internal jugular vein |
| 10 | I82C13 | Internal jugular | Bilateral | Included | Included | Acute embolism and thrombosis of internal jugular vein, bilateral |
| 10 | I82C19 | Internal jugular | Unspecified | Included | Included | Acute embolism and thrombosis of unspecified internal jugular vein |

Notes: The table lists the exact acute ICD codes used in the primary or broader outcome definitions. The primary outcome included acute deep, axillary, subclavian, and internal jugular venous thrombosis codes. The broader secondary outcome included all acute codes shown, including superficial and anatomically unspecified upper-extremity venous thrombosis. Chronic codes were excluded from both acute outcome definitions. The strict sensitivity outcome used the primary definition and additionally required laterality potentially compatible with the qualifying catheter: a right-sided diagnosis with a right-sided catheter, a left-sided diagnosis with a left-sided catheter, or a bilateral diagnosis. Diagnoses with unspecified laterality did not satisfy the strict definition. This admission-level rule cannot be represented by the ICD code alone.

**Supplementary Table S11. Timing characteristics and upper-tail distribution of PaO₂**

| **Section** | **Group or threshold** | **N** | **Measure** | **Value** |
| --- | --- | --- | --- | --- |
| Timing by primary-outcome status | No documented primary outcome | 11,082 | ICU admission to selected PaO₂, min | 299 (176–525) |
| Timing by primary-outcome status | Documented primary outcome | 195 | ICU admission to selected PaO₂, min | 494 (232–1,160) |
| Timing by primary-outcome status | No documented primary outcome | 11,082 | Catheter index time to selected PaO₂, min | 45 (22–152) |
| Timing by primary-outcome status | Documented primary outcome | 195 | Catheter index time to selected PaO₂, min | 134 (60.5–339) |
| Upper-tail PaO₂ distribution | PaO₂ >384 mmHg | 647 | Documented primary-outcome events | 10 |
| Upper-tail PaO₂ distribution | PaO₂ >400 mmHg | 490 | Documented primary-outcome events | 5 |
| Upper-tail PaO₂ distribution | PaO₂ >99th percentile (>461 mmHg) | 111 | Documented primary-outcome events | 2 |
| Overall PaO₂ distribution | All admissions | 11,277 | PaO₂, mmHg | 158 (106–255); range, 16–586 |

Continuous values are presented as median (interquartile range) unless otherwise indicated. The ICU-admission timing summaries and upper-tail counts were additional post hoc descriptive analyses. The catheter index time was the start of the qualifying catheter episode. Because thrombosis diagnoses were available only at the hospitalization level and lacked reliable onset timestamps, the interval from PaO₂ measurement to thrombus onset could not be calculated.

**Supplementary Table S12. Sensitivity analysis restricted to PaO₂ measurements obtained within 60 minutes after catheter insertion**

| **Model** | **Admissions, n** | **Primary outcome events, n** | **OR per 10-mmHg increase in PaO₂** | **95% CI** | **P value** |
| --- | --- | --- | --- | --- | --- |
| Model 2 | 6,301 | 49 | 1.026 | 0.995–1.058 | 0.102 |
| Model 3 | 6,301 | 49 | 1.041 | 1.010–1.073 | 0.010 |

Model 2 was adjusted for age, sex, Charlson Comorbidity Index, hemoglobin, platelet count, lactate, catheter type, catheter laterality, multiple active catheters, and the log-transformed interval from catheter insertion to PaO₂ measurement. Model 3 additionally included FiO₂, respiratory-support status, and the non-respiratory first-day SOFA score. Estimates were obtained using the same multiple-imputation framework as the primary analysis, with patient-clustered HC0 robust standard errors and Rubin’s-rules pooling. All P values are two-sided.

Abbreviations: CI, confidence interval; FiO₂, fraction of inspired oxygen; OR, odds ratio; PaO₂, arterial partial pressure of oxygen; SOFA, Sequential Organ Failure Assessment.

**Supplementary Figure S1. Subgroup-specific associations between PaO₂ and the documented thrombotic outcome**

Points represent adjusted odds ratios for the documented thrombotic outcome per 10-mmHg increase in PaO₂, and horizontal lines represent 95% confidence intervals. Models included the covariates specified in Model 3 and the corresponding interaction term. Interaction P values test whether the PaO₂ association differed across subgroup levels. Estimates were combined across 20 multiply imputed datasets using Rubin’s rules with patient-clustered robust standard errors. Abbreviations: CI, confidence interval; OR, odds ratio; PaO₂, arterial partial pressure of oxygen; PICC, peripherally inserted central catheter.

**Supplementary Figure S2. Apparent calibration of the fully adjusted Model 3**

The plot compares observed and predicted probabilities in the development sample using the first completed dataset as a representative dataset. The dashed line represents perfect agreement and the smooth curve represents apparent calibration. The plot was not corrected for optimism by bootstrap resampling and does not constitute internal or external validation. The apparent intercept of 0 and slope of 1 are mathematical properties of evaluating a fitted logistic model in its development data.

**Supplementary Figure S3. Trace plots for the multiple-imputation procedure**

**Supplementary Figure S3. Trace plots for the multiple-imputation procedure (continued)**

**Supplementary Figure S3. Trace plots for the multiple-imputation procedure (continued)**

**Supplementary Figure S3. Trace plots for the multiple-imputation procedure (continued)**

Trace plots show the iteration histories of the mean and standard deviation for imputed variables across 20 imputation chains. The plots were used as graphical diagnostics for chain mixing and stability during 20 iterations. Abbreviations: FiO₂, fraction of inspired oxygen; SOFA, Sequential Organ Failure Assessment.

**Supplementary Figure S4. Distributional diagnostics for observed and imputed values**

**Supplementary Figure S4. Distributional diagnostics for observed and imputed values (continued)**

**Supplementary Figure S4. Distributional diagnostics for observed and imputed values (continued)**

**Supplementary Figure S4. Distributional diagnostics for observed and imputed values (continued)**

**Supplementary Figure S4. Distributional diagnostics for observed and imputed values (continued)**

Density plots compare the distributions of observed values with those generated across the 20 imputed datasets for hemoglobin, platelet count, lactate, non-respiratory SOFA score, and FiO₂. The overlaid curves were inspected for major distributional discrepancies between observed and imputed values. Abbreviations: FiO₂, fraction of inspired oxygen; SOFA, Sequential Organ Failure Assessment.
