# Supplementary material for: Association between early post-insertion arterial oxygen tension and documented acute upper-body deep or central venous thrombosis in critically ill adults: A retrospective MIMIC-IV cohort study
Source: PLoS One. 2026 Oct 5;21(10):e0359719. doi: 10.1371/journal.pone.0359719 (PMC13637912; doi:10.1371/journal.pone.0359719)
Supplement: Supplementary file 2 [file pone.0359719.s003.docx]

Manuscript ID: PONE-D-25-27701R1

Dear Editor,

Thank you very much for the invitation to review this manuscript titled “Higher Partial Pressure of Oxygen is Associated with Reduced Risk of Catheter-Related Thrombosis in Critical Care: Analysis of Threshold Effects Using the MIMIC Database” and for the opportunity to contribute to the evaluation process for PLOS ONE. I would also like to thank the authors for their work on a clinically relevant question and for the careful, point-by-point responses provided in this revised version, which have clearly improved the clarity of the manuscript.

In brief, the authors use the MIMIC-IV database (2008–2019) to investigate the association between arterial partial pressure of oxygen (PaO₂) and catheter-related thrombosis (CRT) in 13,328 adult ICU patients with upper-extremity catheters. PaO₂ was analysed both as a continuous variable and in three categories (≤80, >80–100, >100 mmHg), with progressively adjusted logistic regression models, smooth-curve fitting, segmented (threshold) regression, and subgroup analyses by anticoagulation status, SOFA score and age. Compared with PaO₂ ≤80 mmHg, higher PaO₂ categories were associated with a lower adjusted risk of CRT (adjusted OR 0.49 and 0.47), and the authors report a threshold at 384 mmHg beyond which no further benefit was seen. The revised manuscript reframes this finding as avoidance of hypoxemia rather than induction of hyperoxia, which is appropriate.

My recommendation is MAJOR REVISION. The dataset and question are of interest and the writing has improved, but several methodological and interpretive issues need to be resolved before the conclusions can be considered robust. My specific concerns, in order of importance, are as follows:

**1.** In Table 3, the effect above the 384 mmHg breakpoint is reported as OR = 0.00 (95% CI 0.00, Inf), p = 0.9795. An odds ratio of zero with an infinite upper bound indicates complete separation / absence of events (or vanishingly few patients) above the breakpoint, i.e. a non-interpretable estimate rather than evidence of a true plateau. Because this threshold now features prominently in both the title and the abstract, it must be substantiated: please report the number of patients and the number of CRT events above 384 mmHg, and reconsider whether the segmented model is adequately powered in that range. If the estimate is driven by sparse data, the threshold should be presented with strong caveats or removed from the title/abstract.

2. When PaO₂ is modelled continuously, the adjusted OR is 1.00 (95% CI 0.99–1.00) per 1 mmHg, with significance driven almost entirely by the large sample size. Describing this as a “significant inverse association” risks overstating a near-null per-unit effect. Please report the OR per a clinically meaningful increment (e.g. per 10 mmHg) and temper the wording accordingly.

3. Patients with PaO₂ >100 mmHg constitute the large majority of the cohort (9,242/13,328) and very likely reflect patients receiving supplemental oxygen or mechanical ventilation, whereas low PaO₂ marks respiratory failure and greater illness. Although APSIII and SOFA were adjusted for, key drivers such as FiO₂, mechanical ventilation, and the PaO₂/FiO₂ ratio are not included. A PaO₂ of 384 mmHg is essentially attainable on high FiO₂, so the exposure is partly a proxy for the treatment received. Please adjust for FiO₂ / ventilation where available, or discuss this as a major source of residual confounding and reverse causation.

4. CRT was identified from ICD-9/10 codes and clinical notes. By the authors’ own cited literature, a large fraction of CRT is asymptomatic (incidence reported up to 59%), yet the observed incidence here is only ~2.2% in the reference group. This strongly suggests that only symptomatic / imaged thrombosis was captured, and detection may be differential (sicker or more frequently imaged patients are more likely to be diagnosed). Please report the total number of CRT events, and discuss the direction of this detection bias; it could plausibly generate the observed association if low-PaO₂ patients are imaged more often.

5. I appreciate the clarification that the first PaO₂ before CRT was used. However, a single baseline value cannot capture the dynamic oxygenation of an ICU stay, and the differing exposure windows introduce an immortal-time-like imbalance. A time-to-event model with PaO₂ as a time-varying exposure would be more appropriate; at minimum, this limitation should be stated explicitly.

6. Reporting completeness (STROBE). Please add the number of patients excluded for missing key variables and a comparison of their characteristics; the number of CRT events per PaO₂ group; and some assessment of model performance (calibration/discrimination). This information is needed to judge the stability of the adjusted estimates given the low event rate.

7. The biological rationale leans heavily on COVID-19 cohorts (e.g. Helms et al., Tang et al.). Coagulopathy in severe SARS-CoV-2 infection may not generalise to unselected ICU patients with catheter-related thrombosis; please acknowledge this and, where possible, cite mechanistic evidence from a broader critical-care population.

A few minor points would also strengthen the manuscript:

8. Table 1 still contains misaligned and internally inconsistent cells; please verify all values, units, and decimal places.

9. The notation alternates between “PO2” and “PaO₂” throughout the text, tables and figure legends; please standardise to PaO₂.

10. Figure 2 should state the number of observations and ideally show the distribution of PaO₂ values, given the wide confidence bands at the extremes.

In summary, this is a potentially useful real-world analysis, but the threshold claim that anchors the title and abstract currently rests on a non-estimable result, and the association is vulnerable to confounding by ventilation and to outcome misclassification.

Addressing points these points is essential before the conclusions can be supported. I believe these issues are addressable, and I would be glad to review a revised version.

I thank the Editor again for the invitation and the authors for their efforts on this work.

Sincerely,

The Reviewer
