# Supplementary material for: Association between early post-insertion arterial oxygen tension and documented acute upper-body deep or central venous thrombosis in critically ill adults: A retrospective MIMIC-IV cohort study
Source: PLoS One. 2026 Oct 5;21(10):e0359719. doi: 10.1371/journal.pone.0359719 (PMC13637912; doi:10.1371/journal.pone.0359719)
Supplement: Supplementary file 3 [file pone.0359719.s004.docx]

**Response to Reviewers**
Manuscript ID: PONE-D-25-27701
Title: *Higher Partial Pressure of Oxygen is Associated with Reduced Risk of Catheter-Related Thrombosis in Critical Care: Analysis of Threshold Effects Using the MIMIC Database*

Dear Academic Editor and Reviewers,

Thank you very much for your thoughtful and constructive feedback on our manuscript. We greatly appreciate the time and expertise you have devoted to reviewing our work. We have carefully considered all comments and have revised the manuscript accordingly. Below, we provide a point-by-point response to each concern raised.

**Response to Reviewer #1**

**Comment 1**: *The authors failed to clarify the temporal relationship between PaO₂ and CRT development… I strongly urge the authors to clearly and specifically detail the inclusion criteria and the timing of data collection.*

We sincerely thank the reviewer for this crucial observation and apologize for the lack of clarity in our original manuscript.

We fully understand the reviewer’s concern. Establishing a valid temporal sequence—where exposure (PaO₂) precedes outcome (CRT)—is fundamental for causal inference in observational studies. Without this clarification, the validity of our conclusions could be questioned.

We confirm that all PaO₂ values used in our analysis were extracted from the first arterial blood gas measurement recorded after ICU admission and, critically, before the diagnosis of CRT (or before ICU discharge for patients who did not develop CRT). This ensures that the exposure temporally precedes the outcome.

**We have revised the manuscript as follows**:

**Original**: “The primary exposure variable was arterial oxygen partial pressure (PaO2), which was categorized into three groups… These measurements were obtained from arterial blood gas analyses during the ICU stay.”

**Revised**: “The primary exposure variable was the first recorded arterial oxygen partial pressure (PaO₂) during the ICU stay, which was confirmed to have occurred prior to the diagnosis of catheter-related thrombosis. This PaO₂ value was categorized into three groups: ≤80 mmHg, >80 to ≤100 mmHg, and >100 mmHg.”

Additionally, we added a sentence in the Statistical Analysis section: “All exposure data (PaO₂) were ascertained before the outcome event (CRT) to establish a valid temporal sequence.”

**Comment 2**: *There is a clear disconnect between the title and the actual content… I suggest revising the title.*

We thank the reviewer for this helpful suggestion.

We agree that the original title could be misleading, as it labeled PaO₂ a “risk factor,” whereas our findings indicate it is a protective factor (higher PaO₂ associated with lower CRT risk).

We have revised the title to more accurately reflect our findings:

**Original**: “Partial Pressure of Oxygen as a Risk Factor for Catheter-Related Thrombosis in Critical Care…”

**Revised**: “Higher Partial Pressure of Oxygen is Associated with Reduced Risk of Catheter-Related Thrombosis in Critical Care: Analysis of Threshold Effects Using the MIMIC Database”.

**Comment 3**: *The threshold of 384 mmHg is far higher than clinical values… clinical relevance is highly questionable.*

We sincerely appreciate the reviewer’s insightful clinical perspective and acknowledge this important concern.

We understand that a PaO₂ of 384 mmHg is supraphysiological and rarely a clinical target, as it typically requires high-concentration oxygen therapy, which carries risks of oxygen toxicity.

In our revision, we clarify that this threshold should be interpreted as a statistical inflection point—not a therapeutic goal. The key clinical message is that maintaining PaO₂ above hypoxemic levels (e.g., >80 mmHg) is protective, and no additional CRT benefit is seen beyond extremely high levels. We now explicitly caution against pursuing supraphysiological PaO₂ for CRT prevention alone.

**Revised text in the Discussion:**

**Original**: “Our findings provide valuable clinical reference for oxygen therapy management in ICU patients. The identification of a 384 mmHg PO2 threshold enables clinicians to optimize individualized oxygen therapy protocols…”

Revised: “However, our identification of a PO₂ threshold at 384 mmHg should be interpreted with caution. This value far exceeds typical clinical oxygenation targets and is often only achievable with high or pure oxygen supplementation, which carries its own risks of oxygen toxicity and lung injury(11). Therefore, this threshold should be viewed primarily as a statistical inflection point indicating the saturation of the protective effect against CRT, rather than a therapeutic goal. The more clinically relevant and actionable finding is that maintaining PO₂ above the hypoxemic range (e.g., >80 mmHg) is consistently associated with a significantly reduced risk of CRT. Clinicians should aim for adequate oxygenation to avoid hypoxemia, but should not pursue supraphysiological PO₂ levels solely for the purpose of CRT prevention, given the potential for harm from hyperoxia. Several publications have highlighted the harmful effects of hyperoxia in critically ill patients, including increased oxidative stress, ventilator-induced lung injury, and higher mortality in subgroups such as those with acute respiratory distress syndrome (ARDS) or post-cardiac arrest(12-14). For instance, Girardis et al. (12) in a randomized trial showed that conservative oxygen therapy (targeting PaO₂ 70-100 mmHg) reduced mortality compared to liberal strategies (PaO₂ up to 150 mmHg). This underscores the need to avoid hyperoxia while preventing hypoxemia. The observed association between hypoxemia and increased thrombosis risk is biologically plausible and aligns with previous mechanistic studies. Under hypoxic conditions, the activation of Hypoxia-Inducible Factors (HIFs) promotes thrombosis, though current risk models do not include oxygenation status as a specific factor (20)(21). Our study suggests that a patient's oxygenation level could be a relevant variable to consider in future risk stratification models. Further prospective studies are warranted to determine whether optimizing oxygenation, while strictly avoiding both hypoxia and hyperoxia, can be an effective component of a multifaceted strategy to prevent CRT in ICU patients**.**”

**Revised text in the conclusion**

**Original:** Higher PO2 levels are associated with reduced CRT risk in ICU patients, with an optimal threshold at 384 mmHg. These findings suggest the importance of maintaining adequate oxygenation for CRT prevention while avoiding excessive oxygen supplementation.

**Revised**: Our findings suggest that avoiding hypoxemia—not inducing hyperoxia—is the clinically relevant strategy for CRT prevention

**Response to Reviewer #2**

**Comment 1**: *Discuss the harmful effects of hyperoxia in critically ill patients.*

We sincerely appreciate the reviewer’s suggestion to expand the discussion on the harmful effects of hyperoxia in critically ill patients, as this ensures a more balanced and clinically relevant presentation.

In response, we have expanded the Discussion section to include a dedicated paragraph on hyperoxia's adverse effects, incorporating additional references to key publications for a comprehensive view.

**Revised text in the introduction**

Original: However, excessive oxygen levels may also have detrimental effects, highlighting the complexity of this relationship.

Revised: However, excessive oxygen levels may also have detrimental effects, highlighting the complexity of this relationship(11). Nevertheless, hyperoxia can lead to adverse outcomes such as oxidative stress and lung injury, necessitating a balanced approach(12-14).

**Revised text in the discussion**

This is now integrated into our interpretation of the 384 mmHg threshold (see response to Reviewer #1, Comment 3).

**Comment 2**: *Revise the first paragraph of the Introduction for clarity.*

We sincerely appreciate the reviewer's suggestion to revise the first paragraph of the Introduction for greater clarity, as this helps ensure the manuscript is more accessible to readers.

In response, we have revised the first paragraph to make the message clearer by reorganizing the content with better flow, shorter sentences, and explicit transitions.

**Original:** Catheter-related thrombosis (CRT) represents a significant complication in intensive care unit (ICU) patients(1). Recent multicenter studies have reported that central venous catheters are utilized in over 85% of ICU patients, with CRT incidence rates varying considerably from 3.6% to 59%(2). This complication not only prolongs hospital stays and increases healthcare costs but also poses substantial risks for life-threatening complications such as pulmonary embolism(3). Furthermore, CRT has been associated with increased mortality rates and adverse clinical outcomes in critically ill patients(4)

**Revised:** Catheter-related thrombosis (CRT) is a major complication in intensive care unit (ICU) patients, affecting over 85% of those with central venous catheters(1,2). Incidence rates range from 3.6% to 59%, leading to prolonged hospital stays, increased healthcare costs, and severe risks such as pulmonary embolism and higher mortality(3,4). These impacts highlight the urgent need for better prevention strategies

**Comment 3**: *Describe catheter types if available.*

We acknowledge this limitation. The MIMIC-IV database identifies CRT via diagnosis codes but does not specify catheter types (e.g., PICC vs. CVC).

**We have added this as a limitation**: “Fourth, the MIMIC-IV database does not provide granular details on the catheters used, including material type (e.g., polyurethane vs. silicone). Given that these factors are well-established determinants of thrombosis risk, their absence constitutes a significant limitation and may introduce unmeasured heterogeneity into our findings.”

**Comment 4**: *Clarify how thrombosis was diagnosed.*

We appreciate the reviewer’s attention to the details of the diagnostic methods and thank you for emphasizing their importance to the reliability of our study.
We agree with your suggestion and have added further details in the Methods section under “Outcome Measurement,” describing the diagnostic assessment approach based on the MIMIC database. We have expanded the Outcome Measurement section:
**Original**: The primary outcome was the development of catheter-related thrombosis during the ICU stay. Thrombosis was identified through documented diagnosis codes and clinical notes.

**Revised:** The primary outcome was the development of catheter-related thrombosis during the ICU stay. Thrombosis was identified through documented ICD-9/10 diagnosis codes (e.g., 453.81 for acute venous embolism and thrombosis) and clinical notes in the MIMIC-IV database. Diagnosis typically involved complementary examinations such as duplex ultrasound or computed tomography angiography, as per standard protocols at Beth Israel Deaconess Medical Center, though specific per-case details are not uniformly recorded.

**Comment 5**: *Improve Tables 1 and 2.*

we sincerely thank the reviewer for their valuable suggestions regarding the readability and presentation of our tables. we fully understand the reviewer’s concerns: in the original Table 1, the reporting of both "yes" and "no" percentages for binary variables such as vasopressin and anticoagulant use was redundant; and the original layout of Table 2 was overly compact, making it difficult to interpret the association estimates across different adjustment models. In response, we have carefully revised both tables in accordance with the reviewer’s recommendations to enhance clarity and data accessibility.

The specific revisions are as follows:

Table 1: For vasopressin and anticoagulant use, we now report only the proportion of patients who received these interventions (i.e., the “Yes” percentage), removing the redundant “No” category.

Table 2: We have completely reformatted the table to clearly separate the results for continuous and categorical PO₂ analyses, and to align the odds ratios (ORs), 95% confidence intervals (CIs), and *P*-values for the non-adjusted, Model I, and Model II regression models in a vertically structured and reader-friendly format.

**Comment 6**: *Expand the limitations section.*

we sincerely thank the reviewer for their insightful comments regarding the limitations of our study. we fully understand the reviewer’s concerns. Although the MIMIC-IV database is derived from a single-center ICU (Beth Israel Deaconess Medical Center), the retrospective nature of our analysis, the absence of standardized diagnostic protocols for catheter-related thrombosis, and the lack of detailed information on catheter characteristics (such as material type) represent important methodological constraints that warrant explicit acknowledgment.

**In response**, we sincerely thank the reviewer for their insightful comments regarding the limitations of our study.

**Original**: “…unmeasured confounders such as genetic background and specific medication regimens.

**Revised**: This study has several limitations that warrant consideration. First, our analysis is based on the MIMIC-IV database, which is a single-center retrospective cohort. While this provides a large and detailed dataset, the findings may not be fully generalizable to other healthcare settings or populations with different baseline characteristics. Second, as a retrospective study, our analysis is inherently susceptible to residual confounding and selection bias. Although we adjusted for a comprehensive set of clinically relevant covariates, unmeasured or unrecorded confounders (e.g., specific genetic predispositions, detailed medication regimens, or fluid balance) may still influence the observed associations. Third, the diagnosis of catheter-related thrombosis (CRT) relied on ICD coding and supporting clinical documentation, without a standardized, protocol-driven diagnostic approach across the ICU. This may introduce outcome misclassification, potentially leading to either under- or over-estimation of the true CRT incidence. Fourth, the MIMIC-IV database does not provide granular details on the catheters used, including material type (e.g., polyurethane vs. silicone). Given that these factors are well-established determinants of thrombosis risk, their absence constitutes a significant limitation and may introduce unmeasured heterogeneity into our findings. Finally, our data spans from 2008 to 2019, and clinical practices regarding both oxygen therapy and thromboprophylaxis may have evolved since then, which could affect the contemporary applicability of our results.

We believe these revisions have significantly strengthened the manuscript. Thank you again for your valuable feedback.

Sincerely,
Qing Cui, Anshuai Fang, Haiyan Zhang, Qingtong Meng, Sijia Zhou, Yueming Peng, Weixiang Luo
