# Supplementary material for: Association between early post-insertion arterial oxygen tension and documented acute upper-body deep or central venous thrombosis in critically ill adults: A retrospective MIMIC-IV cohort study
Source: PLoS One. 2026 Oct 5;21(10):e0359719. doi: 10.1371/journal.pone.0359719 (PMC13637912; doi:10.1371/journal.pone.0359719)
Supplement: Supplementary file 4 [file pone.0359719.s006.docx]

Response to the Academic Editor and Reviewers

**Manuscript ID: PONE-D-25-27701**

**Revised title:** Association between early post-insertion arterial oxygen tension and documented acute upper-body deep or central venous thrombosis in critically ill adults: a retrospective MIMIC-IV cohort study

# General response

**Dear Academic Editor and Reviewers,**

We sincerely thank you for the careful and constructive evaluation of our manuscript. We agree that the previous analytical framework did not adequately establish a clinically meaningful temporal relationship between oxygen measurement, catheter placement, and the thrombotic outcome, and that the reported 384-mmHg breakpoint was statistically unstable and clinically difficult to interpret.

Rather than retaining the previous framework, we substantially reconstructed the cohort and repeated the analyses. The revised exposure is the first arterial PaO₂ measurement obtained at or after insertion of a qualifying invasive venous catheter, within 24 hours after insertion, during the ICU stay, and while at least one qualifying catheter remained in situ. Because hospitalization-level diagnosis codes lack reliable thrombus-onset timestamps and cannot establish catheter causation, the outcome is no longer described as confirmed catheter-related thrombosis. It is now described as documented acute upper-body deep or central venous thrombosis during hospitalization.

We completely removed the segmented regression analysis, the 384-mmHg threshold, and all claims that higher PaO₂ has a protective effect. The reconstructed cohort included 11,277 hospital admissions from 10,789 patients, with 195 primary outcome events. In the fully adjusted model, PaO₂ was not associated with the outcome (OR per 10-mmHg increase, 1.008; 95% CI, 0.989–1.027; P = 0.430). The revised model includes catheter characteristics, the interval from catheter insertion to PaO₂ measurement, FiO₂, respiratory-support status, and non-respiratory organ dysfunction. Categorical, restricted cubic spline, alternative-outcome, PaO₂/FiO₂, subgroup, complete-case, and extreme-value sensitivity analyses were consistent with the primary result.

We also added the complete ICD code dictionary and outcome-classification rules in Supplementary Table S10, additional timing and upper-tail PaO₂ distribution data in Supplementary Table S11, and a completed STROBE checklist. The title, abstract, Methods, Results, Discussion, Conclusion, tables, figure captions, and Supporting Information have been extensively revised.

For clarity, the reviewer comments are summarized below in bold headings, followed by our responses and the corresponding locations of the revisions.

# Reviewer #1

## Comment 1. Temporal relationship between PaO₂ measurement, catheter placement, catheter dwell time, and thrombosis development

**Reviewer comment:** The temporal relationship remained insufficiently clarified. The reviewer requested detailed timing from ICU admission and catheter insertion to PaO₂ measurement, as well as from PaO₂ measurement to thrombosis diagnosis, and questioned whether a single early value could represent the relevant exposure for a later thrombotic event.

**Response:** We agree that the previous exposure definition was insufficient. We therefore reconstructed the cohort using qualifying catheter insertion as the index time. An eligible PaO₂ measurement was required to occur at or after catheter insertion, within 24 hours after insertion, during the corresponding ICU stay, and while at least one qualifying catheter remained active. The first measurement meeting all four criteria was selected.

We also characterized the timing of arterial blood-gas sampling. In an additional post hoc descriptive analysis, the median interval from ICU admission to the selected PaO₂ measurement was 299 minutes (IQR, 176–525) in admissions without the primary outcome and 494 minutes (IQR, 232–1,160) in admissions with the primary outcome. The corresponding intervals from catheter insertion to PaO₂ measurement were 45 minutes (IQR, 22–152) and 134 minutes (IQR, 60.5–339), respectively. The catheter-insertion-to-PaO₂ interval was included in the adjusted models after log transformation, and subgroup analyses across ≤1 hour, >1–6 hours, and >6–24 hours showed no evidence of interaction (P for interaction = 0.223).

However, the hospitalization-level diagnosis records do not provide a sufficiently reliable time of thrombus onset. We therefore cannot calculate a valid interval from PaO₂ measurement to thrombus formation, confirm that PaO₂ preceded thrombus formation in every admission, or calculate catheter dwell time before thrombus onset. For the same reason, a valid time-to-event or competing-risk analysis could not be performed. We have made these limitations explicit and no longer describe the outcome as confirmed catheter-related thrombosis.

**Changes in the revised submission:** Methods—Exposure assessment and Outcome definitions; Results—Study population and baseline characteristics; Table 1B; Supplementary Table S11; Discussion—limitations.

## Comment 2. The 384-mmHg threshold was clinically uninterpretable and potentially driven by sparse data and model instability

**Reviewer comment:** The reviewer questioned the clinical relevance and statistical stability of the reported 384-mmHg breakpoint and requested information on the upper tail of the PaO₂ distribution and sensitivity analyses excluding extreme values.

**Response:** We agree. The segmented regression analysis, the 384-mmHg breakpoint, and all threshold-based claims have been removed from the title, abstract, Methods, Results, tables, figures, Discussion, and Conclusion.

For transparency, an additional post hoc descriptive summary showed that the reconstructed cohort contained 647 admissions and 10 primary-outcome events with PaO₂ >384 mmHg, 490 admissions and 5 events with PaO₂ >400 mmHg, and 111 admissions and 2 events above the 99th percentile (>461 mmHg). These counts confirm that the upper tail was sparse. Potential nonlinearity is now evaluated using restricted cubic splines, which showed no evidence of an overall association (P = 0.186) or nonlinearity (P = 0.109). Sensitivity analyses excluding PaO₂ >400 mmHg and values above the 99th percentile also yielded null estimates.

**Changes in the revised submission:** Threshold analysis removed throughout; Results—Sensitivity, nonlinear, and subgroup analyses; revised Fig 2; Supplementary Tables S1, S2, and S11.

## Comment 3. Suitability of MIMIC-IV for the research question and need for validation using original clinical data

**Reviewer comment:** The reviewer suggested that, if the database cannot establish a valid exposure window and oxygenation context, the study question may require validation or re-conduction using original clinical data.

**Response:** We agree that MIMIC-IV cannot support a causal or time-to-event analysis of confirmed catheter-related thrombosis because reliable thrombus-onset timestamps, standardized imaging ascertainment, and several detailed catheter variables are unavailable. We therefore narrowed the research question to the association between a clearly defined early post-insertion PaO₂ measurement and a hospitalization-level documented upper-body thrombotic outcome.

Within this narrower scope, MIMIC-IV provides catheter insertion and removal records, arterial blood-gas timing, FiO₂, respiratory-support information, laboratory data, and illness-severity variables that permit a transparent observational analysis. We explicitly acknowledge the remaining temporal, exposure, and outcome-ascertainment limitations and recommend prospective validation using repeated oxygenation measurements, detailed catheter data, and accurately timed, imaging-confirmed thrombotic outcomes.

**Changes in the revised submission:** Title, objective, exposure definition, outcome terminology, Discussion, limitations, and Conclusion.

# Reviewer #3

## Comment 1. The estimate above 384 mmHg was non-interpretable because of complete separation or sparse events

**Reviewer comment:** The reviewer noted that OR = 0.00 with an infinite upper confidence limit indicated a non-estimable result rather than a true plateau.

**Response:** We agree. The previous estimate reflected sparse-data separation and should not have been interpreted as a plateau. The breakpoint model and every reference to 384 mmHg as a threshold have been removed. No threshold result remains in the revised manuscript. Upper-tail counts are reported only as an additional post hoc descriptive summary to demonstrate the sparsity that affected the previous model.

**Changes in the revised submission:** Threshold analysis removed from all manuscript sections, tables, and figures; Supplementary Table S11.

## Comment 2. Report the continuous association per a clinically meaningful increment and temper the interpretation

**Reviewer comment:** The reviewer recommended reporting the OR per 10 mmHg rather than per 1 mmHg and avoiding overstatement of a near-null effect.

**Response:** PaO₂ is now reported per 10-mmHg increase throughout the manuscript. The unadjusted estimate was OR 0.970 (95% CI, 0.952–0.987), but the association attenuated after adjustment. The fully adjusted estimate was OR 1.008 (95% CI, 0.989–1.027; P = 0.430). We therefore no longer describe the result as a significant inverse association, risk reduction, or protective effect.

**Changes in the revised submission:** Abstract; Methods—Statistical analysis; Results; Tables 2 and 3; Discussion; Conclusion.

## Comment 3. Account for FiO₂, ventilation, and the PaO₂/FiO₂ ratio

**Reviewer comment:** The reviewer noted that PaO₂ partly reflects oxygen treatment and recommended adjustment for FiO₂ and ventilation and analysis of the PaO₂/FiO₂ ratio.

**Response:** We agree that PaO₂ cannot be interpreted independently of oxygen delivery and respiratory support. The fully adjusted model now includes FiO₂ at the time of PaO₂ measurement, respiratory-support status, and the non-respiratory component of the first-day SOFA score. Respiratory support was classified as invasive mechanical ventilation, other recorded respiratory support, or no contemporaneous respiratory-support interval identified.

We also analyzed the PaO₂/FiO₂ ratio as an alternative exposure. It was not associated with the outcome (OR per 50-unit increase, 1.010; 95% CI, 0.957–1.065; P = 0.730). A high-reliability FiO₂ sensitivity analysis restricted FiO₂ to the same blood-gas record or a ventilator setting recorded within 60 minutes of PaO₂ measurement and also yielded a null result.

**Changes in the revised submission:** Methods—Covariates and Statistical analysis; Results—Sensitivity analyses; Supplementary Tables S1–S3.

## Comment 4. Outcome underascertainment and differential detection bias

**Reviewer comment:** The reviewer requested the total number of thrombotic events and discussion of possible underascertainment and differential imaging or documentation.

**Response:** We agree. The revised primary outcome occurred in 195 of 11,277 admissions (1.73%): 32 events in the PaO₂ ≤80-mmHg group, 29 in the 81–100-mmHg group, and 134 in the >100-mmHg group. We removed the term confirmed catheter-related thrombosis. The primary outcome is now restricted to documented acute upper-body deep or central venous thrombosis, whereas superficial and anatomically unspecified diagnoses are excluded.

A broader outcome included 282 events, and a strict laterality-compatible deep or central venous outcome included 69 events; both yielded null adjusted PaO₂ estimates. For the strict definition, a right-sided diagnosis required a right-sided qualifying catheter, a left-sided diagnosis required a left-sided qualifying catheter, and a bilateral diagnosis was considered compatible; diagnoses with unspecified laterality were excluded. We now explicitly acknowledge that asymptomatic or clinically unsuspected events may have been missed and that outcome detection may have varied according to diagnostic testing and documentation. If patients with greater illness severity or lower PaO₂ were imaged more frequently, differential ascertainment could have contributed to the apparent inverse association in the less-adjusted models.

**Changes in the revised submission:** Methods—Outcome definitions; Results—event counts and alternative outcomes; Table 1A; Supplementary Tables S3 and S10; Discussion—limitations.

## Comment 5. A single PaO₂ value cannot capture dynamic exposure; a time-varying model would be preferable

**Reviewer comment:** The reviewer emphasized that one baseline value does not represent oxygenation over the ICU stay and raised concern about differing exposure windows and time-related bias.

**Response:** We agree that a single early PaO₂ measurement cannot represent cumulative or time-varying oxygen exposure. The revised research question is explicitly limited to the association of one early post-insertion measurement with a hospitalization-level documented outcome. We do not interpret the selected value as a measure of cumulative hypoxemia or hyperoxemia.

A time-varying Cox or competing-risk model could not be implemented validly because reliable thrombus-onset timestamps were unavailable. The revised Discussion explicitly acknowledges the single-measurement limitation, temporal uncertainty, selection related to requiring an eligible arterial blood-gas measurement, and the inability to perform valid time-to-event analyses. Future studies should use repeated or time-weighted oxygenation measurements and accurately timed, imaging-confirmed outcomes.

**Changes in the revised submission:** Methods—Exposure assessment; Discussion—limitations; Conclusion.

## Comment 6. Improve STROBE reporting, including missing data, event counts, and model performance

**Reviewer comment:** The reviewer requested the number excluded for missing variables, comparison of missing cases, event counts by PaO₂ group, and calibration/discrimination information.

**Response:** Covariate missingness is now handled using multiple imputation rather than excluding admissions solely for missing prespecified covariates. Supplementary Table S6 reports observed and missing counts, missingness percentages, imputation methods, and imputation diagnostics. A complete-case sensitivity analysis included 8,906 admissions and 139 events.

Event counts by PaO₂ category are reported in the Results and Table 1A. Supplementary Table S5 reports the mean apparent AUC (0.7834), mean apparent Brier score (0.0166), generalized variance inflation factors, leverage, Cook’s distance, DFBETA, and an influence analysis. Supplementary Figure S2 presents apparent calibration and is explicitly described as uncorrected for optimism and not as internal or external validation. A completed STROBE checklist is submitted separately.

**Changes in the revised submission:** Fig 1; Results; Tables 1A–1B; Supplementary Tables S5–S6; Supplementary Figure S2; S1 Checklist.

## Comment 7. The biological rationale relied too heavily on COVID-19 cohorts

**Reviewer comment:** The reviewer requested acknowledgment that COVID-19-associated coagulopathy may not generalize to an unselected ICU population and recommended broader evidence.

**Response:** We agree. The biological rationale and Discussion were rewritten and no longer rely on COVID-19 cohorts. The revised text considers intermittent hypoxia in obstructive sleep apnea, environmental hypoxia, COPD-associated thromboembolism, perioperative hyperoxia, and randomized ICU oxygen-target trials. We also emphasize that these settings cannot be directly extrapolated to a single routine ICU PaO₂ measurement.

**Changes in the revised submission:** Introduction and Discussion.

## Comment 8. Table 1 contained misaligned or internally inconsistent cells

**Reviewer comment:** The reviewer requested verification of values, units, and decimal places and improvement of table alignment.

**Response:** Table 1 was reformatted, and the values, denominators, units, and decimal places were checked. To improve readability, the three pairwise standardized mean-difference columns were removed from the main Table 1A and transferred to Supplementary Table S9.

**Changes in the revised submission:** Reformatted Tables 1A–1B; new Supplementary Table S9.

## Comment 9. Standardize PO₂ and PaO₂ notation

**Reviewer comment:** The reviewer requested consistent use of PaO₂ for arterial oxygen tension.

**Response:** The terminology has been standardized to PaO₂ throughout the title, abstract, main text, tables, figure captions, and supporting information.

**Changes in the revised submission:** All manuscript and supporting-information files.

## Comment 10. Figure 2 should report the sample size and show the PaO₂ distribution

**Reviewer comment:** The reviewer requested the number of observations and a visual indication of the exposure distribution because of wide confidence intervals at the extremes.

**Response:** The revised restricted cubic spline figure includes rug marks showing the observed PaO₂ distribution and displays the 95% confidence interval across the plotted range. The caption now states that the analysis included 11,277 hospital admissions and 195 outcome events. Supplementary Table S11 also reports the overall PaO₂ distribution and upper-tail counts.

**Changes in the revised submission:** Revised Fig 2 and caption; Supplementary Table S11.

# Reviewer #4

## Major Comment 1. Fundamental concern regarding outcome ascertainment

**Reviewer comment:** The reviewer requested the exact ICD codes, validation strategy, sensitivity and specificity, anatomical restriction, and an explanation of how unrelated DVTs were excluded.

**Response:** We agree that the previous terminology overstated what could be established from the available data. The revised outcome is not described as confirmed catheter-related thrombosis. It is now defined as documented acute upper-body deep or central venous thrombosis during hospitalization. The revised algorithm uses the MIMIC-IV diagnoses_icd table and no longer relies on clinical notes.

The primary definition includes acute ICD-9-CM and ICD-10-CM codes for deep upper-extremity, axillary, subclavian, and internal jugular venous thrombosis. Superficial, chronic, and anatomically unspecified diagnoses are excluded from the primary definition. A broader acute outcome and a strict laterality-compatible deep or central venous outcome were evaluated as sensitivity analyses. For the strict definition, right-sided diagnoses were required to match a right-sided qualifying catheter, left-sided diagnoses were required to match a left-sided qualifying catheter, bilateral diagnoses were considered compatible, and diagnoses with unspecified laterality were excluded. The complete code list and classification rules are provided in Supplementary Table S10.

No chart-adjudicated or imaging-adjudicated reference standard was available; therefore, we cannot estimate or claim the sensitivity or specificity of the coding algorithm. We also cannot exclude every non-catheter-related upper-body thrombosis or establish catheter causation. These limitations are now stated explicitly.

**Changes in the revised submission:** Methods—Outcome definitions; Supplementary Tables S3 and S10; Discussion—limitations.

## Major Comment 2. The exposure definition based on a single first PaO₂ measurement was problematic

**Reviewer comment:** The reviewer asked how soon PaO₂ was measured, whether thrombosis may already have been present, and why time-weighted, maximum, minimum, or cumulative measures were not used.

**Response:** The exposure is no longer the first PaO₂ recorded during the ICU stay. It is the first PaO₂ obtained at or after qualifying catheter insertion, within 24 hours, during the corresponding ICU stay, and while the catheter remained active. This definition provides a reproducible early post-insertion measurement and avoids using later measurements that may be increasingly affected by the subsequent ICU course.

We agree that the selected value remains a single measurement and cannot represent time-weighted, maximum, minimum, or cumulative oxygen exposure. Reliable thrombus-onset timestamps were unavailable, so repeated oxygen measurements could not be aligned with event onset, and pre-existing thrombosis at the time of the selected measurement cannot be excluded in every admission. These limitations are now stated explicitly. The timing from ICU admission and catheter insertion to the selected PaO₂ is reported in the Results and Supplementary Table S11.

**Changes in the revised submission:** Methods—Exposure assessment and Outcome definitions; Results—timing description; Supplementary Table S11; Discussion—limitations.

## Major Comment 3. Temporal relationship remained unclear; Cox or competing-risk analysis was suggested

**Reviewer comment:** The reviewer requested catheter insertion time, PaO₂ time, and thrombosis time and suggested time-to-event methods.

**Response:** Exact catheter insertion and selected PaO₂ times were available and are now linked explicitly. The interval from catheter insertion to PaO₂ was incorporated into the adjusted models, and timing by outcome status is reported. However, thrombosis was available only as a hospitalization-level diagnosis without a sufficiently reliable onset time. Using a diagnosis-code date as biological onset would create false precision.

We therefore retained hospitalization-level logistic regression, clearly limited the estimand to an association with a documented hospitalization-level outcome, and removed claims that PaO₂ preceded thrombus formation. We now state explicitly that valid Cox, time-to-event, and competing-risk analyses could not be performed.

**Changes in the revised submission:** Methods—Exposure assessment and Outcome definitions; Results; Supplementary Table S11; Discussion—limitations.

## Major Comment 4. Severe confounding by illness severity; propensity-score or weighting methods were recommended

**Reviewer comment:** The reviewer noted major baseline differences and recommended propensity-score matching, IPTW, or doubly robust analyses.

**Response:** We agree that illness severity and treatment context are important sources of confounding. The revised fully adjusted model includes age, sex, Charlson Comorbidity Index, hemoglobin, platelet count, lactate, catheter type, catheter laterality, multiple active catheters, the interval from catheter insertion to PaO₂ measurement, FiO₂, respiratory-support status, and the non-respiratory component of the first-day SOFA score. The attenuation of the inverse estimate after catheter, timing, oxygenation, and severity variables were added is reported transparently.

We considered the suggested propensity-score methods. However, PaO₂ is a continuous physiologic measurement rather than an assigned binary treatment. Conventional matching or binary-treatment IPTW would require an arbitrary PaO₂ cutoff, discard exposure information, and change the estimand. These approaches would also not resolve unavailable thrombus-onset times or unmeasured confounding. We therefore retained prespecified continuous and categorical analyses with sequential multivariable adjustment, multiple imputation, patient-clustered robust standard errors, nonlinear analysis, and extensive sensitivity analyses. The manuscript consistently interprets all estimates as associations rather than causal effects.

**Changes in the revised submission:** Methods—Covariates and Statistical analysis; Results—sequential and module-specific models; Discussion—interpretation and limitations.

## Major Comment 5. Potential reverse causality

**Reviewer comment:** The reviewer suggested that illness severity could cause both hypoxemia and thrombosis rather than PaO₂ causing thrombosis and requested more cautious interpretation.

**Response:** We agree. The revised models account for available measures of illness severity, respiratory treatment, laboratory status, catheter characteristics, and measurement timing. The inverse estimate observed in the unadjusted and minimally adjusted models moved to the null after these variables were included. We now interpret this attenuation as evidence that the crude association was sensitive to clinical and treatment context, not as evidence of an independent or causal effect of PaO₂.

Because thrombus-onset time was unavailable, reverse temporal ordering cannot be excluded. This limitation is stated explicitly, and all causal terminology has been removed.

**Changes in the revised submission:** Results—sequential and module-specific analyses; Discussion—interpretation and limitations; Conclusion.

## Major Comment 6. The threshold analysis was statistically fragile

**Reviewer comment:** The reviewer questioned the biological plausibility and statistical stability of the 384-mmHg threshold, noting the likely sparsity of observations in the upper tail and the non-estimable estimate above the breakpoint.

**Response:** We agree. The segmented regression analysis, the 384-mmHg breakpoint, and all threshold-based claims have been removed from the title, abstract, Methods, Results, tables, figures, Discussion, and Conclusion. In an additional post hoc descriptive summary, 647 admissions with 10 primary-outcome events had PaO₂ values greater than 384 mmHg, 490 admissions with 5 events had values greater than 400 mmHg, and 111 admissions with 2 events had values above the 99th percentile (>461 mmHg). These counts confirmed the sparsity of the upper tail. Potential nonlinearity is now evaluated using restricted cubic splines, which showed no evidence of an overall association (P = 0.186) or nonlinearity (P = 0.109). Sensitivity analyses excluding PaO₂ values greater than 400 mmHg and values above the 99th percentile also yielded null estimates.

**Changes in the revised submission:** Threshold analysis removed throughout; Results—Sensitivity, nonlinear, and subgroup analyses; Fig 2; Supplementary Tables S1, S2, and S11.

## Major Comment 7. Inconsistency between continuous and categorical analyses

**Reviewer comment:** The reviewer noted discordance between a near-null continuous estimate and substantially lower categorical odds.

**Response:** The discrepancy is no longer present after cohort reconstruction and comprehensive adjustment. In the fully adjusted model, the continuous estimate was OR 1.008 (95% CI, 0.989–1.027) per 10-mmHg increase. The categorical estimates were OR 0.900 (95% CI, 0.531–1.526) for 81–100 versus ≤80 mmHg and OR 0.989 (95% CI, 0.652–1.502) for >100 versus ≤80 mmHg. Restricted cubic spline analysis also showed no overall or nonlinear association.

**Changes in the revised submission:** Results; Tables 2–3; Fig 2.

## Major Comment 8. Anticoagulation may introduce overadjustment or collider bias

**Reviewer comment:** The reviewer questioned adjustment for anticoagulant administration because its timing and indication may be related to perceived thrombotic risk.

**Response:** We agree that time-varying anticoagulation is difficult to position analytically when indication and timing relative to exposure and outcome are uncertain. Anticoagulant administration is not included in the revised primary adjustment model. The Discussion now acknowledges that detailed time-varying information on thromboprophylaxis and therapeutic anticoagulation was limited, leaving potential residual confounding. We did not make causal claims about the role of anticoagulation.

**Changes in the revised submission:** Methods—Covariate specification; Discussion—limitations.

## Major Comment 9. Important catheter and clinical risk factors were missing

**Reviewer comment:** The reviewer listed catheter type, lumens, size, dwell time, tip position, ventilation, cancer, sepsis, platelet count, D-dimer, and coagulation variables.

**Response:** The revised analysis incorporates catheter type, catheter laterality, multiple active qualifying catheters, platelet count, hemoglobin, lactate, FiO₂, respiratory-support status, Charlson Comorbidity Index, and non-respiratory SOFA. PICC status was also evaluated in subgroup analysis.

Nevertheless, catheter material, exact diameter, catheter-to-vein ratio, exact tip position, insertion technique, operator-related factors, complete dwell time before thrombus onset, D-dimer, detailed coagulation measures, and detailed time-varying anticoagulation were unavailable or could not be measured reliably. These limitations are now stated explicitly. The associations of individual covariates are presented as secondary, hypothesis-generating findings and are not described as independent risk factors.

**Changes in the revised submission:** Methods—Covariates; Results—secondary covariate associations; Supplementary Table S8; Discussion—limitations.

## Major Comment 10. Causal wording such as “protective effect” and “risk reduction” should be removed

**Reviewer comment:** The reviewer requested consistent noncausal terminology.

**Response:** We agree. Terms including protective effect, risk reduction, and higher PaO₂ reduces thrombosis have been removed. The revised manuscript consistently uses association, odds, and documented outcome. The Conclusion states that the findings do not support PaO₂-based thrombosis risk stratification or modification of oxygen therapy for thrombosis prevention.

**Changes in the revised submission:** Title; Abstract; Results; Discussion; Conclusion.

## Minor Comment 1. Use one study-design term consistently

**Reviewer comment:** The reviewer noted inconsistent use of retrospective cohort and cross-sectional terminology.

**Response:** The study is now consistently described as a retrospective observational cohort study. Cross-sectional terminology has been removed.

**Changes in the revised submission:** Title, Abstract, Methods, and Discussion.

## Minor Comment 2. Standardize PO₂ and PaO₂

**Reviewer comment:** The reviewer requested consistent arterial oxygen terminology.

**Response:** PaO₂ is now used consistently throughout the manuscript and supporting information.

**Changes in the revised submission:** All files.

## Minor Comment 3. Clarify APSIII versus APACHE III

**Reviewer comment:** The reviewer requested clarification regarding the severity score used in the models.

**Response:** Neither APSIII nor APACHE III is included in the revised primary models. Illness severity is represented by the non-respiratory component of the first-day SOFA score, which is consistently defined and reported.

**Changes in the revised submission:** Methods—Covariates and Statistical analysis; tables and supporting information.

## Minor Comment 4. Report continuous ORs per a meaningful increment

**Reviewer comment:** The reviewer recommended reporting the continuous association per 10 mmHg or per standard deviation.

**Response:** All continuous PaO₂ associations are now reported per 10-mmHg increase.

**Changes in the revised submission:** Abstract; Methods; Results; tables; figures; supporting information.

## Minor Comment 5. Report handling of missing data

**Reviewer comment:** The reviewer requested the number and percentages of missing variables and consideration of multiple imputation.

**Response:** Missing values in prespecified model covariates were handled using multiple imputation by chained equations with 20 imputed datasets. Supplementary Table S6 reports observed and missing counts, missingness percentages, imputation methods, and diagnostics. Supplementary Figures S3 and S4 provide graphical imputation diagnostics. A complete-case sensitivity analysis was also performed.

**Changes in the revised submission:** Methods—Statistical analysis; Supplementary Table S6; Supplementary Figures S3–S4.

## Minor Comment 6. Provide the MIMIC-IV version and reproducible extraction and analysis code

**Reviewer comment:** The reviewer requested the exact database version, SQL extraction strategy, and a reproducible code repository or package.

**Response:** Thank you for this important suggestion. The database version is now specified as MIMIC-IV v2.2, and the Methods and Supporting Information have been expanded to describe the cohort-construction logic, eligibility criteria, exposure window, outcome definitions, ICD code list, covariate definitions, missing-data procedures, model specifications, and sensitivity analyses. We agree that sharing well-documented analysis code would further improve reproducibility. The reported results were generated using the final analytic workflow; however, the relevant code currently remains distributed across several working scripts and has not yet been consolidated and documented as a single publication-ready repository. To avoid providing an incomplete or potentially misleading package, we have not included executable code with this revision. If the Editor considers code submission essential for further evaluation, we would be grateful for the opportunity to prepare and provide a fully consolidated and documented package. Patient-level MIMIC-IV data are not redistributed because they are subject to the PhysioNet Credentialed Health Data Use Agreement; qualified researchers may obtain the data through the same credentialing process, and the authors had no special access privileges.

**Changes in the revised submission:** Methods—Data source and Statistical analysis; Supporting Information; Data Availability Statement in the submission system.

# Closing statement

We again thank the Academic Editor and Reviewers for their detailed and constructive comments. The review prompted a fundamental reconstruction of the study, removal of the unstable threshold claim, more complete adjustment for catheter and oxygenation context, and a substantially more cautious interpretation. We believe that the revised manuscript now addresses the concerns transparently and reports the null fully adjusted result without causal or threshold-based claims.

**Sincerely,**

Qing Cui, on behalf of all authors
