# Supplementary material for: Association between early post-insertion arterial oxygen tension and documented acute upper-body deep or central venous thrombosis in critically ill adults: A retrospective MIMIC-IV cohort study
Source: PLoS One. 2026 Oct 5;21(10):e0359719. doi: 10.1371/journal.pone.0359719 (PMC13637912; doi:10.1371/journal.pone.0359719)
Supplement: Supplementary file 5 [file pone.0359719.s007.docx]

Response to Reviewers

**Manuscript ID: PONE-D-25-27701R2**

**Revised title:** Association between early post-insertion arterial oxygen tension and documented acute upper-body deep or central venous thrombosis in critically ill adults: a retrospective MIMIC-IV cohort study

# General response

Dear Academic Editor and Reviewers,

We sincerely thank the Academic Editor and Reviewers for the careful and constructive reassessment of our manuscript. We appreciate the recognition that the reconstructed cohort, revised outcome definition, removal of the previous threshold-based interpretation, expanded adjustment strategy, and sensitivity analyses have substantially improved the study.

In this revision, we focused on the remaining issues concerning measurement timing, the role of FiO₂ in the adjustment strategy, study-design terminology and temporal ordering, and outcome ascertainment. We performed an additional narrow-window sensitivity analysis restricted to PaO₂ measurements obtained within 60 minutes after catheter insertion. This analysis included 6,301 admissions from 6,185 patients and 49 primary outcome events. Model 2 was not statistically significant (OR per 10-mmHg increase, 1.026; 95% CI, 0.995–1.058; P = 0.102), whereas Model 3 showed a positive association (OR, 1.041; 95% CI, 1.010–1.073; P = 0.010). We therefore revised the manuscript to report the result transparently as adjustment-set dependent rather than describing all sensitivity analyses as uniformly consistent with the primary null result.

We also clarified that the interpretation of FiO₂ adjustment depends on the estimand, while noting that the complete-cohort Model 2 was already null before inclusion of FiO₂, respiratory-support status, and non-respiratory SOFA. We retained PaO₂/FiO₂ as an alternative exposure rather than an adjustment variable. In addition, we clarified that the study evaluates admission-level associations rather than time-to-event relationships, explicitly acknowledged that the selected PaO₂ measurement could not be confirmed to precede thrombus formation in every admission, and added a statement that the observed event rate may be lower than rates reported in studies using systematic imaging surveillance.

The Abstract, Methods, Results, Discussion, Conclusion, and Supporting Information have been revised accordingly. Supplementary Table S12 has been added to report the 60-minute restricted-cohort analysis. For clarity, each comment is addressed point by point below.

# Academic Editor

## Comment 1. Timing between catheter insertion and PaO₂ measurement

**Editor comment:** The interval between catheter insertion and the selected PaO₂ measurement differs substantially according to outcome status (median 45 vs 134 minutes), and the timing variable itself is associated with the documented thrombotic outcome. The Editor requested an additional sensitivity analysis restricted to a narrow early post-insertion measurement window, preferably within 1 hour, with reporting of the included admissions, events, and adjusted PaO₂ estimate. The related limitation should be described in terms of temporal uncertainty and possible selection related to measurement timing, without implying that a conventional immortal-time bias can be definitively established.

**Response:** We agree. We performed an additional narrow-window sensitivity analysis restricted to admissions in which the selected PaO₂ measurement was obtained within 60 minutes after catheter insertion. This restricted cohort included 6,301 admissions from 6,185 patients, with 49 primary outcome events. In Model 2, the association was not statistically significant (OR per 10-mmHg increase, 1.026; 95% CI, 0.995–1.058; P = 0.102). In the fully adjusted Model 3, the estimate was positive and statistically significant (OR, 1.041; 95% CI, 1.010–1.073; P = 0.010). Both estimates are now reported in the manuscript and in Supplementary Table S12.

Because the restricted cohort contained only 49 events and the estimate differed according to the adjustment set, we interpret this analysis cautiously and describe it as a secondary, hypothesis-generating finding. We also revised the Discussion and Limitations to note that restriction to very early blood-gas measurements may select patients with different illness severity, monitoring intensity, or clinical trajectories. Because reliable thrombus-onset timestamps are unavailable, we do not characterize this issue as definitively representing conventional immortal-time bias; instead, we describe temporal uncertainty and possible selection related to measurement timing.

**Changes in the revised submission:** Abstract—Results and Conclusion; Methods—Statistical analysis; Results—Sensitivity, nonlinear, and subgroup analyses; Discussion—interpretation and limitations; Conclusion; Supplementary Table S12.

## Comment 2. Role of FiO₂ in the adjustment strategy

**Editor comment:** The Reviewer raised the possibility that adjustment for FiO₂ could contribute to over-adjustment because FiO₂ is a determinant of measured PaO₂. The Editor noted that Model 2 already excludes FiO₂, respiratory-support status, and non-respiratory SOFA and is null (OR 1.003, 95% CI 0.984–1.022; P = 0.774), so no additional complete-cohort model is necessary. The manuscript should acknowledge that the interpretation of FiO₂ adjustment depends on the estimand, and PaO₂/FiO₂ should remain an alternative-exposure analysis rather than an adjustment variable.

**Response:** We agree. We now state explicitly that the role of FiO₂ adjustment depends on the estimand because FiO₂ is a direct determinant of measured PaO₂. Importantly, the complete-cohort Model 2, which does not include FiO₂, respiratory-support status, or non-respiratory SOFA, was already null (OR per 10-mmHg increase, 1.003; 95% CI, 0.984–1.022; P = 0.774). Therefore, the primary null finding cannot be attributed solely to adjustment for FiO₂, and we did not add another complete-cohort model.

We continue to present the PaO₂/FiO₂ ratio as an alternative exposure in sensitivity analysis rather than as a covariate in the primary PaO₂ models. For the newly requested 60-minute restricted-cohort analysis, we report both Model 2 and Model 3 because their estimates differed across adjustment sets.

**Changes in the revised submission:** Discussion—interpretation of FiO₂ adjustment; Results and Supplementary Table S12—restricted-cohort Model 2 and Model 3 estimates; PaO₂/FiO₂ retained as an alternative-exposure analysis.

## Comment 3. Study-design terminology and temporal ordering

**Editor comment:** The manuscript is described as a retrospective observational cohort study, but the exact onset of thrombosis cannot be established and the selected PaO₂ measurement cannot be confirmed to precede thrombus formation in every admission. The Editor requested further clarification of this temporal limitation and indicated that the current terminology may be retained if adequately qualified.

**Response:** We agree and have retained the term “retrospective observational cohort study” while qualifying its interpretation more explicitly. The Methods now state that the study evaluates admission-level associations and was not designed as a time-to-event analysis. We also state that, because thrombosis diagnoses lack reliable onset timestamps, temporal precedence of the selected PaO₂ measurement cannot be confirmed for every admission. The Outcome definitions and Limitations reiterate that exact thrombus onset could not be determined and that the selected PaO₂ value could not be confirmed to precede thrombus formation in all admissions.

**Changes in the revised submission:** Methods—Study design and objective and Outcome definitions; Discussion—limitations.

## Comment 4. Outcome ascertainment

**Editor comment:** The manuscript appropriately acknowledges the limitations of ICD-based outcome ascertainment, including lack of standardized prospective imaging, unknown sensitivity and specificity, possible under-detection of asymptomatic events, and differential ascertainment. The Editor requested an explicit statement that the observed event rate may therefore be lower than rates reported in studies using systematic imaging surveillance.

**Response:** We agree and added the requested statement. The Limitations now explicitly note that, because outcome ascertainment relied on hospitalization-level diagnostic records rather than standardized prospective imaging, asymptomatic or clinically unsuspected thromboses may have been missed and the observed event rate may therefore be lower than rates reported in studies using systematic imaging surveillance. We continue to emphasize that the recorded outcome represents documented acute upper-body deep or central venous thrombosis during hospitalization rather than imaging-confirmed catheter-related thrombosis.

**Changes in the revised submission:** Discussion—limitations.

# Reviewer #3

## Major Comment 1. Timing and possible selection/temporal bias

**Reviewer comment:** The interval between catheter insertion and the first PaO₂ measurement differs systematically between patients with and without the outcome and is itself associated with the outcome. The Reviewer requested explicit discussion of the resulting temporal/selection concern and a sensitivity analysis restricting the cohort to a narrow insertion-to-measurement window.

**Response:** We agree and performed the requested restricted-cohort analysis rather than relying only on adjustment for measurement timing or the previous interaction analysis. Among 6,301 admissions from 6,185 patients with PaO₂ measured within 60 minutes after catheter insertion, 49 primary outcome events occurred. Model 2 was not statistically significant (OR per 10-mmHg increase, 1.026; 95% CI, 0.995–1.058; P = 0.102), whereas Model 3 showed a positive association (OR, 1.041; 95% CI, 1.010–1.073; P = 0.010).

We revised the Abstract, Results, Discussion, and Conclusion so that the new restricted analysis is not described as uniformly supporting the primary null result. We also explain that the restricted estimate should be interpreted cautiously because only 49 events remained, the estimate was sensitive to the adjustment set, and selection into very early arterial blood-gas measurement may be related to clinical severity and monitoring intensity. Because thrombus-onset timestamps are unavailable, we describe this issue as temporal uncertainty and possible selection related to measurement timing rather than asserting a definitive conventional immortal-time bias.

**Changes in the revised submission:** Abstract; Methods—Statistical analysis; Results—Sensitivity, nonlinear, and subgroup analyses; Discussion—interpretation and limitations; Conclusion; Supplementary Table S12.

## Major Comment 2. FiO₂ and the PaO₂/FiO₂ ratio

**Reviewer comment:** The Reviewer noted that FiO₂ is a determinant of PaO₂ rather than a classical confounder and raised concern that adjustment could attenuate an association toward the null. The Reviewer requested transparency regarding whether the null result persists without conditioning on FiO₂ and clarification of the role of PaO₂/FiO₂.

**Response:** We agree that FiO₂ is a determinant of measured PaO₂ and that whether it belongs in the adjustment set depends on the estimand. As highlighted by the Academic Editor, the complete-cohort Model 2 excludes FiO₂, respiratory-support status, and non-respiratory SOFA and was already null (OR per 10-mmHg increase, 1.003; 95% CI, 0.984–1.022; P = 0.774). We now make this point explicit in the Discussion. Accordingly, we did not add another complete-cohort model solely to remove FiO₂.

The PaO₂/FiO₂ ratio remains an alternative-exposure analysis and is not included alongside PaO₂ as an adjustment variable in the primary models. We also report Model 2 and Model 3 separately in the new 60-minute restricted-cohort analysis because their estimates differ.

**Changes in the revised submission:** Discussion—role of FiO₂ adjustment; Results and Supplementary Table S12—restricted-cohort estimates; PaO₂/FiO₂ retained as an alternative exposure.

## Minor Comment 3. Study-design terminology and temporal relationship

**Reviewer comment:** The Reviewer noted that a single-time-point exposure and uncertain temporal ordering make the “cohort study” label potentially too strong and requested either softer terminology or explicit clarification in the Methods and Limitations.

**Response:** We agree with the need for qualification. We retained “retrospective observational cohort study” but now explicitly state that the analysis evaluates admission-level associations and was not designed as a time-to-event analysis. The Methods, Outcome definitions, and Limitations clarify that reliable thrombus-onset timestamps were unavailable and that the selected PaO₂ measurement could not be confirmed to precede thrombus formation in every admission.

**Changes in the revised submission:** Methods—Study design and objective and Outcome definitions; Discussion—limitations.

## Minor Comment 4. ICD-based outcome ascertainment and event rate

**Reviewer comment:** The Reviewer noted that administrative ICD coding may under-capture asymptomatic thrombosis and requested a plain statement that residual outcome misclassification remains possible and that the observed event rate is lower than rates reported with systematic screening.

**Response:** We agree. The Limitations now explicitly state that asymptomatic or clinically unsuspected thromboses may have been missed and that the observed event rate may therefore be lower than rates reported in studies using systematic imaging surveillance. We also retain the existing discussion of unknown coding sensitivity and specificity, differential outcome detection, and the absence of chart- or imaging-adjudicated confirmation.

**Changes in the revised submission:** Discussion—limitations.

# Reviewer #4

**Reviewer comment:** All comments have been addressed.

**Response:** We sincerely thank Reviewer #4 for confirming that the previous comments have been addressed. No additional manuscript changes were required specifically in response to Reviewer #4 in this round.

# Closing statement

We again thank the Academic Editor and Reviewers for their careful and constructive guidance. The additional 60-minute restricted-cohort analysis and the requested clarifications have helped us present the temporal limitations, adjustment strategy, and outcome ascertainment more transparently. We have reported the adjustment-dependent restricted-cohort estimates without overstating their implications and have retained the complete-cohort analysis as the primary analysis. We believe that the revised manuscript now addresses all remaining concerns in a balanced and transparent manner.

**Sincerely,**

Qing Cui, on behalf of all authors
