# Supplementary material for: Association between early post-insertion arterial oxygen tension and documented acute upper-body deep or central venous thrombosis in critically ill adults: A retrospective MIMIC-IV cohort study
Source: PLoS One. 2026 Oct 5;21(10):e0359719. doi: 10.1371/journal.pone.0359719 (PMC13637912; doi:10.1371/journal.pone.0359719)
Supplement: Supplementary file 6 [file pone.0359719.s005.DOC]

Dear Editor and Authors,

Thank you for the opportunity to review this manuscript entitled "Association between early post-insertion arterial oxygen tension and documented acute upper-body deep or central venous thrombosis in critically ill adults: a retrospective MIMIC-IV cohort study" (Manuscript ID: PONE-D-25-27701). This is the third review of the manuscript. I am grateful to the authors for the substantial work invested in this revision.

In this study the authors use the MIMIC-IV database to examine whether early post-insertion arterial oxygen tension (PaO2) is associated with documented acute upper-body deep or central venous thrombosis in critically ill adults. Compared with the previous versions, the cohort has been entirely reconstructed (11,277 admissions, 10,789 patients, 195 events, 1.73%), the previously reported 384-mmHg threshold and all "protective" claims have been removed, and the analysis now yields a well-estimated null association (fully adjusted OR 1.008 per 10 mmHg, 95% CI 0.989-1.027, P=0.43), supported by categorical models, a PaO2/FiO2 analysis, restricted cubic splines, multiple imputation, and model-performance and influence diagnostics. I commend the authors; this revision resolves the complete-separation problem and the over-interpretation that affected the original submission, and the manuscript is now scientifically sound and, for a null finding, clearly reportable.

A few points should still be addressed to strengthen the validity and transparency of the work before publication.

**MAJOR REVISIONS**

1. The interval between catheter insertion and the first PaO2 measurement differs systematically between patients with and without the outcome (approximately 134 vs 45 minutes) and is itself a strong predictor in the models (OR 1.33 per unit, P<0.001). This raises a concern of immortal-time-like/selection bias that adjustment alone may not fully remove, because patients who develop the outcome have a different temporal profile. The authors should discuss this explicitly and provide a sensitivity analysis restricting the cohort to a narrow, homogeneous insertion-to-measurement window (e.g., excluding extreme intervals) to show that the null result is robust to this design feature.

2. FiO2 (and the PaO2/FiO2 ratio) is a determinant of the exposure rather than a classical confounder. Adjusting for it may attenuate any true association toward the null through over-adjustment effects. Because the primary conclusion is a null association, this deserves an explicit acknowledgment, and the authors should present a model that excludes FiO2 and the PaO2/FiO2 term, so that readers can judge whether the null persists without conditioning on a determinant of the exposure.

**MINOR REVISIONS**

3. Given that the exposure is captured at a single time point and that the design cannot establish that exposure precedes the outcome, the label "cohort study" is somewhat generous. The authors should either soften this terminology and add a brief statement in the Methods/Limitations clarifying the temporal relationship between exposure measurement and outcome ascertainment.

4. Outcome ascertainment relies on administrative ICD coding, which is known to under-capture asymptomatic thrombosis. The authors have honestly disclosed this and added broad and strict outcome sensitivity analyses, both null. I would only ask that the Limitations section state plainly that residual outcome misclassification remains possible and that the observed event rate is lower than rates reported with systematic screening.

In summary, the authors have thoroughly and honestly addressed the concerns raised in the previous rounds; the reconstructed analysis is rigorous and the null finding is appropriately presented. The remaining requests concern the discussion of temporal/selection bias, transparency around adjustment for a determinant of the exposure, and minor clarifications of terminology and outcome ascertainment. I believe the manuscript can be accepted after these points are addressed.

Best regards,

The reviewer
