# Supplementary material for: Association between early post-insertion arterial oxygen tension and documented acute upper-body deep or central venous thrombosis in critically ill adults: A retrospective MIMIC-IV cohort study
Source: PLoS One. 2026 Oct 5;21(10):e0359719. doi: 10.1371/journal.pone.0359719 (PMC13637912; doi:10.1371/journal.pone.0359719)
Supplement: Peer review history

# Peer review: Decision Letter 0

**DOI:** 10.1371/journal.pone.0359719.r001

**Authors:** Paavani Atluri

6 Nov 2025

Dear Dr. Cui,

Thank you for submitting your manuscript to PLOS ONE. After careful consideration, we feel that it has merit but does not fully meet PLOS ONE’s publication criteria as it currently stands. Therefore, we invite you to submit a revised version of the manuscript that addresses the points raised during the review process.

Please submit your revised manuscript by Dec 20 2025 11:59PM. If you will need more time than this to complete your revisions, please reply to this message or contact the journal office at plosone@plos.org. When you're ready to submit your revision, log on to https://www.editorialmanager.com/pone/ and select the 'Submissions Needing Revision' folder to locate your manuscript file.

- A rebuttal letter that responds to each point raised by the academic editor and reviewer(s). You should upload this letter as a separate file labeled 'Response to Reviewers'.
- A marked-up copy of your manuscript that highlights changes made to the original version. You should upload this as a separate file labeled 'Revised Manuscript with Track Changes'.
- An unmarked version of your revised paper without tracked changes. You should upload this as a separate file labeled 'Manuscript'.

If you would like to make changes to your financial disclosure, please include your updated statement in your cover letter. Guidelines for resubmitting your figure files are available below the reviewer comments at the end of this letter.

If applicable, we recommend that you deposit your laboratory protocols in protocols.io to enhance the reproducibility of your results. Protocols.io assigns your protocol its own identifier (DOI) so that it can be cited independently in the future. For instructions see: https://journals.plos.org/plosone/s/submission-guidelines#loc-laboratory-protocols. Additionally, PLOS ONE offers an option for publishing peer-reviewed Lab Protocol articles, which describe protocols hosted on protocols.io. Read more information on sharing protocols at https://plos.org/protocols?utm_medium=editorial-email&utm_source=authorletters&utm_campaign=protocols.

We look forward to receiving your revised manuscript.

Kind regards,

Paavani Atluri

Academic Editor

PLOS ONE

Journal Requirements:

When submitting your revision, we need you to address these additional requirements.

1. Please ensure that your manuscript meets PLOS ONE's style requirements, including those for file naming. The PLOS ONE style templates can be found at

https://journals.plos.org/plosone/s/file?id=wjVg/PLOSOne_formatting_sample_main_body.pdf and https://journals.plos.org/plosone/s/file?id=ba62/PLOSOne_formatting_sample_title_authors_affiliations.pdf

2. We note that the grant information you provided in the ‘Funding Information’ and ‘Financial Disclosure’ sections do not match.

When you resubmit, please ensure that you provide the correct grant numbers for the awards you received for your study in the ‘Funding Information’ section.

3. Thank you for stating the following financial disclosure:

[Scientific Research Grant from Shenzhen Society of Health Economics].

Please state what role the funders took in the study.  If the funders had no role, please state: "The funders had no role in study design, data collection and analysis, decision to publish, or preparation of the manuscript."

If this statement is not correct you must amend it as needed.

Please include this amended Role of Funder statement in your cover letter; we will change the online submission form on your behalf.

4. Thank you for stating the following in your Competing Interests section:

[no].

Please complete your Competing Interests on the online submission form to state any Competing Interests. If you have no competing interests, please state "The authors have declared that no competing interests exist.", as detailed online in our guide for authors at http://journals.plos.org/plosone/s/submit-now

This information should be included in your cover letter; we will change the online submission form on your behalf.

5. When completing the data availability statement of the submission form, you indicated that you will make your data available on acceptance. We strongly recommend all authors decide on a data sharing plan before acceptance, as the process can be lengthy and hold up publication timelines. Please note that, though access restrictions are acceptable now, your entire data will need to be made freely accessible if your manuscript is accepted for publication. This policy applies to all data except where public deposition would breach compliance with the protocol approved by your research ethics board. If you are unable to adhere to our open data policy, please kindly revise your statement to explain your reasoning and we will seek the editor's input on an exemption. Please be assured that, once you have provided your new statement, the assessment of your exemption will not hold up the peer review process.

6. Your ethics statement should only appear in the Methods section of your manuscript. If your ethics statement is written in any section besides the Methods, please delete it from any other section.

If the reviewer comments include a recommendation to cite specific previously published works, please review and evaluate these publications to determine whether they are relevant and should be cited. There is no requirement to cite these works unless the editor has indicated otherwise.

[Note: HTML markup is below. Please do not edit.]

Reviewers' comments:

Reviewer's Responses to Questions

**Comments to the Author**

1. Is the manuscript technically sound, and do the data support the conclusions?

Reviewer #1: Partly

Reviewer #2: No

**********

2. Has the statistical analysis been performed appropriately and rigorously?

Reviewer #1: Yes

Reviewer #2: Yes

**********

3. Have the authors made all data underlying the findings in their manuscript fully available?

The PLOS Data policy

Reviewer #1: No

Reviewer #2: Yes

**********

4. Is the manuscript presented in an intelligible fashion and written in standard English?

Reviewer #1: Yes

Reviewer #2: Yes

**********

Reviewer #1: Dear Author:

Thank you for submitting the manuscript. PLOS ONE. After a thorough review, I regret to inform you that the manuscript in its current form does not meet the standards required for publication. My concerns are as follows:

First, in determining the specific diseaseWhen considering clinical risk factors, the timing of data collection (especially exploratory indicators) is crucial. In this study, the authors failed to clarify...PaO2and CRT The temporal relationship between developments. However, as far as I know,MIMIC dataThe library does indeed allow researchers to determine the temporal sequence between disease events and laboratory indicators. Logically, your conclusion seems to be based on data collected before thrombosis.PaO2——noTherefore, I strongly urge the authors to clearly and specifically detail the inclusion criteria and the timing of data collection in the manuscript.

SecondlyAlthough the manuscript title indicatesPaO2yes CRT A dangerWhile there are risk factors, intraductal thrombosis can be influenced by a variety of factors, not just one. PaO₂I acceptThe author's extensive and exploratory analysis is commendable.;However,There is a clear disconnect between the title and the actual content of the manuscript. I suggest revising the title to more accurately reflect the scope and findings of the research.。

Finally, return in segments.Regression analysis to determine PaO₂ Threshold is 384 mmHg，This is far higher than commonly observed clinical values. Such levels can only be achieved with pure oxygen inhalation or hyperbaric oxygen therapy. Therefore, the clinical relevance and applicability of this threshold are highly questionable.

I sincerely hope the author canThese key issues were taken seriously, and the manuscript was revised accordingly.

Yours sincerely

Reviewpeople

Reviewer #2: Evaluating risk factors for catheter-related thrombosis in critically ill patients is an important issue.

However, the article requires significant revisions.

There are several publications highlighting the harmful effects of hyperoxia in critically ill patients — this topic should be discussed in the manuscript.

Introduction – I recommend revising the first paragraph to make the message clearer.

We know that the prevalence of thrombosis varies depending on the type of catheter used — I suggest that the authors describe the types of catheters (if available) and provide a discussion on this point.

Did the hospitals have protocols for diagnosing thrombosis? How were the thrombosis cases assessed? Complementary examinations are not described.

I suggest revising Table 1 – for instance, regarding vasopressin, it would be more appropriate to present only the proportion of patients who used it, as well as for anticoagulants.

Table 2 is difficult to interpret due to its layout. I recommend the authors reformat it for clarity.

Finally, I suggest the authors revisit the limitations section of the study, which should include the heterogeneity of the participating centers, the presence or absence of diagnostic protocols, and a discussion on the limitations inherent to a retrospective study.

**********

what does this mean?). If published, this will include your full peer review and any attached files.

If you choose “no”, your identity will remain anonymous but your review may still be made public.

**Do you want your identity to be public for this peer review?**  For information about this choice, including consent withdrawal, please see our Privacy Policy

Reviewer #1: No

Reviewer #2: No

**********

[NOTE: If reviewer comments were submitted as an attachment file, they will be attached to this email and accessible via the submission site. Please log into your account, locate the manuscript record, and check for the action link "View Attachments". If this link does not appear, there are no attachment files.]

To ensure your figures meet our technical requirements, please review our figure guidelines: https://journals.plos.org/plosone/s/figures

You may also use PLOS’s free figure tool, NAAS, to help you prepare publication quality figures: https://journals.plos.org/plosone/s/figures#loc-tools-for-figure-preparation.

NAAS will assess whether your figures meet our technical requirements by comparing each figure against our figure specifications.

# Author comment: Author response to Decision Letter 1

**DOI:** 10.1371/journal.pone.0359719.r002

9 Mar 2026

Response to Reviewers

Manuscript ID: PONE-D-25-27701

Title: Higher Partial Pressure of Oxygen is Associated with Reduced Risk of Catheter-Related Thrombosis in Critical Care: Analysis of Threshold Effects Using the MIMIC Database

Dear Academic Editor and Reviewers,

Thank you very much for your thoughtful and constructive feedback on our manuscript. We greatly appreciate the time and expertise you have devoted to reviewing our work. We have carefully considered all comments and have revised the manuscript accordingly. Below, we provide a point-by-point response to each concern raised.

________________________________________

Response to Reviewer #1

Comment 1: The authors failed to clarify the temporal relationship between PaO₂ and CRT development… I strongly urge the authors to clearly and specifically detail the inclusion criteria and the timing of data collection.

We sincerely thank the reviewer for this crucial observation and apologize for the lack of clarity in our original manuscript.

We fully understand the reviewer’s concern. Establishing a valid temporal sequence—where exposure (PaO₂) precedes outcome (CRT)—is fundamental for causal inference in observational studies. Without this clarification, the validity of our conclusions could be questioned.

We confirm that all PaO₂ values used in our analysis were extracted from the first arterial blood gas measurement recorded after ICU admission and, critically, before the diagnosis of CRT (or before ICU discharge for patients who did not develop CRT). This ensures that the exposure temporally precedes the outcome.

We have revised the manuscript as follows:

Original: “The primary exposure variable was arterial oxygen partial pressure (PaO2), which was categorized into three groups… These measurements were obtained from arterial blood gas analyses during the ICU stay.”

Revised: “The primary exposure variable was the first recorded arterial oxygen partial pressure (PaO₂) during the ICU stay, which was confirmed to have occurred prior to the diagnosis of catheter-related thrombosis. This PaO₂ value was categorized into three groups: ≤80 mmHg, >80 to ≤100 mmHg, and >100 mmHg.”

Additionally, we added a sentence in the Statistical Analysis section: “All exposure data (PaO₂) were ascertained before the outcome event (CRT) to establish a valid temporal sequence.”

________________________________________

Comment 2: There is a clear disconnect between the title and the actual content… I suggest revising the title.

We thank the reviewer for this helpful suggestion.

We agree that the original title could be misleading, as it labeled PaO₂ a “risk factor,” whereas our findings indicate it is a protective factor (higher PaO₂ associated with lower CRT risk).

We have revised the title to more accurately reflect our findings:

Original: “Partial Pressure of Oxygen as a Risk Factor for Catheter-Related Thrombosis in Critical Care…”

Revised: “Higher Partial Pressure of Oxygen is Associated with Reduced Risk of Catheter-Related Thrombosis in Critical Care: Analysis of Threshold Effects Using the MIMIC Database”.

________________________________________

Comment 3: The threshold of 384 mmHg is far higher than clinical values… clinical relevance is highly questionable.

We sincerely appreciate the reviewer’s insightful clinical perspective and acknowledge this important concern.

We understand that a PaO₂ of 384 mmHg is supraphysiological and rarely a clinical target, as it typically requires high-concentration oxygen therapy, which carries risks of oxygen toxicity.

In our revision, we clarify that this threshold should be interpreted as a statistical inflection point—not a therapeutic goal. The key clinical message is that maintaining PaO₂ above hypoxemic levels (e.g., >80 mmHg) is protective, and no additional CRT benefit is seen beyond extremely high levels. We now explicitly caution against pursuing supraphysiological PaO₂ for CRT prevention alone.

Revised text in the Discussion:

Original: “Our findings provide valuable clinical reference for oxygen therapy management in ICU patients. The identification of a 384 mmHg PO2 threshold enables clinicians to optimize individualized oxygen therapy protocols…”

Revised: “However, our identification of a PO₂ threshold at 384 mmHg should be interpreted with caution. This value far exceeds typical clinical oxygenation targets and is often only achievable with high or pure oxygen supplementation, which carries its own risks of oxygen toxicity and lung injury(11). Therefore, this threshold should be viewed primarily as a statistical inflection point indicating the saturation of the protective effect against CRT, rather than a therapeutic goal. The more clinically relevant and actionable finding is that maintaining PO₂ above the hypoxemic range (e.g., >80 mmHg) is consistently associated with a significantly reduced risk of CRT. Clinicians should aim for adequate oxygenation to avoid hypoxemia, but should not pursue supraphysiological PO₂ levels solely for the purpose of CRT prevention, given the potential for harm from hyperoxia. Several publications have highlighted the harmful effects of hyperoxia in critically ill patients, including increased oxidative stress, ventilator-induced lung injury, and higher mortality in subgroups such as those with acute respiratory distress syndrome (ARDS) or post-cardiac arrest(12-14). For instance, Girardis et al. (12) in a randomized trial showed that conservative oxygen therapy (targeting PaO₂ 70-100 mmHg) reduced mortality compared to liberal strategies (PaO₂ up to 150 mmHg). This underscores the need to avoid hyperoxia while preventing hypoxemia. The observed association between hypoxemia and increased thrombosis risk is biologically plausible and aligns with previous mechanistic studies. Under hypoxic conditions, the activation of Hypoxia-Inducible Factors (HIFs) promotes thrombosis, though current risk models do not include oxygenation status as a specific factor (20)(21). Our study suggests that a patient's oxygenation level could be a relevant variable to consider in future risk stratification models. Further prospective studies are warranted to determine whether optimizing oxygenation, while strictly avoiding both hypoxia and hyperoxia, can be an effective component of a multifaceted strategy to prevent CRT in ICU patients.”

Revised text in the conclusion

Original: Higher PO2 levels are associated with reduced CRT risk in ICU patients, with an optimal threshold at 384 mmHg. These findings suggest the importance of maintaining adequate oxygenation for CRT prevention while avoiding excessive oxygen supplementation.

Revised: Our findings suggest that avoiding hypoxemia—not inducing hyperoxia—is the clinically relevant strategy for CRT prevention

________________________________________

Response to Reviewer #2

Comment 1: Discuss the harmful effects of hyperoxia in critically ill patients.

We sincerely appreciate the reviewer’s suggestion to expand the discussion on the harmful effects of hyperoxia in critically ill patients, as this ensures a more balanced and clinically relevant presentation.

In response, we have expanded the Discussion section to include a dedicated paragraph on hyperoxia's adverse effects, incorporating additional references to key publications for a comprehensive view.

Revised text in the introduction

Original: However, excessive oxygen levels may also have detrimental effects, highlighting the complexity of this relationship.

Revised: However, excessive oxygen levels may also have detrimental effects, highlighting the complexity of this relationship(11). Nevertheless, hyperoxia can lead to adverse outcomes such as oxidative stress and lung injury, necessitating a balanced approach(12-14).

Revised text in the discussion

This is now integrated into our interpretation of the 384 mmHg threshold (see response to Reviewer #1, Comment 3).________________________________________

Comment 2: Revise the first paragraph of the Introduction for clarity.

We sincerely appreciate the reviewer's suggestion to revise the first paragraph of the Introduction for greater clarity, as this helps ensure the manuscript is more accessible to readers.

In response, we have revised the first paragraph to make the message clearer by reorganizing the content with better flow, shorter sentences, and explicit transitions.

Original: Catheter-related thrombosis (CRT) represents a significant complication in intensive care unit (ICU) patients(1). Recent multicenter studies have reported that central venous catheters are utilized in over 85% of ICU patients, with CRT incidence rates varying considerably from 3.6% to 59%(2). This complication not only prolongs hospital stays and increases healthcare costs but also poses substantial risks for life-threatening complications such as pulmonary embolism(3). Furthermore, CRT has been associated with increased mortality rates and adverse clinical outcomes in critically ill patients(4)

Revised: Catheter-related thrombosis (CRT) is a major complication in intensive care unit (ICU) patients, affecting over 85% of those with central venous catheters(1,2). Incidence rates range from 3.6% to 59%, leading to prolonged hospital stays, increased healthcare costs, and severe risks such as pulmonary embolism and higher mortality(3,4). These impacts highlight the urgent need for better prevention strategies

________________________________________

Comment 3: Describe catheter types if available.

We acknowledge this limitation. The MIMIC-IV database identifies CRT via diagnosis codes but does not specify catheter types (e.g., PICC vs. CVC).

We have added this as a limitation: “Fourth, the MIMIC-IV database does not provide granular details on the catheters used, including material type (e.g., polyurethane vs. silicone). Given that these factors are well-established determinants of thrombosis risk, their absence constitutes a significant limitation and may introduce unmeasured heterogeneity into our findings.”

________________________________________

Comment 4: Clarify how thrombosis was diagnosed.

We appreciate the reviewer’s attention to the details of the diagnostic methods and thank you for emphasizing their importance to the reliability of our study.

We agree with your suggestion and have added further details in the Methods section under “Outcome Measurement,” describing the diagnostic assessment approach based on the MIMIC database. We have expanded the Outcome Measurement section:

Original: The primary outcome was the development of catheter-related thrombosis during the ICU stay. Thrombosis was identified through documented diagnosis codes and clinical notes.

Revised: The primary outcome was the development of catheter-related thrombosis during the ICU stay. Thrombosis was identified through documented ICD-9/10 diagnosis codes (e.g., 453.81 for acute venous embolism and thrombosis) and clinical notes in the MIMIC-IV database. Diagnosis typically involved complementary examinations such as duplex ultrasound or computed tomography angiography, as per standard protocols at Beth Israel Deaconess Medical Center, though specific per-case details are not uniformly recorded.

________________________________________

Comment 5: Improve Tables 1 and 2.

we sincerely thank the reviewer for their valuable suggestions regarding the readability and presentation of our tables. we fully understand the reviewer’s concerns: in the original Table 1, the reporting of both "yes" and "no" percentages for binary variables such as vasopressin and anticoagulant use was redundant; and the original layout of Table 2 was overly compact, making it difficult to interpret the association estimates across different adjustment models. In response, we have carefully revised both tables in accordance with the reviewer’s recommendations to enhance clarity and data accessibility.

The specific revisions are as follows:

Table 1: For vasopressin and anticoagulant use, we now report only the proportion of patients who received these interventions (i.e., the “Yes” percentage), removing the redundant “No” category.

Table 2: We have completely reformatted the table to clearly separate the results for continuous and categorical PO₂ analyses, and to align the odds ratios (ORs), 95% confidence intervals (CIs), and P-values for the non-adjusted, Model I, and Model II regression models in a vertically structured and reader-friendly format.

________________________________________

Comment 6: Expand the limitations section.

we sincerely thank the reviewer for their insightful comments regarding the limitations of our study. we fully understand the reviewer’s concerns. Although the MIMIC-IV database is derived from a single-center ICU (Beth Israel Deaconess Medical Center), the retrospective nature of our analysis, the absence of standardized diagnostic protocols for catheter-related thrombosis, and the lack of detailed information on catheter characteristics (such as material type) represent important methodological constraints that warrant explicit acknowledgment.

In response, we sincerely thank the reviewer for their insightful comments regarding the limitations of our study.

Original: “…unmeasured confounders such as genetic background and specific medication regimens.

Revised: This study has several limitations that warrant consideration. First, our analysis is based on the MIMIC-IV database, which is a single-center retrospective cohort. While this provides a large and detailed dataset, the findings may not be fully generalizable to other healthcare settings or populations with different baseline characteristics. Second, as a retrospective study, our analysis is inherently susceptible to residual confounding and selection bias. Although we adjusted for a comprehensive set of clinically relevant covariates, unmeasured or unrecorded confounders (e.g., specific genetic predispositions, detailed medication regimens, or fluid balance) may still influence the observed associations. Third, the diagnosis of catheter-related thrombosis (CRT) relied on ICD coding and supporting clinical documentation, without a standardized, protocol-driven diagnostic approach across the ICU. This may introduce outcome misclassification, potentially leading to either under- or over-estimation of the true CRT incidence. Fourth, the MIMIC-IV database does not provide granular details on the catheters used, including material type (e.g., polyurethane vs. silicone). Given that these factors are well-established determinants of thrombosis risk, their absence constitutes a significant limitation and may introduce unmeasured heterogeneity into our findings. Finally, our data spans from 2008 to 2019, and clinical practices regarding both oxygen therapy and thromboprophylaxis may have evolved since then, which could affect the contemporary applicability of our results.

________________________________________

We believe these revisions have significantly strengthened the manuscript. Thank you again for your valuable feedback.

Sincerely,

Qing Cui, Anshuai Fang, Haiyan Zhang, Qingtong Meng, Sijia Zhou, Yueming Peng, Weixiang Luo

Attachment Submitted filename: Response to Reviewers.docx

# Peer review: Decision Letter 1

**DOI:** 10.1371/journal.pone.0359719.r003

**Authors:** Giovanni Giordano

15 Jun 2026

Dear Dr. Cui,

Thank you for submitting your manuscript to PLOS ONE. After careful consideration, we feel that it has merit but does not fully meet PLOS ONE’s publication criteria as it currently stands. Therefore, we invite you to submit a revised version of the manuscript that addresses the points raised during the review process.

Please submit your revised manuscript by Jul 30 2026 11:59PM. If you will need more time than this to complete your revisions, please reply to this message or contact the journal office at plosone@plos.org. When you're ready to submit your revision, log on to https://www.editorialmanager.com/pone/ and select the 'Submissions Needing Revision' folder to locate your manuscript file.

- A letter that responds to each point raised by the academic editor and reviewer(s). You should upload this letter as a separate file labeled 'Response to Reviewers'.
- A marked-up copy of your manuscript that highlights changes made to the original version. You should upload this as a separate file labeled 'Revised Manuscript with Track Changes'.
- An unmarked version of your revised paper without tracked changes. You should upload this as a separate file labeled 'Manuscript'.

If you would like to make changes to your financial disclosure, please include your updated statement in your cover letter. Guidelines for resubmitting your figure files are available below the reviewer comments at the end of this letter.

If applicable, we recommend that you deposit your laboratory protocols in protocols.io to enhance the reproducibility of your results. Protocols.io assigns your protocol its own identifier (DOI) so that it can be cited independently in the future. For instructions see: https://journals.plos.org/plosone/s/submission-guidelines#loc-laboratory-protocols. Additionally, PLOS ONE offers an option for publishing peer-reviewed Lab Protocol articles, which describe protocols hosted on protocols.io. Read more information on sharing protocols at https://plos.org/protocols?utm_medium=editorial-email&utm_source=authorletters&utm_campaign=protocols.

As the corresponding author, your ORCID iD is verified in the submission system and will appear in the published article. PLOS supports the use of ORCID, and we encourage all coauthors to register for an ORCID iD and use it as well. Please encourage your coauthors to verify their ORCID iD within the submission system before final acceptance, as unverified ORCID iDs will not appear in the published article. *Only*  the individual author can complete the verification step; PLOS staff *cannot*  verify ORCID iDs on behalf of authors.

We look forward to receiving your revised manuscript.

Kind regards,

Giovanni Giordano

Academic Editor

PLOS One

**Journal Requirements:**

If the reviewer comments include a recommendation to cite specific previously published works, please review and evaluate these publications to determine whether they are relevant and should be cited. There is no requirement to cite these works unless the editor has indicated otherwise.

[Note: HTML markup is below. Please do not edit.]

Reviewers' comments:

Reviewer's Responses to Questions

**Comments to the Author**

Reviewer #1: All comments have been addressed

Reviewer #3: (No Response)

Reviewer #4: (No Response)

**********

2. Is the manuscript technically sound, and do the data support the conclusions?

Reviewer #1: No

Reviewer #3: Partly

Reviewer #4: Partly

**********

3. Has the statistical analysis been performed appropriately and rigorously?

Reviewer #1: No

Reviewer #3: No

Reviewer #4: No

**********

4. Have the authors made all data underlying the findings in their manuscript fully available?

The PLOS Data policy

Reviewer #1: Yes

Reviewer #3: Yes

Reviewer #4: Yes

**********

5. Is the manuscript presented in an intelligible fashion and written in standard English?

Reviewer #1: Yes

Reviewer #3: (No Response)

Reviewer #4: Yes

**********

Reviewer #1: The authors have substantially revised the manuscript, and their effort is appreciated. However, the revised manuscript still does not resolve the fundamental methodological concerns raised in the previous review.

First, the temporal relationship between PaO₂ measurement and CRT development remains insufficiently clarified. If an arterial blood gas value obtained after ICU admission is used as the exposure for catheter-related thrombosis, this value must have a clear and clinically meaningful temporal relationship with catheter insertion, catheter dwell time, and thrombosis development. For example, if a patient with shock undergoes arterial blood gas testing immediately after ICU admission and has a CVC inserted at that time, but develops deep venous thrombosis on ICU day 10, it remains questionable whether the initial PaO₂ value can reasonably represent the relevant exposure for that catheter-related thrombotic event. In the revised manuscript, the authors only state that the first recorded PaO₂ during ICU stay occurred before CRT diagnosis. This statement does not adequately address the concern. Demonstrating that PaO₂ preceded the diagnostic code for CRT is not sufficient to establish a valid exposure window. The authors still do not report the interval from ICU admission to PaO₂ measurement, from catheter insertion to PaO₂ measurement, or from PaO₂ measurement to CRT diagnosis. Nor do they show whether the timing of blood gas sampling was comparable between patients with and without CRT.

Second, the reported threshold of 384 mmHg remains clinically uninterpretable. Although the title has been revised, the authors continue to retain this threshold as an important finding. They acknowledge that this value far exceeds usual clinical oxygenation targets and should be interpreted as a statistical inflection point rather than a therapeutic goal. However, this does not resolve the problem. In real ICU practice, a PaO₂ of 384 mmHg is uncommon and usually reflects transient exposure to very high FiO₂, peri-intubation or perioperative oxygen administration, resuscitation, or other specific oxygenation contexts rather than a stable and clinically generalizable oxygenation target. Therefore, this threshold has very limited clinical relevance.

More importantly, the 384 mmHg cutoff may be an artifact arising from the construction of the MIMIC-based dataset and the modeling strategy. Potential sources of bias include indication bias in arterial blood gas sampling, exposure misclassification due to the absence of FiO₂, PEEP, oxygen delivery method, mechanical ventilation status, and PaO₂/FiO₂ ratio, sparse data in the high-PaO₂ tail, extreme-value effects, case-mix heterogeneity, unmeasured catheter dwell time and catheter type, and outcome misclassification due to reliance on ICD codes and clinical notes. The authors do not report the distribution of PaO₂ values, the number of patients with PaO₂ >384 mmHg, the number of CRT events above this threshold, the handling of outliers, or sensitivity analyses excluding extreme PaO₂ values. Therefore, the threshold should not be presented as a clinically meaningful finding.

Overall, the authors have not truly addressed the major concern raised in the previous review. At minimum, they should provide detailed timing information linking PaO₂ measurement to ICU admission, catheter insertion, catheter dwell time, and CRT diagnosis. If the MIMIC database cannot provide the variables required to establish a valid exposure window and clinically interpretable oxygenation context, then this database may not be suitable for answering the present research question. The authors should consider validating or re-conducting the study using original clinical data from their own center, where catheter timing, catheter characteristics, oxygenation parameters, and CRT diagnosis can be reliably verified.

For these reasons, I do not recommend acceptance of the manuscript in its current form.

Reviewer #3: Manuscript ID: PONE-D-25-27701R1

Dear Editor,

Thank you very much for the invitation to review this manuscript titled “Higher Partial Pressure of Oxygen is Associated with Reduced Risk of Catheter-Related Thrombosis in Critical Care: Analysis of Threshold Effects Using the MIMIC Database” and for the opportunity to contribute to the evaluation process for PLOS ONE. I would also like to thank the authors for their work on a clinically relevant question and for the careful, point-by-point responses provided in this revised version, which have clearly improved the clarity of the manuscript.

In brief, the authors use the MIMIC-IV database (2008–2019) to investigate the association between arterial partial pressure of oxygen (PaO₂) and catheter-related thrombosis (CRT) in 13,328 adult ICU patients with upper-extremity catheters. PaO₂ was analysed both as a continuous variable and in three categories (≤80, >80–100, >100 mmHg), with progressively adjusted logistic regression models, smooth-curve fitting, segmented (threshold) regression, and subgroup analyses by anticoagulation status, SOFA score and age. Compared with PaO₂ ≤80 mmHg, higher PaO₂ categories were associated with a lower adjusted risk of CRT (adjusted OR 0.49 and 0.47), and the authors report a threshold at 384 mmHg beyond which no further benefit was seen. The revised manuscript reframes this finding as avoidance of hypoxemia rather than induction of hyperoxia, which is appropriate.

My recommendation is MAJOR REVISION. The dataset and question are of interest and the writing has improved, but several methodological and interpretive issues need to be resolved before the conclusions can be considered robust. My specific concerns, in order of importance, are as follows:

1. In Table 3, the effect above the 384 mmHg breakpoint is reported as OR = 0.00 (95% CI 0.00, Inf), p = 0.9795. An odds ratio of zero with an infinite upper bound indicates complete separation / absence of events (or vanishingly few patients) above the breakpoint, i.e. a non-interpretable estimate rather than evidence of a true plateau. Because this threshold now features prominently in both the title and the abstract, it must be substantiated: please report the number of patients and the number of CRT events above 384 mmHg, and reconsider whether the segmented model is adequately powered in that range. If the estimate is driven by sparse data, the threshold should be presented with strong caveats or removed from the title/abstract.

2. When PaO₂ is modelled continuously, the adjusted OR is 1.00 (95% CI 0.99–1.00) per 1 mmHg, with significance driven almost entirely by the large sample size. Describing this as a “significant inverse association” risks overstating a near-null per-unit effect. Please report the OR per a clinically meaningful increment (e.g. per 10 mmHg) and temper the wording accordingly.

3. Patients with PaO₂ >100 mmHg constitute the large majority of the cohort (9,242/13,328) and very likely reflect patients receiving supplemental oxygen or mechanical ventilation, whereas low PaO₂ marks respiratory failure and greater illness. Although APSIII and SOFA were adjusted for, key drivers such as FiO₂, mechanical ventilation, and the PaO₂/FiO₂ ratio are not included. A PaO₂ of 384 mmHg is essentially attainable on high FiO₂, so the exposure is partly a proxy for the treatment received. Please adjust for FiO₂ / ventilation where available, or discuss this as a major source of residual confounding and reverse causation.

4. CRT was identified from ICD-9/10 codes and clinical notes. By the authors’ own cited literature, a large fraction of CRT is asymptomatic (incidence reported up to 59%), yet the observed incidence here is only ~2.2% in the reference group. This strongly suggests that only symptomatic / imaged thrombosis was captured, and detection may be differential (sicker or more frequently imaged patients are more likely to be diagnosed). Please report the total number of CRT events, and discuss the direction of this detection bias; it could plausibly generate the observed association if low-PaO₂ patients are imaged more often.

5. I appreciate the clarification that the first PaO₂ before CRT was used. However, a single baseline value cannot capture the dynamic oxygenation of an ICU stay, and the differing exposure windows introduce an immortal-time-like imbalance. A time-to-event model with PaO₂ as a time-varying exposure would be more appropriate; at minimum, this limitation should be stated explicitly.

6. Reporting completeness (STROBE). Please add the number of patients excluded for missing key variables and a comparison of their characteristics; the number of CRT events per PaO₂ group; and some assessment of model performance (calibration/discrimination). This information is needed to judge the stability of the adjusted estimates given the low event rate.

7. The biological rationale leans heavily on COVID-19 cohorts (e.g. Helms et al., Tang et al.). Coagulopathy in severe SARS-CoV-2 infection may not generalise to unselected ICU patients with catheter-related thrombosis; please acknowledge this and, where possible, cite mechanistic evidence from a broader critical-care population.

A few minor points would also strengthen the manuscript:

8. Table 1 still contains misaligned and internally inconsistent cells; please verify all values, units, and decimal places.

9. The notation alternates between “PO2” and “PaO₂” throughout the text, tables and figure legends; please standardise to PaO₂.

10. Figure 2 should state the number of observations and ideally show the distribution of PaO₂ values, given the wide confidence bands at the extremes.

In summary, this is a potentially useful real-world analysis, but the threshold claim that anchors the title and abstract currently rests on a non-estimable result, and the association is vulnerable to confounding by ventilation and to outcome misclassification.

Addressing points these points is essential before the conclusions can be supported. I believe these issues are addressable, and I would be glad to review a revised version.

I thank the Editor again for the invitation and the authors for their efforts on this work.

Sincerely,

The Reviewer

Reviewer #4: This manuscript investigates the association between arterial oxygen partial pressure (PaO₂) and catheter-related thrombosis (CRT) among ICU patients using the MIMIC-IV database. The topic is clinically relevant because CRT is a common ICU complication and oxygenation status is routinely measured. The large sample size is a major strength, and the authors attempt to explore nonlinear relationships and threshold effects.

However, substantial methodological and interpretative concerns currently limit confidence in the conclusions. The central finding—that higher PaO₂ protects against CRT—may be heavily influenced by confounding, selection bias, exposure misclassification, outcome ascertainment issues, and possible immortal-time bias. Several analytical choices require clarification or re-analysis before publication.

The manuscript has merit but requires major revision.

A. Major Comments

1. Fundamental Concern Regarding CRT Ascertainment

The manuscript states:

"CRT was identified through ICD-9/10 diagnosis codes and clinical notes."

This approach raises major concerns.

CRT is notoriously undercoded in administrative databases. Many upper-extremity catheter thromboses are diagnosed radiologically but never assigned specific ICD codes. Conversely, some ICD thrombotic diagnoses may represent unrelated venous thromboses.

The authors should provide:

Exact ICD-9 and ICD-10 codes used.

Validation strategy.

Sensitivity and specificity of their CRT definition.

Whether thromboses were restricted to upper-extremity catheter-associated events.

How non-catheter-related DVTs were excluded.

Without a validated CRT definition, outcome misclassification could substantially bias results.

2. Exposure Definition is Potentially Problematic

The study uses:

"First recorded arterial PaO₂ during ICU stay."

This single measurement is unlikely to represent oxygen exposure throughout the period during which CRT develops.

Important questions:

How soon after ICU admission was PaO₂ measured?

What was the median time from PaO₂ measurement to CRT diagnosis?

Were patients already thrombosed when the first blood gas was obtained?

Why was only the first PaO₂ used instead of:

Time-weighted average PaO₂

Maximum PaO₂

Minimum PaO₂

Area-under-the-curve exposure

Given that oxygenation changes dramatically during ICU admission, reliance on a single value may create substantial exposure misclassification.

3. Temporal Relationship Remains Unclear

The manuscript repeatedly claims:

"PaO₂ occurred before CRT diagnosis."

However, the methodology does not demonstrate this rigorously.

The authors should explicitly report:

Time of catheter insertion.

Time of first PaO₂ measurement.

Time of CRT diagnosis.

CRT is a time-dependent event. Standard logistic regression ignores timing and may introduce bias.

A more appropriate approach would be:

Cox proportional hazards model.

Time-to-event analysis.

Competing-risk framework (death as competing event).

Current analyses do not adequately address temporality.

4. Severe Risk of Confounding by Illness Severity

One striking observation:

Patients with PaO₂ >100 mmHg appear less ill:

Variable ≤80 mmHg >100 mmHg

APSIII 4.04 3.76

SOFA 7.64 6.37

Vasopressor use 18.0% 10.1%

ICU LOS 1.70 1.20

These differences are substantial.

Higher PaO₂ may simply identify patients who:

Are physiologically more stable.

Have better pulmonary function.

Have shorter ICU stays.

Have lower inflammatory burden.

Thus PaO₂ may function as a marker of illness severity rather than an independent causal factor.

Although adjustment was performed, residual confounding remains highly likely.

I strongly recommend:

Propensity score matching.

IPTW analysis.

Sensitivity analyses using doubly robust models.

5. Potential Reverse Causality

Patients with severe illness often develop:

Hypoxemia

Longer catheter dwell times

More thrombosis

Thus:

Hypoxemia → severity ← CRT

rather than:

Hypoxemia → CRT

The authors should discuss this possibility more thoroughly.

Current wording occasionally implies causality that cannot be supported by observational data.

6. Threshold Analysis Appears Statistically Fragile

The reported threshold:

384 mmHg

is difficult to interpret biologically.

Several concerns arise:

A. Sparse Data

Very few ICU patients likely have PaO₂ >384 mmHg.

The manuscript does not provide:

Number of patients above threshold.

Distribution of PaO₂ values.

Histogram or density plot.

B. Implausible Estimate

Table 3 reports:

OR = 0.00 (0.00–Inf)

This indicates model instability.

Such estimates typically occur when:

Data are sparse.

Separation occurs.

Regression becomes unreliable.

Therefore the claimed threshold may be an artifact of modeling.

The threshold analysis should be interpreted cautiously or potentially removed.

7. Inconsistency Between Continuous and Categorical Analyses

The manuscript reports:

Continuous model:

OR ≈ 1.00

Categorical model:

OR ≈ 0.47–0.49

These findings appear discordant.

If a clinically meaningful effect exists, the continuous estimate should better reflect that relationship.

This discrepancy suggests:

Strong nonlinearity,

Modeling issues,

Or influence of categorization.

The authors should formally compare models and present spline plots with confidence intervals.

8. Anticoagulation Variable May Introduce Bias

The study adjusts for anticoagulant administration.

However:

Anticoagulation may lie on the causal pathway.

Patients perceived as thrombosis-prone are more likely to receive anticoagulation.

Adjusting for this variable may create:

Collider bias

Overadjustment

The authors should justify its inclusion and perform sensitivity analyses excluding anticoagulation.

9. Missing Important CRT Risk Factors

Several important determinants of CRT are absent:

Catheter-related

Catheter type

Number of lumens

Catheter size

PICC vs CVC

Insertion technique

Catheter dwell time

Tip position

Clinical

Mechanical ventilation

Cancer status

Sepsis

Platelet count

D-dimer

Coagulation profile

These omissions substantially limit causal inference.

10. Use of the Term "Protective Effect"

The manuscript repeatedly uses phrases such as:

"protective effect"

"risk reduction"

These imply causality.

Given the observational design, wording should be changed to:

"associated with lower odds"

"inverse association"

"correlated with reduced CRT occurrence"

throughout the manuscript.

B. Minor Comments

1. Study Design Misclassification

The manuscript alternates between:

Retrospective cohort

Cross-sectional study

These are not equivalent.

The study should be consistently described as a retrospective observational cohort analysis.

2. PO₂ versus PaO₂

Terminology switches repeatedly between:

PO₂

PaO₂

Use PaO₂ consistently because arterial oxygen tension is specifically being measured.

3. APACHE III / APSIII Confusion

The manuscript uses:

APSIII

APACHE III

interchangeably.

Please clarify which score was included in regression models.

4. Reporting of Continuous ORs

Reporting:

OR=1.00

is uninformative.

Authors should present:

OR per 10 mmHg increase

or

OR per SD increase

to improve interpretability.

5. Missing Handling of Missing Data

The manuscript states:

Missing key variables were excluded.

The following should be reported:

Number excluded.

Variables missing.

Missingness percentages.

Whether multiple imputation was considered.

6. MIMIC-IV Reporting Standards

Authors should provide:

Exact SQL extraction strategy.

MIMIC-IV version used.

Reproducible code repository.

This aligns with current expectations for MIMIC-based publications.

**********

what does this mean?). If published, this will include your full peer review and any attached files.

If you choose “no”, your identity will remain anonymous but your review may still be made public.

**Do you want your identity to be public for this peer review?**  For information about this choice, including consent withdrawal, please see our Privacy Policy

Reviewer #1: No

Reviewer #3: No

Reviewer #4: No

**********

[NOTE: If reviewer comments were submitted as an attachment file, they will be attached to this email and accessible via the submission site. Please log into your account, locate the manuscript record, and check for the action link "View Attachments". If this link does not appear, there are no attachment files.]

To ensure your figures meet our technical requirements, please review our figure guidelines: https://journals.plos.org/plosone/s/figures

You may also use PLOS’s free figure tool, NAAS, to help you prepare publication quality figures: https://journals.plos.org/plosone/s/figures#loc-tools-for-figure-preparation.

NAAS will assess whether your figures meet our technical requirements by comparing each figure against our figure specifications.

Attachment Submitted filename: PONE-D-25-27701R1_Reviewer_Report.docx

# Author comment: Author response to Decision Letter 2

**DOI:** 10.1371/journal.pone.0359719.r004

13 Aug 2026

General response

Dear Academic Editor and Reviewers,

We sincerely thank you for the careful and constructive evaluation of our manuscript. We agree that the previous analytical framework did not adequately establish a clinically meaningful temporal relationship between oxygen measurement, catheter placement, and the thrombotic outcome, and that the reported 384-mmHg breakpoint was statistically unstable and clinically difficult to interpret.

Rather than retaining the previous framework, we substantially reconstructed the cohort and repeated the analyses. The revised exposure is the first arterial PaO₂ measurement obtained at or after insertion of a qualifying invasive venous catheter, within 24 hours after insertion, during the ICU stay, and while at least one qualifying catheter remained in situ. Because hospitalization-level diagnosis codes lack reliable thrombus-onset timestamps and cannot establish catheter causation, the outcome is no longer described as confirmed catheter-related thrombosis. It is now described as documented acute upper-body deep or central venous thrombosis during hospitalization.

We completely removed the segmented regression analysis, the 384-mmHg threshold, and all claims that higher PaO₂ has a protective effect. The reconstructed cohort included 11,277 hospital admissions from 10,789 patients, with 195 primary outcome events. In the fully adjusted model, PaO₂ was not associated with the outcome (OR per 10-mmHg increase, 1.008; 95% CI, 0.989–1.027; P = 0.430). The revised model includes catheter characteristics, the interval from catheter insertion to PaO₂ measurement, FiO₂, respiratory-support status, and non-respiratory organ dysfunction. Categorical, restricted cubic spline, alternative-outcome, PaO₂/FiO₂, subgroup, complete-case, and extreme-value sensitivity analyses were consistent with the primary result.

We also added the complete ICD code dictionary and outcome-classification rules in Supplementary Table S10, additional timing and upper-tail PaO₂ distribution data in Supplementary Table S11, and a completed STROBE checklist. The title, abstract, Methods, Results, Discussion, Conclusion, tables, figure captions, and Supporting Information have been extensively revised.

For clarity, the reviewer comments are summarized below in bold headings, followed by our responses and the corresponding locations of the revisions.

Reviewer #1

Comment 1. Temporal relationship between PaO₂ measurement, catheter placement, catheter dwell time, and thrombosis development

Reviewer comment: The temporal relationship remained insufficiently clarified. The reviewer requested detailed timing from ICU admission and catheter insertion to PaO₂ measurement, as well as from PaO₂ measurement to thrombosis diagnosis, and questioned whether a single early value could represent the relevant exposure for a later thrombotic event.

Response: We agree that the previous exposure definition was insufficient. We therefore reconstructed the cohort using qualifying catheter insertion as the index time. An eligible PaO₂ measurement was required to occur at or after catheter insertion, within 24 hours after insertion, during the corresponding ICU stay, and while at least one qualifying catheter remained active. The first measurement meeting all four criteria was selected.

We also characterized the timing of arterial blood-gas sampling. In an additional post hoc descriptive analysis, the median interval from ICU admission to the selected PaO₂ measurement was 299 minutes (IQR, 176–525) in admissions without the primary outcome and 494 minutes (IQR, 232–1,160) in admissions with the primary outcome. The corresponding intervals from catheter insertion to PaO₂ measurement were 45 minutes (IQR, 22–152) and 134 minutes (IQR, 60.5–339), respectively. The catheter-insertion-to-PaO₂ interval was included in the adjusted models after log transformation, and subgroup analyses across ≤1 hour, >1–6 hours, and >6–24 hours showed no evidence of interaction (P for interaction = 0.223).

However, the hospitalization-level diagnosis records do not provide a sufficiently reliable time of thrombus onset. We therefore cannot calculate a valid interval from PaO₂ measurement to thrombus formation, confirm that PaO₂ preceded thrombus formation in every admission, or calculate catheter dwell time before thrombus onset. For the same reason, a valid time-to-event or competing-risk analysis could not be performed. We have made these limitations explicit and no longer describe the outcome as confirmed catheter-related thrombosis.

Changes in the revised submission: Methods—Exposure assessment and Outcome definitions; Results—Study population and baseline characteristics; Table 1B; Supplementary Table S11; Discussion—limitations.

Comment 2. The 384-mmHg threshold was clinically uninterpretable and potentially driven by sparse data and model instability

Reviewer comment: The reviewer questioned the clinical relevance and statistical stability of the reported 384-mmHg breakpoint and requested information on the upper tail of the PaO₂ distribution and sensitivity analyses excluding extreme values.

Response: We agree. The segmented regression analysis, the 384-mmHg breakpoint, and all threshold-based claims have been removed from the title, abstract, Methods, Results, tables, figures, Discussion, and Conclusion.

For transparency, an additional post hoc descriptive summary showed that the reconstructed cohort contained 647 admissions and 10 primary-outcome events with PaO₂ >384 mmHg, 490 admissions and 5 events with PaO₂ >400 mmHg, and 111 admissions and 2 events above the 99th percentile (>461 mmHg). These counts confirm that the upper tail was sparse. Potential nonlinearity is now evaluated using restricted cubic splines, which showed no evidence of an overall association (P = 0.186) or nonlinearity (P = 0.109). Sensitivity analyses excluding PaO₂ >400 mmHg and values above the 99th percentile also yielded null estimates.

Changes in the revised submission: Threshold analysis removed throughout; Results—Sensitivity, nonlinear, and subgroup analyses; revised Fig 2; Supplementary Tables S1, S2, and S11.

Comment 3. Suitability of MIMIC-IV for the research question and need for validation using original clinical data

Reviewer comment: The reviewer suggested that, if the database cannot establish a valid exposure window and oxygenation context, the study question may require validation or re-conduction using original clinical data.

Response: We agree that MIMIC-IV cannot support a causal or time-to-event analysis of confirmed catheter-related thrombosis because reliable thrombus-onset timestamps, standardized imaging ascertainment, and several detailed catheter variables are unavailable. We therefore narrowed the research question to the association between a clearly defined early post-insertion PaO₂ measurement and a hospitalization-level documented upper-body thrombotic outcome.

Within this narrower scope, MIMIC-IV provides catheter insertion and removal records, arterial blood-gas timing, FiO₂, respiratory-support information, laboratory data, and illness-severity variables that permit a transparent observational analysis. We explicitly acknowledge the remaining temporal, exposure, and outcome-ascertainment limitations and recommend prospective validation using repeated oxygenation measurements, detailed catheter data, and accurately timed, imaging-confirmed thrombotic outcomes.

Changes in the revised submission: Title, objective, exposure definition, outcome terminology, Discussion, limitations, and Conclusion.

Reviewer #3

Comment 1. The estimate above 384 mmHg was non-interpretable because of complete separation or sparse events

Reviewer comment: The reviewer noted that OR = 0.00 with an infinite upper confidence limit indicated a non-estimable result rather than a true plateau.

Response: We agree. The previous estimate reflected sparse-data separation and should not have been interpreted as a plateau. The breakpoint model and every reference to 384 mmHg as a threshold have been removed. No threshold result remains in the revised manuscript. Upper-tail counts are reported only as an additional post hoc descriptive summary to demonstrate the sparsity that affected the previous model.

Changes in the revised submission: Threshold analysis removed from all manuscript sections, tables, and figures; Supplementary Table S11.

Comment 2. Report the continuous association per a clinically meaningful increment and temper the interpretation

Reviewer comment: The reviewer recommended reporting the OR per 10 mmHg rather than per 1 mmHg and avoiding overstatement of a near-null effect.

Response: PaO₂ is now reported per 10-mmHg increase throughout the manuscript. The unadjusted estimate was OR 0.970 (95% CI, 0.952–0.987), but the association attenuated after adjustment. The fully adjusted estimate was OR 1.008 (95% CI, 0.989–1.027; P = 0.430). We therefore no longer describe the result as a significant inverse association, risk reduction, or protective effect.

Changes in the revised submission: Abstract; Methods—Statistical analysis; Results; Tables 2 and 3; Discussion; Conclusion.

Comment 3. Account for FiO₂, ventilation, and the PaO₂/FiO₂ ratio

Reviewer comment: The reviewer noted that PaO₂ partly reflects oxygen treatment and recommended adjustment for FiO₂ and ventilation and analysis of the PaO₂/FiO₂ ratio.

Response: We agree that PaO₂ cannot be interpreted independently of oxygen delivery and respiratory support. The fully adjusted model now includes FiO₂ at the time of PaO₂ measurement, respiratory-support status, and the non-respiratory component of the first-day SOFA score. Respiratory support was classified as invasive mechanical ventilation, other recorded respiratory support, or no contemporaneous respiratory-support interval identified.

We also analyzed the PaO₂/FiO₂ ratio as an alternative exposure. It was not associated with the outcome (OR per 50-unit increase, 1.010; 95% CI, 0.957–1.065; P = 0.730). A high-reliability FiO₂ sensitivity analysis restricted FiO₂ to the same blood-gas record or a ventilator setting recorded within 60 minutes of PaO₂ measurement and also yielded a null result.

Changes in the revised submission: Methods—Covariates and Statistical analysis; Results—Sensitivity analyses; Supplementary Tables S1–S3.

Comment 4. Outcome underascertainment and differential detection bias

Reviewer comment: The reviewer requested the total number of thrombotic events and discussion of possible underascertainment and differential imaging or documentation.

Response: We agree. The revised primary outcome occurred in 195 of 11,277 admissions (1.73%): 32 events in the PaO₂ ≤80-mmHg group, 29 in the 81–100-mmHg group, and 134 in the >100-mmHg group. We removed the term confirmed catheter-related thrombosis. The primary outcome is now restricted to documented acute upper-body deep or central venous thrombosis, whereas superficial and anatomically unspecified diagnoses are excluded.

A broader outcome included 282 events, and a strict laterality-compatible deep or central venous outcome included 69 events; both yielded null adjusted PaO₂ estimates. For the strict definition, a right-sided diagnosis required a right-sided qualifying catheter, a left-sided diagnosis required a left-sided qualifying catheter, and a bilateral diagnosis was considered compatible; diagnoses with unspecified laterality were excluded. We now explicitly acknowledge that asymptomatic or clinically unsuspected events may have been missed and that outcome detection may have varied according to diagnostic testing and documentation. If patients with greater illness severity or lower PaO₂ were imaged more frequently, differential ascertainment could have contributed to the apparent inverse association in the less-adjusted models.

Changes in the revised submission: Methods—Outcome definitions; Results—event counts and alternative outcomes; Table 1A; Supplementary Tables S3 and S10; Discussion—limitations.

Comment 5. A single PaO₂ value cannot capture dynamic exposure; a time-varying model would be preferable

Reviewer comment: The reviewer emphasized that one baseline value does not represent oxygenation over the ICU stay and raised concern about differing exposure windows and time-related bias.

Response: We agree that a single early PaO₂ measurement cannot represent cumulative or time-varying oxygen exposure. The revised research question is explicitly limited to the association of one early post-insertion measurement with a hospitalization-level documented outcome. We do not interpret the selected value as a measure of cumulative hypoxemia or hyperoxemia.

A time-varying Cox or competing-risk model could not be implemented validly because reliable thrombus-onset timestamps were unavailable. The revised Discussion explicitly acknowledges the single-measurement limitation, temporal uncertainty, selection related to requiring an eligible arterial blood-gas measurement, and the inability to perform valid time-to-event analyses. Future studies should use repeated or time-weighted oxygenation measurements and accurately timed, imaging-confirmed outcomes.

Changes in the revised submission: Methods—Exposure assessment; Discussion—limitations; Conclusion.

Comment 6. Improve STROBE reporting, including missing data, event counts, and model performance

Reviewer comment: The reviewer requested the number excluded for missing variables, comparison of missing cases, event counts by PaO₂ group, and calibration/discrimination information.

Response: Covariate missingness is now handled using multiple imputation rather than excluding admissions solely for missing prespecified covariates. Supplementary Table S6 reports observed and missing counts, missingness percentages, imputation methods, and imputation diagnostics. A complete-case sensitivity analysis included 8,906 admissions and 139 events.

Event counts by PaO₂ category are reported in the Results and Table 1A. Supplementary Table S5 reports the mean apparent AUC (0.7834), mean apparent Brier score (0.0166), generalized variance inflation factors, leverage, Cook’s distance, DFBETA, and an influence analysis. Supplementary Figure S2 presents apparent calibration and is explicitly described as uncorrected for optimism and not as internal or external validation. A completed STROBE checklist is submitted separately.

Changes in the revised submission: Fig 1; Results; Tables 1A–1B; Supplementary Tables S5–S6; Supplementary Figure S2; S1 Checklist.

Comment 7. The biological rationale relied too heavily on COVID-19 cohorts

Reviewer comment: The reviewer requested acknowledgment that COVID-19-associated coagulopathy may not generalize to an unselected ICU population and recommended broader evidence.

Response: We agree. The biological rationale and Discussion were rewritten and no longer rely on COVID-19 cohorts. The revised text considers intermittent hypoxia in obstructive sleep apnea, environmental hypoxia, COPD-associated thromboembolism, perioperative hyperoxia, and randomized ICU oxygen-target trials. We also emphasize that these settings cannot be directly extrapolated to a single routine ICU PaO₂ measurement.

Changes in the revised submission: Introduction and Discussion.

Comment 8. Table 1 contained misaligned or internally inconsistent cells

Reviewer comment: The reviewer requested verification of values, units, and decimal places and improvement of table alignment.

Response: Table 1 was reformatted, and the values, denominators, units, and decimal places were checked. To improve readability, the three pairwise standardized mean-difference columns were removed from the main Table 1A and transferred to Supplementary Table S9.

Changes in the revised submission: Reformatted Tables 1A–1B; new Supplementary Table S9.

Comment 9. Standardize PO₂ and PaO₂ notation

Reviewer comment: The reviewer requested consistent use of PaO₂ for arterial oxygen tension.

Response: The terminology has been standardized to PaO₂ throughout the title, abstract, main text, tables, figure captions, and supporting information.

Changes in the revised submission: All manuscript and supporting-information files.

Comment 10. Figure 2 should report the sample size and show the PaO₂ distribution

Reviewer comment: The reviewer requested the number of observations and a visual indication of the exposure distribution because of wide confidence intervals at the extremes.

Response: The revised restricted cubic spline figure includes rug marks showing the observed PaO₂ distribution and displays the 95% confidence interval across the plotted range. The caption now states that the analysis included 11,277 hospital admissions and 195 outcome events. Supplementary Table S11 also reports the overall PaO₂ distribution and upper-tail counts.

Changes in the revised submission: Revised Fig 2 and caption; Supplementary Table S11.

Reviewer #4

Major Comment 1. Fundamental concern regarding outcome ascertainment

Reviewer comment: The reviewer requested the exact ICD codes, validation strategy, sensitivity and specificity, anatomical restriction, and an explanation of how unrelated DVTs were excluded.

Response: We agree that the previous terminology overstated what could be established from the available data. The revised outcome is not described as confirmed catheter-related thrombosis. It is now defined as documented acute upper-body deep or central venous thrombosis during hospitalization. The revised algorithm uses the MIMIC-IV diagnoses_icd table and no longer relies on clinical notes.

The primary definition includes acute ICD-9-CM and ICD-10-CM codes for deep upper-extremity, axillary, subclavian, and internal jugular venous thrombosis. Superficial, chronic, and anatomically unspecified diagnoses are excluded from the primary definition. A broader acute outcome and a strict laterality-compatible deep or central venous outcome were evaluated as sensitivity analyses. For the strict definition, right-sided diagnoses were required to match a right-sided qualifying catheter, left-sided diagnoses were required to match a left-sided qualifying catheter, bilateral diagnoses were considered compatible, and diagnoses with unspecified laterality were excluded. The complete code list and classification rules are provided in Supplementary Table S10.

No chart-adjudicated or imaging-adjudicated reference standard was available; therefore, we cannot estimate or claim the sensitivity or specificity of the coding algorithm. We also cannot exclude every non-catheter-related upper-body thrombosis or establish catheter causation. These limitations are now stated explicitly.

Changes in the revised submission: Methods—Outcome definitions; Supplementary Tables S3 and S10; Discussion—limitations.

Major Comment 2. The exposure definition based on a single first PaO₂ measurement was problematic

Reviewer comment: The reviewer asked how soon PaO₂ was measured, whether thrombosis may already have been present, and why time-weighted, maximum, minimum, or cumulative measures were not used.

Response: The exposure is no longer the first PaO₂ recorded during the ICU stay. It is the first PaO₂ obtained at or after qualifying catheter insertion, within 24 hours, during the corresponding ICU stay, and while the catheter remained active. This definition provides a reproducible early post-insertion measurement and avoids using later measurements that may be increasingly affected by the subsequent ICU course.

We agree that the selected value remains a single measurement and cannot represent time-weighted, maximum, minimum, or cumulative oxygen exposure. Reliable thrombus-onset timestamps were unavailable, so repeated oxygen measurements could not be aligned with event onset, and pre-existing thrombosis at the time of the selected measurement cannot be excluded in every admission. These limitations are now stated explicitly. The timing from ICU admission and catheter insertion to the selected PaO₂ is reported in the Results and Supplementary Table S11.

Changes in the revised submission: Methods—Exposure assessment and Outcome definitions; Results—timing description; Supplementary Table S11; Discussion—limitations.

Major Comment 3. Temporal relationship remained unclear; Cox or competing-risk analysis was suggested

Reviewer comment: The reviewer requested catheter insertion time, PaO₂ time, and thrombosis time and suggested time-to-event methods.

Response: Exact catheter insertion and selected PaO₂ times were available and are now linked explicitly. The interval from catheter insertion to PaO₂ was incorporated into the adjusted models, and timing by outcome status is reported. However, thrombosis was available only as a hospitalization-level diagnosis without a sufficiently reliable onset time. Using a diagnosis-code date as biological onset would create false precision.

We therefore retained hospitalization-level logistic regression, clearly limited the estimand to an association with a documented hospitalization-level outcome, and removed claims that PaO₂ preceded thrombus formation. We now state explicitly that valid Cox, time-to-event, and competing-risk analyses could not be performed.

Changes in the revised submission: Methods—Exposure assessment and Outcome definitions; Results; Supplementary Table S11; Discussion—limitations.

Major Comment 4. Severe confounding by illness severity; propensity-score or weighting methods were recommended

Reviewer comment: The reviewer noted major baseline differences and recommended propensity-score matching, IPTW, or doubly robust analyses.

Response: We agree that illness severity and treatment context are important sources of confounding. The revised fully adjusted model includes age, sex, Charlson Comorbidity Index, hemoglobin, platelet count, lactate, catheter type, catheter laterality, multiple active catheters, the interval from catheter insertion to PaO₂ measurement, FiO₂, respiratory-support status, and the non-respiratory component of the first-day SOFA score. The attenuation of the inverse estimate after catheter, timing, oxygenation, and severity variables were added is reported transparently.

We considered the suggested propensity-score methods. However, PaO₂ is a continuous physiologic measurement rather than an assigned binary treatment. Conventional matching or binary-treatment IPTW would require an arbitrary PaO₂ cutoff, discard exposure information, and change the estimand. These approaches would also not resolve unavailable thrombus-onset times or unmeasured confounding. We therefore retained prespecified continuous and categorical analyses with sequential multivariable adjustment, multiple imputation, patient-clustered robust standard errors, nonlinear analysis, and extensive sensitivity analyses. The manuscript consistently interprets all estimates as associations rather than causal effects.

Changes in the revised submission: Methods—Covariates and Statistical analysis; Results—sequential and module-specific models; Discussion—interpretation and limitations.

Major Comment 5. Potential reverse causality

Reviewer comment: The reviewer suggested that illness severity could cause both hypoxemia and thrombosis rather than PaO₂ causing thrombosis and requested more cautious interpretation.

Response: We agree. The revised models account for available measures of illness severity, respiratory treatment, laboratory status, catheter characteristics, and measurement timing. The inverse estimate observed in the unadjusted and minimally adjusted models moved to the null after these variables were included. We now interpret this attenuation as evidence that the crude association was sensitive to clinical and treatment context, not as evidence of an independent or causal effect of PaO₂.

Because thrombus-onset time was unavailable, reverse temporal ordering cannot be excluded. This limitation is stated explicitly, and all causal terminology has been removed.

Changes in the revised submission: Results—sequential and module-specific analyses; Discussion—interpretation and limitations; Conclusion.

Major Comment 6. The threshold analysis was statistically fragile

Reviewer comment: The reviewer questioned the biological plausibility and statistical stability of the 384-mmHg threshold, noting the likely sparsity of observations in the upper tail and the non-estimable estimate above the breakpoint.

Response: We agree. The segmented regression analysis, the 384-mmHg breakpoint, and all threshold-based claims have been removed from the title, abstract, Methods, Results, tables, figures, Discussion, and Conclusion. In an additional post hoc descriptive summary, 647 admissions with 10 primary-outcome events had PaO₂ values greater than 384 mmHg, 490 admissions with 5 events had values greater than 400 mmHg, and 111 admissions with 2 events had values above the 99th percentile (>461 mmHg). These counts confirmed the sparsity of the upper tail. Potential nonlinearity is now evaluated using restricted cubic splines, which showed no evidence of an overall association (P = 0.186) or nonlinearity (P = 0.109). Sensitivity analyses excluding PaO₂ values greater than 400 mmHg and values above the 99th percentile also yielded null estimates.

Changes in the revised submission: Threshold analysis removed throughout; Results—Sensitivity, nonlinear, and subgroup analyses; Fig 2; Supplementary Tables S1, S2, and S11.

Major Comment 7. Inconsistency between continuous and categorical analyses

Reviewer comment: The reviewer noted discordance between a near-null continuous estimate and substantially lower categorical odds.

Response: The discrepancy is no longer present after cohort reconstruction and comprehensive adjustment. In the fully adjusted model, the continuous estimate was OR 1.008 (95% CI, 0.989–1.027) per 10-mmHg increase. The categorical estimates were OR 0.900 (95% CI, 0.531–1.526) for 81–100 versus ≤80 mmHg and OR 0.989 (95% CI, 0.652–1.502) for >100 versus ≤80 mmHg. Restricted cubic spline analysis also showed no overall or nonlinear association.

Changes in the revised submission: Results; Tables 2–3; Fig 2.

Major Comment 8. Anticoagulation may introduce overadjustment or collider bias

Reviewer comment: The reviewer questioned adjustment for anticoagulant administration because its timing and indication may be related to perceived thrombotic risk.

Response: We agree that time-varying anticoagulation is difficult to position analytically when indication and timing relative to exposure and outcome are uncertain. Anticoagulant administration is not included in the revised primary adjustment model. The Discussion now acknowledges that detailed time-varying information on thromboprophylaxis and therapeutic anticoagulation was limited, leaving potential residual confounding. We did not make causal claims about the role of anticoagulation.

Changes in the revised submission: Methods—Covariate specification; Discussion—limitations.

Major Comment 9. Important catheter and clinical risk factors were missing

Reviewer comment: The reviewer listed catheter type, lumens, size, dwell time, tip position, ventilation, cancer, sepsis, platelet count, D-dimer, and coagulation variables.

Response: The revised analysis incorporates catheter type, catheter laterality, multiple active qualifying catheters, platelet count, hemoglobin, lactate, FiO₂, respiratory-support status, Charlson Comorbidity Index, and non-respiratory SOFA. PICC status was also evaluated in subgroup analysis.

Nevertheless, catheter material, exact diameter, catheter-to-vein ratio, exact tip position, insertion technique, operator-related factors, complete dwell time before thrombus onset, D-dimer, detailed coagulation measures, and detailed time-varying anticoagulation were unavailable or could not be measured reliably. These limitations are now stated explicitly. The associations of individual covariates are presented as secondary, hypothesis-generating findings and are not described as independent risk factors.

Changes in the revised submission: Methods—Covariates; Results—secondary covariate associations; Supplementary Table S8; Discussion—limitations.

Major Comment 10. Causal wording such as “protective effect” and “risk reduction” should be removed

Reviewer comment: The reviewer requested consistent noncausal terminology.

Response: We agree. Terms including protective effect, risk reduction, and higher PaO₂ reduces thrombosis have been removed. The revised manuscript consistently uses association, odds, and documented outcome. The Conclusion states that the findings do not support PaO₂-based thrombosis risk stratification or modification of oxygen therapy for thrombosis prevention.

Changes in the revised submission: Title; Abstract; Results; Discussion; Conclusion.

Minor Comment 1. Use one study-design term consistently

Reviewer comment: The reviewer noted inconsistent use of retrospective cohort and cross-sectional terminology.

Response: The study is now consistently described as a retrospective observational cohort study. Cross-sectional terminology has been removed.

Changes in the revised submission: Title, Abstract, Methods, and Discussion.

Minor Comment 2. Standardize PO₂ and PaO₂

Reviewer comment: The reviewer requested consistent arterial oxygen terminology.

Response: PaO₂ is now used consistently throughout the manuscript and supporting information.

Changes in the revised submission: All files.

Minor Comment 3. Clarify APSIII versus APACHE III

Reviewer comment: The reviewer requested clarification regarding the severity score used in the models.

Response: Neither APSIII nor APACHE III is included in the revised primary models. Illness severity is represented by the non-respiratory component of the first-day SOFA score, which is consistently defined and reported.

Changes in the revised submission: Methods—Covariates and Statistical analysis; tables and supporting information.

Minor Comment 4. Report continuous ORs per a meaningful increment

Reviewer comment: The reviewer recommended reporting the continuous association per 10 mmHg or per standard deviation.

Response: All continuous PaO₂ associations are now reported per 10-mmHg increase.

Changes in the revised submission: Abstract; Methods; Results; tables; figures; supporting information.

Minor Comment 5. Report handling of missing data

Reviewer comment: The reviewer requested the number and percentages of missing variables and consideration of multiple imputation.

Response: Missing values in prespecified model covariates were handled using multiple imputation by chained equations with 20 imputed datasets. Supplementary Table S6 reports observed and missing counts, missingness percentages, imputation methods, and diagnostics. Supplementary Figures S3 and S4 provide graphical imputation diagnostics. A complete-case sensitivity analysis was also performed.

Changes in the revised submission: Methods—Statistical analysis; Supplementary Table S6; Supplementary Figures S3–S4.

Minor Comment 6. Provide the MIMIC-IV version and reproducible extraction and analysis code

Reviewer comment: The reviewer requested the exact database version, SQL extraction strategy, and a reproducible code repository or package.

Response: Thank you for this important suggestion. The database version is now specified as MIMIC-IV v2.2, and the Methods and Supporting Information have been expanded to describe the cohort-construction logic, eligibility criteria, exposure window, outcome definitions, ICD code list, covariate definitions, missing-data procedures, model specifications, and sensitivity analyses. We agree that sharing well-documented analysis code would further improve reproducibility. The reported results were generated using the final analytic workflow; however, the relevant code currently remains distributed across several working scripts and has not yet been consolidated and documented as a single publication-ready repository. To avoid providing an incomplete or potentially misleading package, we have not included executable code with this revision. If the Editor considers code submission essential for further evaluation, we would be grateful for the opportunity to prepare and provide a fully consolidated and documented package. Patient-level MIMIC-IV data are not redistributed because they are subject to the PhysioNet Credentialed Health Data Use Agreement; qualified researchers may obtain the data through the same credentialing process, and the authors had no special access privileges.

Changes in the revised submission: Methods—Data source and Statistical analysis; Supporting Information; Data Availability Statement in the submission system.

Closing statement

We again thank the Academic Editor and Reviewers for their detailed and constructive comments. The review prompted a fundamental reconstruction of the study, removal of the unstable threshold claim, more complete adjustment for catheter and oxygenation context, and a substantially more cautious interpretation. We believe that the revised manuscript now addresses the concerns transparently and reports the null fully adjusted result without causal or threshold-based claims.

Sincerely,

Qing Cui, on behalf of all auth

Attachment Submitted filename: renamed_f23a0.docx

# Peer review: Decision Letter 2

**DOI:** 10.1371/journal.pone.0359719.r005

**Authors:** Giovanni Giordano

10 Sep 2026

Dear Dr. Cui,

Thank you for submitting your manuscript to PLOS One. After careful consideration, we feel that it has merit but does not fully meet PLOS One’s publication criteria as it currently stands. Therefore, we invite you to submit a revised version of the manuscript that addresses the points raised during the review process.

Please submit your revised manuscript by Oct 25 2026 11:59PM. If you will need more time than this to complete your revisions, please reply to this message or contact the journal office at plosone@plos.org. When you're ready to submit your revision, log on to https://www.editorialmanager.com/pone/ and select the 'Submissions Needing Revision' folder to locate your manuscript file.

- A letter that responds to each point raised by the academic editor and reviewer(s). You should upload this letter as a separate file labeled 'Response to Reviewers'.
- A marked-up copy of your manuscript that highlights changes made to the original version. You should upload this as a separate file labeled 'Revised Manuscript with Track Changes'.
- An unmarked version of your revised paper without tracked changes. You should upload this as a separate file labeled 'Manuscript'.

If you would like to make changes to your financial disclosure, please include your updated statement in your cover letter. Guidelines for resubmitting your figure files are available below the reviewer comments at the end of this letter.

If applicable, we recommend that you deposit your laboratory protocols in protocols.io to enhance the reproducibility of your results. Protocols.io assigns your protocol its own identifier (DOI) so that it can be cited independently in the future. For instructions see: https://journals.plos.org/plosone/s/submission-guidelines#loc-laboratory-protocols. Additionally, PLOS One offers an option for publishing peer-reviewed Lab Protocol articles, which describe protocols hosted on protocols.io. Read more information on sharing protocols at https://plos.org/protocols?utm_medium=editorial-email&utm_source=authorletters&utm_campaign=protocols.

As the corresponding author, your ORCID iD is verified in the submission system and will appear in the published article. PLOS supports the use of ORCID, and we encourage all coauthors to register for an ORCID iD and use it as well. Please encourage your coauthors to verify their ORCID iD within the submission system before final acceptance, as unverified ORCID iDs will not appear in the published article. *Only*  the individual author can complete the verification step; PLOS staff *cannot*  verify ORCID iDs on behalf of authors.

We look forward to receiving your revised manuscript.

Kind regards,

Giovanni Giordano

Academic Editor

PLOS One

Journal Requirements:

1. If the reviewer comments include a recommendation to cite specific previously published works, please review and evaluate these publications to determine whether they are relevant and should be cited. There is no requirement to cite these works unless the editor has indicated otherwise.

2. Please review your reference list to ensure that it is complete and correct. If you have cited papers that have been retracted, please include the rationale for doing so in the manuscript text, or remove these references and replace them with relevant current references. Any changes to the reference list should be mentioned in the rebuttal letter that accompanies your revised manuscript. If you need to cite a retracted article, indicate the article’s retracted status in the References list and also include a citation and full reference for the retraction notice.

Additional Editor Comments:

Dear Dr. Cui and colleagues,

Thank you for submitting the revised version of your manuscript, “Association between early post-insertion arterial oxygen tension and documented acute upper-body deep or central venous thrombosis in critically ill adults: a retrospective MIMIC-IV cohort study” (PONE-D-25-27701R2).

The manuscript has undergone substantial improvement. In particular, the reconstructed cohort, removal of the previously proposed PaO₂ threshold and protective-effect interpretation, revised outcome definition, expanded adjustment strategy, and extensive sensitivity analyses have addressed the major methodological concerns raised during the previous rounds. The revised analysis now reports a well-estimated null association between early post-insertion PaO₂ and the documented thrombotic outcome.

A small number of issues should nevertheless be addressed before the manuscript can be considered for acceptance.

1. Timing between catheter insertion and PaO₂ measurement

The interval between catheter insertion and the selected PaO₂ measurement differs substantially according to outcome status (median 45 vs 134 minutes), and the timing variable itself is associated with the documented thrombotic outcome. Although this interval has appropriately been included in the adjusted models and subgroup analyses according to timing showed no significant interaction, these analyses do not fully address the potential selection and temporal ambiguity introduced by the exposure window.

Please therefore provide an additional sensitivity analysis restricting the cohort to a narrow early post-insertion measurement window, preferably PaO₂ measurements obtained within 1 hour after catheter insertion. Please report the number of included admissions and events and the corresponding adjusted PaO₂ estimate. If another narrow window is considered more appropriate based on the data distribution, please justify the choice.

The related limitation should also be described in terms of temporal uncertainty and possible selection related to measurement timing. Because the exact thrombus-onset time is unavailable, the manuscript should avoid implying that a conventional immortal-time bias can be definitively established.

2. Role of FiO₂ in the adjustment strategy

The Reviewer raised the possibility that adjustment for FiO₂ could contribute to over-adjustment because FiO₂ is a determinant of the measured PaO₂. However, the manuscript already presents Model 2, which does not include FiO₂, respiratory-support status, or non-respiratory SOFA and still shows no association between PaO₂ and the outcome (OR 1.003, 95% CI 0.984–1.022; P = 0.774).

No additional model therefore appears necessary. Please make this point explicit in the response letter and briefly acknowledge in the manuscript that the interpretation of adjustment for FiO₂ depends on the estimand and that the null finding was already present before inclusion of the oxygenation/severity variables. The PaO₂/FiO₂ analysis should continue to be described as an alternative-exposure analysis rather than as an adjustment variable in the primary model.

3. Study-design terminology and temporal ordering

The manuscript is currently described as a retrospective observational cohort study. Given that the exact onset of thrombosis cannot be established and that it cannot be confirmed that the selected PaO₂ measurement preceded thrombus formation in every admission, please further clarify this temporal limitation in the Methods and/or Limitations.

The current study-design terminology may be retained if adequately qualified, although “retrospective observational study” would also be acceptable if the Authors prefer a more conservative formulation.

4. Outcome ascertainment

The manuscript already appropriately acknowledges the limitations of ICD-based outcome ascertainment, including the lack of standardized prospective imaging, unknown sensitivity and specificity of the coding algorithm, possible under-detection of asymptomatic events, and differential ascertainment.

Please add a brief explicit statement noting that the observed event rate may therefore be lower than rates reported in studies using systematic imaging surveillance.

Overall, the revised manuscript is now close to being suitable for publication. Please submit a final revised version addressing the points above together with a concise point-by-point response.

Kind regards,

Giovanni Giordano

[Note: HTML markup is below. Please do not edit.]

Reviewers' comments:

Reviewer's Responses to Questions

**Comments to the Author**

Reviewer #3: (No Response)

Reviewer #4: All comments have been addressed

**********

2. Is the manuscript technically sound, and do the data support the conclusions?

Reviewer #3: Partly

Reviewer #4: (No Response)

**********

3. Has the statistical analysis been performed appropriately and rigorously?

Reviewer #3: Yes

Reviewer #4: (No Response)

**********

4. Have the authors made all data underlying the findings in their manuscript fully available?

The PLOS Data policy

Reviewer #3: Yes

Reviewer #4: (No Response)

**********

5. Is the manuscript presented in an intelligible fashion and written in standard English?

Reviewer #3: Yes

Reviewer #4: (No Response)

**********

Reviewer #3: Dear Editor and Authors,

Thank you for the opportunity to review this manuscript entitled "Association between early post-insertion arterial oxygen tension and documented acute upper-body deep or central venous thrombosis in critically ill adults: a retrospective MIMIC-IV cohort study" (Manuscript ID: PONE-D-25-27701). This is the third review of the manuscript. I am grateful to the authors for the substantial work invested in this revision.

In this study the authors use the MIMIC-IV database to examine whether early post-insertion arterial oxygen tension (PaO2) is associated with documented acute upper-body deep or central venous thrombosis in critically ill adults. Compared with the previous versions, the cohort has been entirely reconstructed (11,277 admissions, 10,789 patients, 195 events, 1.73%), the previously reported 384-mmHg threshold and all "protective" claims have been removed, and the analysis now yields a well-estimated null association (fully adjusted OR 1.008 per 10 mmHg, 95% CI 0.989-1.027, P=0.43), supported by categorical models, a PaO2/FiO2 analysis, restricted cubic splines, multiple imputation, and model-performance and influence diagnostics. I commend the authors; this revision resolves the complete-separation problem and the over-interpretation that affected the original submission, and the manuscript is now scientifically sound and, for a null finding, clearly reportable.

A few points should still be addressed to strengthen the validity and transparency of the work before publication.

MAJOR REVISIONS

1. The interval between catheter insertion and the first PaO2 measurement differs systematically between patients with and without the outcome (approximately 134 vs 45 minutes) and is itself a strong predictor in the models (OR 1.33 per unit, P<0.001). This raises a concern of immortal-time-like/selection bias that adjustment alone may not fully remove, because patients who develop the outcome have a different temporal profile. The authors should discuss this explicitly and provide a sensitivity analysis restricting the cohort to a narrow, homogeneous insertion-to-measurement window (e.g., excluding extreme intervals) to show that the null result is robust to this design feature.

2. FiO2 (and the PaO2/FiO2 ratio) is a determinant of the exposure rather than a classical confounder. Adjusting for it may attenuate any true association toward the null through over-adjustment effects. Because the primary conclusion is a null association, this deserves an explicit acknowledgment, and the authors should present a model that excludes FiO2 and the PaO2/FiO2 term, so that readers can judge whether the null persists without conditioning on a determinant of the exposure.

MINOR REVISIONS

3. Given that the exposure is captured at a single time point and that the design cannot establish that exposure precedes the outcome, the label "cohort study" is somewhat generous. The authors should either soften this terminology and add a brief statement in the Methods/Limitations clarifying the temporal relationship between exposure measurement and outcome ascertainment.

4. Outcome ascertainment relies on administrative ICD coding, which is known to under-capture asymptomatic thrombosis. The authors have honestly disclosed this and added broad and strict outcome sensitivity analyses, both null. I would only ask that the Limitations section state plainly that residual outcome misclassification remains possible and that the observed event rate is lower than rates reported with systematic screening.

In summary, the authors have thoroughly and honestly addressed the concerns raised in the previous rounds; the reconstructed analysis is rigorous and the null finding is appropriately presented. The remaining requests concern the discussion of temporal/selection bias, transparency around adjustment for a determinant of the exposure, and minor clarifications of terminology and outcome ascertainment. I believe the manuscript can be accepted after these points are addressed.

Best regards,

The reviewer

Reviewer #4: (No Response)

**********

what does this mean?). If published, this will include your full peer review and any attached files.

If you choose “no”, your identity will remain anonymous but your review may still be made public.

**Do you want your identity to be public for this peer review?**  For information about this choice, including consent withdrawal, please see our Privacy Policy

Reviewer #3: No

Reviewer #4: No

**********

[NOTE: If reviewer comments were submitted as an attachment file, they will be attached to this email and accessible via the submission site. Please log into your account, locate the manuscript record, and check for the action link "View Attachments". If this link does not appear, there are no attachment files.]

To ensure your figures meet our technical requirements, please review our figure guidelines: https://journals.plos.org/plosone/s/figures

You may also use PLOS’s free figure tool, NAAS, to help you prepare publication quality figures: https://journals.plos.org/plosone/s/figures#loc-tools-for-figure-preparation.

NAAS will assess whether your figures meet our technical requirements by comparing each figure against our figure specifications.

Attachment Submitted filename: PONE-D~2.DOC

# Author comment: Author response to Decision Letter 3

**DOI:** 10.1371/journal.pone.0359719.r006

12 Sep 2026

Dear Academic Editor and Reviewers,

Thank you for your careful and constructive comments. We have addressed all remaining points raised by the Academic Editor and Reviewer #3. The revision includes an additional sensitivity analysis restricted to PaO₂ measurements obtained within 60 minutes after catheter insertion, clarification of the role of FiO₂ in the adjustment strategy, further qualification of the temporal relationship between PaO₂ measurement and thrombosis ascertainment, and additional discussion of limitations related to ICD-based outcome ascertainment.

A detailed point-by-point response to each comment is provided in the separately uploaded Response to Reviewers document. All corresponding changes have been incorporated into the revised manuscript and Supporting Information.

We sincerely thank the Academic Editor and Reviewers for their guidance.

Attachment Submitted filename: Response_to_Reviewers_auresp_3.docx

# Editor report: Decision Letter 3

**DOI:** 10.1371/journal.pone.0359719.r007

**Authors:** Giovanni Giordano

17 Sep 2026

Association between early post-insertion arterial oxygen tension and documented acute upper-body deep or central venous thrombosis in critically ill adults: a retrospective MIMIC-IV cohort study

PONE-D-25-27701R3

Dear Dr. Cui,

We’re pleased to inform you that your manuscript has been judged scientifically suitable for publication and will be formally accepted for publication once it meets all outstanding technical requirements.

Within one week, you’ll receive an e-mail detailing the required amendments. When these have been addressed, you’ll receive a formal acceptance letter and your manuscript will be scheduled for publication.

An invoice will be generated when your article is formally accepted. Please note, if your institution has a publishing partnership with PLOS and your article meets the relevant criteria, all or part of your publication costs will be covered. Please make sure your user information is up-to-date by logging into Editorial Manager at Editorial Manager® and clicking the ‘Update My Information' link at the top of the page. For questions related to billing, please contact billing support.

If your institution or institutions have a press office, please notify them about your upcoming paper to help maximize its impact. If they’ll be preparing press materials, please inform our press team as soon as possible -- no later than 48 hours after receiving the formal acceptance. Your manuscript will remain under strict press embargo until 2 pm Eastern Time on the date of publication. For more information, please contact onepress@plos.org.

Kind regards,

Giovanni Giordano

Academic Editor

PLOS One

Additional Editor Comments (optional):

Reviewers' comments:

# Editor report: Acceptance letter

**DOI:** 10.1371/journal.pone.0359719.r008

**Authors:** Giovanni Giordano

PONE-D-25-27701R3

PLOS One

Dear Dr. Cui,

I'm pleased to inform you that your manuscript has been deemed suitable for publication in PLOS One. Congratulations! Your manuscript is now being handed over to our production team.

At this stage, our production department will prepare your paper for publication. This includes ensuring the following:

* All references, tables, and figures are properly cited

* All relevant supporting information is included in the manuscript submission,

* There are no issues that prevent the paper from being properly typeset

You will receive further instructions from the production team, including instructions on how to review your proof when it is ready. Please keep in mind that we are working through a large volume of accepted articles, so please give us a few days to review your paper and let you know the next and final steps.

Lastly, if your institution or institutions have a press office, please let them know about your upcoming paper now to help maximize its impact. If they'll be preparing press materials, please inform our press team within the next 48 hours. Your manuscript will remain under strict press embargo until 2 pm Eastern Time on the date of publication. For more information, please contact onepress@plos.org.

You will receive an invoice from PLOS for your publication fee after your manuscript has reached the completed accept phase. If you receive an email requesting payment before acceptance or for any other service, this may be a phishing scheme. Learn how to identify phishing emails and protect your accounts at https://explore.plos.org/phishing.

If we can help with anything else, please email us at customercare@plos.org.

Thank you for submitting your work to PLOS One and supporting open access.

Kind regards,

PLOS One Editorial Office Staff

on behalf of

Dr. Giovanni Giordano

Academic Editor

PLOS One
